# Synthesis of glyceryl glycosides related to A-type prymnesin toxins

**DOI:** 10.1016/j.carres.2018.04.008

**Published:** 2018-06-30

**Authors:** Edward S. Hems, Sergey A. Nepogodiev, Martin Rejzek, Robert A. Field

**Affiliations:** Department of Biological Chemistry, John Innes Centre, Norwich Research Park, Norwich, NR4 7UH, UK

**Keywords:** Algae, *Prymnesium parvum*, Toxins, Prymnesins, Glyceryl glycosides

## Abstract

A suite of glycosylated glycerol derivatives representing various fragments of the glycosylated ichthyotoxins called prymnesins were chemically synthesised. Glycerol was used to represent a small fragment of the prymnesin backbone, and was glycosylated at the 2° position with the sugars currently reported to be present on prymnesin toxins. Neighbouring group participation was utilised to synthesise 1,2-*trans*-glycosides. SnCl_2_-promoted glycosylation with furanosyl fluorides gave 1,2-*cis*-furanosides with moderate stereocontrol, whilst TMSOTf promoted glycosylation with a furanosyl imidate gave a 1,2-*cis*-furanoside with good stereocontrol. The chemical synthesis of two larger glyceryl diglycoside fragments of prymnesin-1, glycosylated with α-ʟ-arabinopyranose and α-ᴅ-ribofuranose, is also described. As the stereochemistry of the prymnesin backbones at this region is undefined, both the 2*R*- and 2*S-* glycerol isomers were synthesised. The separated diastereoisomers were distinguished by comparing NOESY NMR with computational models.

## Highlights

• Synthesis of glyceryl glycosides inspired by carbohydrates on A-type prymnesins.
• Di-glycosylated fragments of prymnesin-1 synthesised for bioconjugation studies.
• NOESY NMR compared with computational models to assign fragment stereochemistry.

## Graphical abstract

**Figure:**
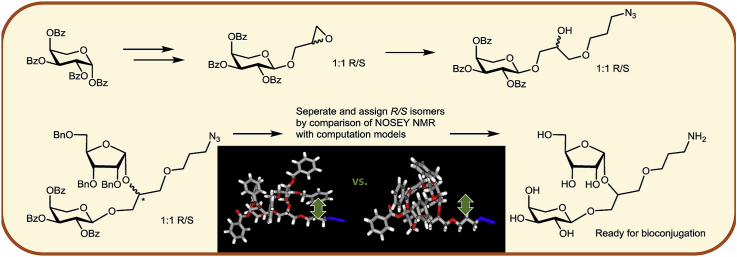

## 1 Introduction

**Fig. 1:**
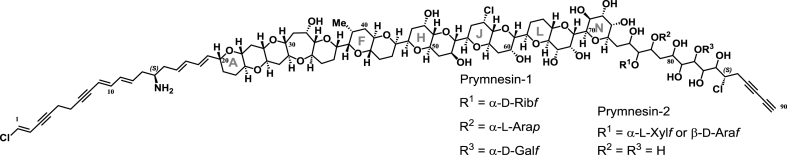
The reported structures of the A-type prymnesin ichthyotoxins [[3], [4], [5], [6]].

Prymnesins-1 and -2 are potent ichthyotoxins produced by *Prymnesium parvum*, a flagellated alga which is widely distributed across brackish waters [1]. As a result, *P. parvum* has been attributed to large scale fish kills globally, resulting in detrimental ecological and economic impacts to affected areas [2]. There are currently 16 different prymnesins reported in literature [3,4]. According to the structure of their ladder-frame polyether backbones, prymnesins can be divided into three main types. The backbone of A-type prymnesins is composed of 91 carbon atoms, B-type of 85 and C-type of 83 carbon atoms. The high diversity of these natural products arises from different patterns of glycosylation combined with the various extent of backbone chlorination. Chemical structures of three prymnesins, prymnesin-1 and prymnesin-2 derived from the A-type backbone (Fig. 1) and Prymnesin-B1, a B-type backbone derivative, have been elucidated, although the absolute configuration of several stereocenters in these molecules remains unknown [[3], [4], [5], [6]].

**Fig. 2:**
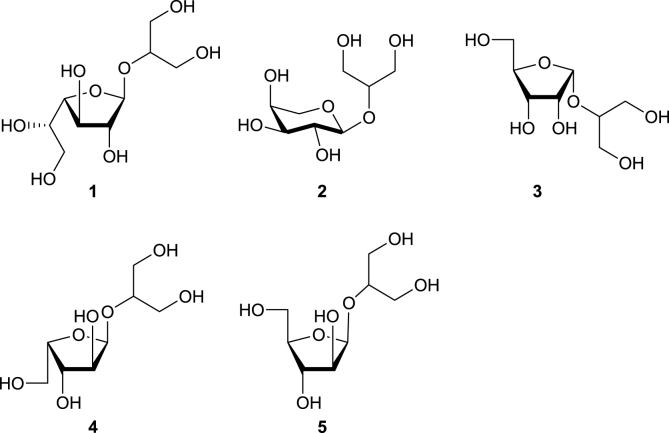
Target glyceryl glycosides as fragments of prymnesin-1 (β-ᴅ-galactofuranoside **1**, α-ʟ-arabinofuranoside **2** and α-ᴅ-ribofuranoside **3**) [3] and prymnesin-2 (either α-ʟ-xylofuranoside **4** or β-ᴅ-arabinofuranoside **5**) [3,4].

In order to gain insight into the structure and properties of prymnesin toxins, we aimed to synthesise a library of glyceryl glycosides which can serve as model fragments of specific toxins relating to prymnesin-1 and prymnesin-2 (Fig. 2). A few glyceryl glycosides have been reported in natural products literature and prepared by chemical synthesis. Thus, 1,3-dihydroxypropan-2-yl α-ᴅ-glucopyranoside is an osmolyte used by cyanobacteria to combat the effects of salt-stress and drought [7]. Glyceryl glycosides have also found use as moisturising ingredients in cosmetics [8]. ‘Floridoside’ (1,3-dihydroxypropan-2-yl α-ᴅ-galactopyranoside) has been isolated from the red alga *Mastocarpus stellatus* [9] and found to be a potent activator of the classical complement pathway [9]. Although relevant to B-type prymnesins, floridoside's chemical synthesis and characterisation has been reported in the literature [10,11], and so will not be repeated in this study. The synthesis of α-ʟ-arabinopyranoside (**2**), which is also relevant to our study, was published in 1972 but with little characterisation data.

**Fig. 3:**
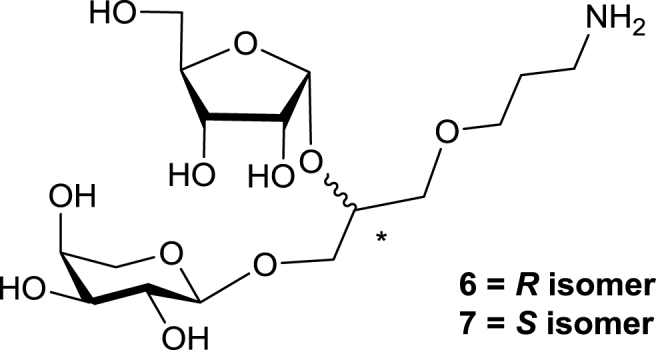
Target diastereomeric diglycosides **6** and **7**.

One of the problems associated with detection of the prymnesin toxins is that current methods rely on detection of the parent organism [12,13]. None of these methods can provide quantification of toxin levels in water samples. Furthermore, the link between amount of *P. parvum* in water and prymnesin concentration is not straight forward [1,13,14]. Thus, it has been noticed that in some cases blooms of *P. parvum* do not necessarily lead to fish kills [15]. Work in our group revealed a new mechanism of *P.* parvum toxicity, which results from mass cell lysis caused by the infection of alga with a newly discovered double stranded DNA megavirus, PpDNAV [16]. Therefore sensitive and quantitative techniques for direct detection for *P. parvum* toxins are highly desirable. As an example, antibody recognition has been developed for polyketide algal toxins such as okadaic acid and brevetoxins [17,18]. We decided to focus on a glycosylated polyol fragment of prymnesin-1 and synthesise diglycosides **6** and **7** (Fig. 3) incorporating α-ᴅ-ribofuranosyl and α-ʟ-arabinopyranosyl residues as possible candidates for antigens for anti-carbohydrate antibodies production. These fragments represent the region between C76-C78 on the prymnesin-1 backbone (Fig. 1). The design of **6** and **7** consists of glycerol as a polyol backbone mimic which is glycosylated at positions 1 and 2 and alkylated at position 3 with 3-aminopropyl linker. The linker is required for conjugation of **6** and **7** via cross-linking techniques to a protein carrier, with the aim to make carbohydrate antigens for antibody production [19,20]. As the exact stereochemistry of the glycosylated backbone region of prymnesin-1 is not yet known [3], both the (2*R*)- and (2*S*)- glycerol diastereoisomers **6** and **7** were required.

## 2 Results and discussion

### 2.1 Synthesis of 1,2-*trans*-linked glyceryl glycoside fragments of prymnesin-1

**Scheme 1:**
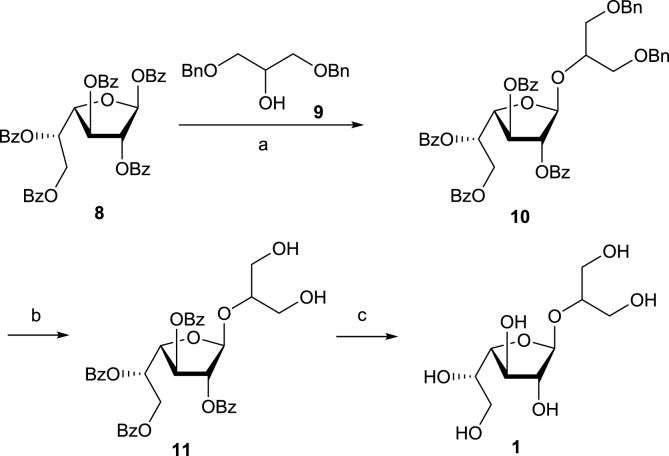
Synthesis of 1,3-dihydroxypropan-2-yl β-ᴅ-galactofuranoside (**1**). Reagents and conditions: (a) SnCl_4_, CH_2_Cl_2_, 39%; (b) H_2_, 10% Pd/C, EtOAc, Et_3_N, 64%. (c) MeOH-H_2_O-Et_3_N (5:2:1), 87%.

Tin (IV) chloride-promoted glycosylation of 1,3-di-*O*-benzyl glycerol (**9**) with per-*O*-benzoyl-β-ᴅ-galactofuranose (**8**) [21,22] produced exclusively 1,2-*trans*-glycoside **10** (Scheme 1). After hydrogenation to give **11**, the benzoyl protecting groups were removed using a 5:2:1 mixture of MeOH-H_2_O-Et_3_N leading to β-galactofuranoside **1**. The configuration of the newly formed glycosidic linkage was confirmed by the appearance of H-1′ signal either as a singlet (δ 5.61 for **10**) or a doublet (δ 4.99 with *J*_1,2_ 1.7 Hz for **1**) in the ^1^H NMR spectra. These *J*_1,2_ values are known to be characteristic for a β-galactofuranosidic linkage [22].

**Scheme 2:**
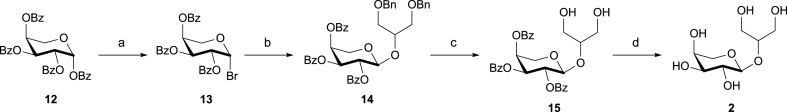
Synthesis of α-ʟ-arabinopyranoside **2**. Reagents and conditions: (a) HBr/AcOH, CH_2_Cl_2_, 95%; (b) 1,3-di-*O*-benzyl glycerol, Ag_2_CO_3_, Drierite™, toluene, 50%; (c) H_2_, 10% Pd/C, EtOAc, Et_3_N, 22%; (d) NaOMe, MeOH, 76%.

The synthesis of 1,3-dihydroxypropan-2-yl α-ʟ-arabinopyranoside (**2**) has previously been described [23], but the product was only characterised by optical rotation. Silver carbonate-promoted glycosylation of alcohol **9** with bromide **13** prepared from per-*O*-benzoyl-β-ʟ-arabinopyranose (**12**) [24,25] gave arabinopyranoside **14** in 50% yield (Scheme 2). The presence of 2-*O*-benzoyl group in donor **13** ensured the formation of only the 1,2-*trans* α-anomer **14**, and this was confirmed by NMR spectra which showed signals for the anomeric proton (doublet at 5.06 ppm with *J*_1′,2′_ 5.9 Hz) and anomeric carbon (100.4 ppm) consistent with α-configuration as followed from comparison with spectra of methyl 2,3,4-tri-*O*-benzoyl-α- and β-arabinopyranosides [26]. The glycerol benzyl ether protecting groups were removed by hydrogenation over a Pd/C catalyst, followed by NaOMe catalysed removal of the benzoates to give 1,3-dihydroxypropan-2-yl α-ʟ-arabinopyranoside (**2**) as a white powder. ^1^H NMR spectroscopy analysis of **2** showed a H-1′ signal at 4.35 ppm as a doublet with a larger *J*_1′,2′_ coupling value of 7.5 Hz, which confirmed the presence of the α-anomer.

### 2.2 Synthesis of 1,2-*cis*-linked glyceryl furanoside fragments

Synthesis of 1,2-*cis*-furanosides can prove challenging as it can be difficult to control the stereochemistry at the anomeric centre [27,28]. Unless indirect glycosylation approaches are used [[29], [30], [31]], application of non-participating protecting groups remains the main method of minimising the formation of 1,2-*trans*-glycofuranosylation products.

**Scheme 3:**
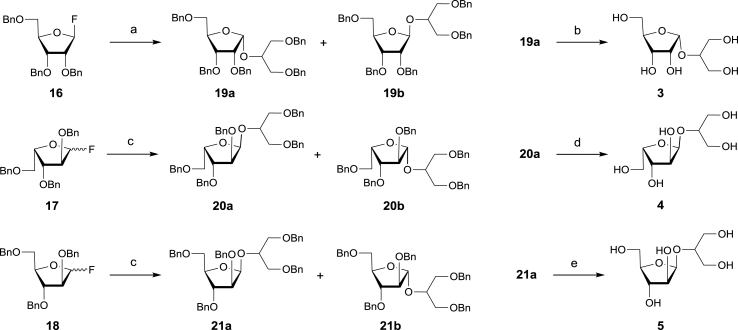
Synthesis of prymnesin-1 fragments **3**–**5**. Reagents and conditions: (a) 1,3-di-*O*-benzyl glycerol, 1 equiv. SnCl_2_, 1 equiv. TrClO_4_, MS 4 Å, Et_2_O; (b) H_2_, 10% Pd/C, MeOH-EtOH (5:1); (c) 1,3-di-*O*-benzyl glycerol, 1 equiv. SnCl_2_, 4 Å MS,Et_2_O; (d) H_2_, 10% Pd/C, MeOH-PrOH (9:1); (e) H_2_ 10% Pd/C, MeOH-EtOAc (9:1).

For syntheses of 1,2-*cis*-furanosides **3**–**5**, we resorted to Mukaiyama's general methodology employing SnCl_2_-promoted glycosylation with benzylated glycofuranosyl fluorides [32,33]. 2,3,5-Tri-*O*-benzylated glycofuranosyl fluorides **16**–**18** were synthesised in high yield by fluorination of the corresponding hemiacetals with DAST (for more details see Suppl. Information) [34,35]. For the glycosylation of 1,3-di-*O*-benzyl-glycerol (**9**) with β-d-ribofuranosyl fluoride **16**, only the purified β-fluoride was used. As this resulted in a mixture of α- and β-linked products (**19a** & **19b**), we decided that there was no benefit in removing the 1,2-*cis* fluoride. Therefore, in the case of ʟ-xylofuranosyl and ʟ-arabinofuranosyl glycosides **17** and **18**, respectively, α/β-mixtures mixtures of the fluoride anomers were employed (Scheme 3).

All glycosylations, which were promoted by one equivalent of SnCl_2_ and carried out in the presence of 4 Å MS in diethyl ether, resulted in the formation of anomeric mixtures of furanosides **19–21** in which the 1,2-*cis*-glycosides dominated. It has been previously noted that in Mukaiyama's synthesis of ribofuranosides both yields and stereoselectivity could be improved with the addition of triphenylmethyl perchlorate (TrClO_4_) [32]. In our hands, addition of TrClO_4_ to the reaction involving donor **16** had only a negligible effect on the reaction outcome, so this variation was not applied in glycosylation with **17** and **18**.

To establish the anomeric configuration of each component of mixtures of glycosylation products **19–21**, individual glycosides were isolated and characterised by NMR spectroscopy. It is well known that α- and-β-glycofuranosides can be distinguished on the basis of the differences in values of their ^3^*J*_H-1,H-2_ coupling constants in ^1^H NMR spectra, which are in the range of 0–2 Hz for of 1,2-*trans*-furanosides and **∼**4 Hz larger for 1,2-*cis*-furanosides [[36], [37], [38]]. In addition, resonances of anomeric carbons of 1,2-*cis*-furanosides in ^13^C NMR spectra appear 4–6 ppm upfield compared to corresponding signals of 1,2-*trans*-furanosides [37]. Thus, the stereochemistry of the glycosidic linkages in pairs of anomeric glycosides **19–21** was determined using these characteristic ^1^H and ^13^C NMR signals, as summarised in Table 1. Finally, per-*O*-benzylated 1,2-*cis*-furanosides structurally related to A-type prymnesin were deprotected by catalytic hydrogenolysis over Pd/C (Scheme 3). NMR analyses of resulting α-ᴅ-ribofuranoside **3**, α-l-xylofuranoside **4** and β-ᴅ-arabinofuranoside **5** confirmed their anomeric configuration (Table 1).

**Table 1:** Characteristic data for anomeric signals in ^1^H and ^13^C NMR spectra of pentofuranosides **3–5, 19–21** and **27–28**.

Compound	Configuration	δ_H-1_ (ppm)	*J*_1,2_ (Hz)	δ_C-1_ (ppm)
**3**	1,2-*cis* (α-d-*ribo*)	5.11	4.3	102.0
**4**	1,2-*cis* (α-l-*xylo*)	5.09	4.4	101.3
**5**	1,2-*cis* (β-d-*arabino*)	5.05	4.7	101.3
**19a**	1,2-*cis* (α-d-*ribo*)	5.38	4.3	101.5
**19b**	1,2-*trans* (β-d-*ribo*)	5.30	0	104.9
**20a**	1,2-*cis* (α-l-*xylo*)	5.35	4.3	99.9
**20b**	1,2-*trans* (β-l-*xylo*)	5.31	1.9	107.0
**21a**	1,2-*cis* (β-d-*arabino*)	5.32	4.4	100.8
**21b**	1,2-*trans* (α-d-*arabino*)	5.35	<1	106.1
**27**	1,2-*cis* (α-d-*ribo*)	5.21	4.2	101.5
**28**	1,2-*cis* (α-d-*ribo*)	5.23	4.4	101.4

### 2.3 Synthesis of diastereomeric diglycoside **6** and **7**

**Scheme 4:**
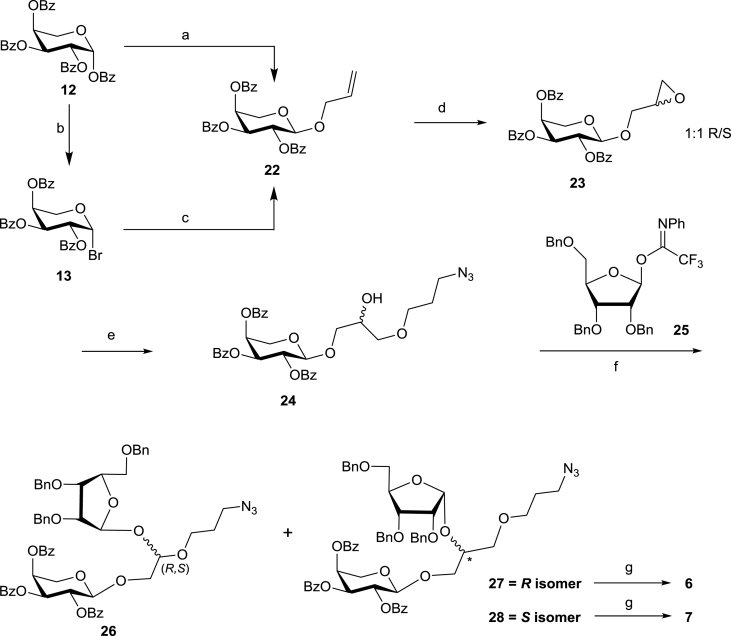
Synthesis of diastereomeric diglycosides **6** and **7**. Reagents and conditions: (a) AllOH, BF_3_-OEt_2_, CH_2_Cl_2_, α/β 3:1; (b) HBr/AcOH, CH_2_Cl_2_, 85%; (c) AllOH, Ag_2_CO_3_, DCE, 4 Å MS, 62%; (d) mCPBA, DCE, reflux, 56%; (e) 3-azidopropanol, Sc(OTf)_3_, toluene, 40%; (f) TMSOTf, CH_2_Cl_2_, 4 Å MS, −78 °C, 15% (**27**), 10% (**28**); (g) i) H_2_, 10% Pd/C, EtOAc-MeOH 1:1. ii) MeOH/H_2_O/Et_3_N (5:2:1), 59% (**6**), 41% (**7**).

We initially tried to synthesise the separate enantiomers of **23** by DMTST promoted glycosylation of optically pure glycidol with ethyl 2,3,4-tri-*O*-benzoyl-1-thio-β-l-arabinopyranoside [39]. Unfortunately the glycosylation was very low yielding and gave a complex mixture of products which proved challenging to separate. As such we changed our approach and for the synthesis of 1,2-*O*-glycosylated glycerol derivatives **6** and **7** we used allyl alcohol as a precursor. It was first arabinosylated, then oxidised to a diastereomeric mixture of epoxides which were then regioselectively ring-opened with 3-azidopropanol in the presence of Sc(OTf)_3_ (Scheme 4). This generated an acceptor for a final ribosylation step and introduced a functionalised linker at the same time (Scheme 4). Attempts to glycosylate allyl alcohol with per-*O*-benzoyl β-ʟ-arabinopyranose (**12**) in the presence of BF_3_.OEt_2_ gave a 3:1 mixture of α and β anomers, which proved very challenging to separate, in a combined 76% yield. To ensure that only the desired α-anomer was synthesised, Koenigs-Knorr glycosylation conditions with perbenzoylated ʟ-arabinopyranosyl bromide **13** were applied, leading to the target 1,2-*trans*-ʟ-arabinopyranoside **22** in 62% yield.

The 1,2-*trans* configuration of **22** was confirmed by ^1^H NMR spectrum which showed the H-1 signal at 4.82 ppm as a doublet with a *J*_1′,2′_ coupling value of 6.1 Hz. Epoxidation of the alkene **22** was achieved using mCPBA [8] giving compound **23** as an inseparable 1:1 mixture of *(R*,*S)-*diastereomers, as judged by integration of the anomeric proton signals at 4.92 and 4.82 ppm. Using a combination of HSQCed and COSY 2D NMR spectra, it was possible to fully assign the ^1^H and ^13^C NMR spectra of the individual isomers. Since both (*R*)- and (*S*)-isomers of the prymnesin-1 fragments were targeted, the synthesis continued without diastereomer separation at this stage.

**Fig. 4:**
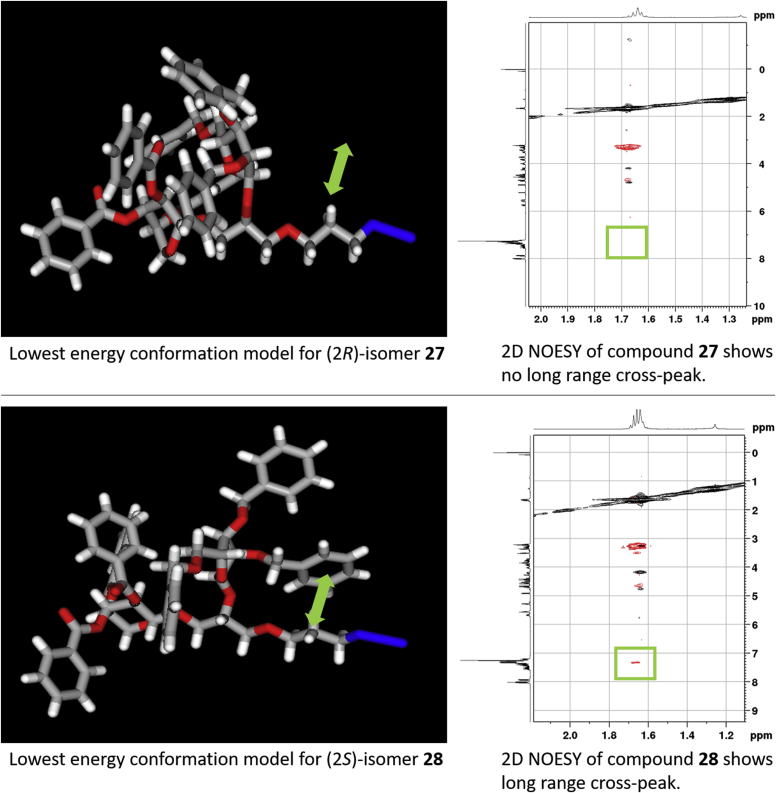
3D Model representations of lowest energy calculations (MarvinSketch 15.1.19.0 Conformer Calculator Plugins) for diastereomers **27** and **28** and expanded 2D NOESY NMR spectra.

The epoxide **23** was ring-opened with 3-azidopropanol in the presence of Sc(OTf)_3_, leading to installation of a linker with azido functional group, suitable for later reduction and conjugation of the glycoside fragment to a carrier protein for antibody generation. The resulting alcohol **24** remained an 1:1 mixture of (*R*,*S*)-diastereomers which were inseparable, but again it was possible to fully assign their ^1^H and ^13^C NMR spectra using a combination of HSQCed and COSY 2D NMR. To effect 1,2-*cis*-ribofuranosylation of **24,** we employed imidate **25** which has been used several times to good effect for stereoselective ribosylations using TMSOTf as a promotor and either DCM or DCE as a solvent [[40], [41], [42]]. The initial attempt to couple **24** to **25** was carried out in DCE at −30 °C and resulted in predominant formation of the β-riboside (α/β 1.0:5.8, as judged by ^1^H NMR). The stereoselectivity improved when the glycosylation was conducted in CH_2_Cl_2_ at −78 °C. Since acceptor **24** was a 1:1 (*R*,*S*)-mixture, the glycosylation resulted in four products. ^1^H NMR of the mixture of ribosylation products showed a slight excess of the desired α-ribosides, with an α/β ratio of 1.4:1. The target α-ribosides **27** and **28** were separated from the (*R*,*S*)-mixture of β-ribosides **26** using preparative TLC. The stereochemistry of the newly formed 1,2-*cis*-ribofuranosyl linkages in **27** and **28** was confirmed by ^1^H and ^13^C NMR, which showed typical values for 1,2-*cis*-furanosides (Table 1). To assign (*R*,*S*)-configurations in **27** and **28**, we used a combination of NMR spectroscopy and modelling approaches. The lowest energy conformations for both isomers were calculated using MarvinSketch 15.1.19.0 Conformer Calculator Plugin (Drieding Force Field), and revealed a noticeable difference in the orientation of the benzyl protecting groups with respect to the azido-propyl linker (Fig. 4). In the computational model for the (*S*)-isomer **28**, the phenyl ring of the 2-OBn group of the ribosyl residue is in close proximity to the azidopropyl group. In contrast, the model of (*R*)- isomer **27** showed the aromatic benzyl protecting groups clustered together and oriented away from the azidopropyl group. The conclusions arising from the computational models were reinforced by 2D NOESY NMR spectra of compound **28**, which show a clear cross-peak between signals for the azidopropyl and the benzyl protecting groups. In contrast, no such cross-peaks can be identified in NOESY spectra of diastereoisomer **27**. These observations allowed us to conclude that the chiral centres of the aglycones in compounds **27** and **28** have (*R*)- and (*S*)-configurations, respectively.

Finally, global deprotection of compounds **27** and **28** was achieved by first Pd/C-catalysed hydrogenolysis of benzyl groups with simultaneous reduction of the azide to amine, followed by de-*O*-benzoylation using a mixture of MeOH-H_2_O-Et_3_N (5:2:1). In this way, diastereomeric 2,3-di-*O*-glycosylated derivatives of glycerol, **6** and **7**, equipped with an aminopropyl linker were synthesised in 59% and 41% yields, respectively.

## 3 Conclusions

A library of glyceryl glycoside fragments inspired by the structures of prymnesin toxins was prepared. Central to these syntheses were efforts to control glycosylation stereochemistry. The 1,2-*trans*-linked fragment 1,3-dihydroxypropan-2-yl β-ᴅ-galactofuranoside (**1**) was synthesised using SnCl_4_ promoted glycosylation with excellent stereocontrol, while 1,3-dihydroxypropan-2-yl α-ʟ-arabinopyranoside (**2**) was synthesised using Koenigs-Knorr methodology, again with excellent stereocontrol at the anomeric position [43]. 1,2-*cis*-Glyceryl glycoside fragments **3**, **4** and **5** were successfully synthesised from the corresponding glycofuranosyl fluoride donors, but with only moderate stereoselectivity.

Elaborating with respect to the stripped-down glyceryl glycoside fragments, two glyceryl diglycoside fragments inspired by prymnesin-1, **6** and **7**, were also chemically synthesised. As the stereochemistry of the prymnesin backbone in this region has not been established, both the 2*R*- and 2*S*- diastereoisomers with respect to the glycerol backbone were synthesised. This was achieved by glycosylating 3-(3-azidopropoxy)-2-*R/S-*hydroxypropyl 2,3,4-tri-*O*-benzoyl-α-ʟ-arabinopyranoside (**24**) with 2′,3′,5′-tri-*O*-benzyl-β-ᴅ-ribofuranosyl (*N*-phenyl)-2,2,2-trifluoroacetimidate (**25**), which gave a mixture of α/β-ribosides in a ratio of 1.4:1. The α-ribosides were separable both from the β-ribosides (**26**) and also from each other, to yield the diastereomeric (2*R*)- and (2*S*)-3-(3-azidopropoxy)-2-[(2′,3′,5′-tri-*O*-benzyl-α-ᴅ-ribofuranosyl)oxy]propyl-2″,3″,4″-tri-*O*-benzoyl-α-ʟ-arabinopyranoside (**27** and **28** respectively). The assignment of the stereochemistry at the 2° position of the glycerol backbone was achieved by comparing nOe NMR spectra with computational models of the lowest energy conformations of both isomers. Global deprotection of **27** and **28** yielded the desired (2*R*)-3-(3-aminopropoxy)-2-(α-ᴅ-ribofuranosyloxy)propyl α-ʟ-arabinopyranoside (**6**) and (2*S*)-3-(3-aminopropoxy)-2-(α-ᴅ-ribofuranosyloxy)propyl α-ʟ-arabinopyranoside (**7**), respectively, which are suitably functionalised for prospective bioconjugation studies.

## 4 Experimental

### 4.1 General

Reagents and anhydrous solvents were supplied by Sigma Aldrich, and were used without further purification. Analytical grade solvents were supplied by Fischer Scientific. Protected sugars which were not synthesised in the lab were supplied by Carbosynth. Glassware was oven-dried and purged with nitrogen immediately before use, and reactions requiring inert atmosphere were run under N_2_.

Reactions were monitored by thin-layer chromatography (TLC) on aluminium-backed, pre-coated silica gel plates (Silica Gel 60 F254, Merk) with the indicated eluents, and the TLC plates were visualised under UV light (*λ* 254 nm) and charring by dipping in ethanol-sulfuric acid (95:5, v/v) followed by heating. Preparative TLC was run on Analtech preparative uniplates (silica gel 1000 μm, 20 × 20 cm) and flash column chromatography (FCC) was performed on a Biotage Horizon Isolera One using pre-packed SNAP ULTRA 25 μm silica gel cartridges.

**Fig. 5:**
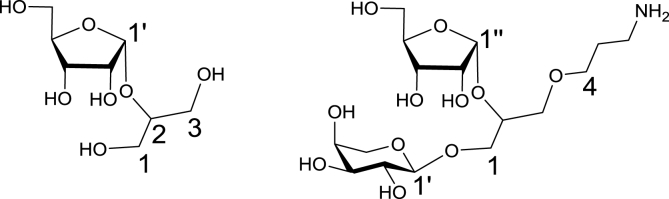
Numbering system used for assigning NMR spectra.

NMR spectra were recorded using a Bruker Ultrashield Plus 400 MHz spectrometer at 298 K and analysed using TopSpin software. Chemical shifts (δ) are reported in ppm with respect to internal tetramethylsilane or the residual HOD signal in D_2_O. NMR assignments were made with the aid of COSY and HSQCed experiments, with signals of glycosyl residues being distinguished from those of aglycon by numbers with prime and double prime as shown in Fig. 5. Note that compounds **23** and **24** are a mixture of *R*- and *S*-isomers; whilst we could not determine which isomer was *R*- and which was *S*-, we could determine which molecule the signals related to via 2D NMR. Therefore, all signals marked with an asterisk are for one diastereoisomer; those without an asterisk are the other.

Optical rotation values were measured using a Perkin Elmer^®^ Model 341 Polarimeter at 20 °C at a wavelength of 589 nm unless otherwise noted. Infrared spectra were recorded using a Perkin Elmer^®^ Spectrum BX and UV–vis spectra using a Varian 50 Bio spectrometer. Low resolution mass spectrometry was employed for monitoring reactions using an Advion Expression CMS spectrometer by direct injection or extraction from a TLC plate using an Advion Plate Express, with a 9:1 mixture of methanol-aqueous formic acid (0.1%) used as a mobile phase. For high resolution mass spectrometry (HRMS) 0.1% (w/v) samples were prepared by dissolving or diluting in methanol-formic acid (1:1) and infused into a Synapt G2-Si mass spectrometer (Waters, Manchester, UK) at 5–10 L min^−1^ using a Harvard Apparatus syringe pump. The mass spectrometer was controlled by MassLynx 4.1 software (Waters). It was operated in high resolution and positive ion mode and calibrated using sodium formate. The sample was analysed for 2 min with 1 s MS scan time over the range of m/z 50–1200 with 3.5 kV capillary voltage, 40 V cone voltage, 120 °C cone temperature. Leu-enkephalin peptide (1 ng μL^−1^, Waters) was infused at 10 L min^−1^ as a lock mass (m/z 556.2766) and measured every 10 s. Spectra were generated in MassLynx 4.1 by combining a number of scans, and peaks were centred using automatic peak detection with lock mass correction. For completeness, synthetic procedures to produce all known compounds used in this study are included as electronic Supplementary Information.

### 4.2 1,3-Bis(benzyloxy)propan-2-yl 2,3,5,6-tetra-*O*-benzoyl-β-ᴅ-galactofuranoside (**10**)

A solution of per-*O*-benzoyl-β-ᴅ-galactofuranose (**8**) [44] (250 mg, 0.36 mmol) dissolved in CH_2_Cl_2_ (4 mL) was cooled in an ice bath. SnCl_4_ solution (1 M in CH_2_Cl_2_, 400 μL, 0.4 mmol) was slowly added by syringe. After stirring for 15 min at 0 °C, 1,3- di-*O*-benzyl-glycerol (**9**) (80 μL, 0.32 mmol) was added by syringe and the mixture was allowed to warm up to room temperature and was stirred for 18 h after which TLC analysis (hexane-EtOAc 7:3) showed complete consumption of **8**. The mixture was diluted with CH_2_Cl_2_ (30 mL) and washed with sat. aqueous NaHCO_3_ (2 × 10 mL). The organic layers were combined, dried over MgSO_4_, filtered and dried *in vacuo* to give a crude residue which was purified by FCC to **10** (120 mg, 39%) as a colourless oil; R_f_ 0.61 (hexane/EtOAc 7:3); [α]_D_ −1.2 (*c* 1.0, CHCl_3_); ^1^H NMR (400 MHz, CDCl_3_) 8.07–8.04 (m, 2H, Ar), 7.97–7.95 (m, 1H, Ar), 7.90–7.88 (m, 1H, Ar), 7.57–7.20 (m, 25H, Ar), 6.03–5.99 (m, 1H, H-5′), 5.61 (s, 1H, H-1′), 5.59 (d, *J*_3′,4′_ = 5.4 Hz, 1H, H-3′), 5.55 (s, 1H, H-2′), 4.75 (dd, *J*_3′,4*′*_ = 5.4 Hz, *J*_*4′,5′*_ = 3.4 Hz, 1H, H-4′), 4.73 (dd, *J*_5′,6′a_ = 7.7 Hz, ^2^*J*_6′a,6′b_ = 12.0 Hz, 1H, H-6′a), 4.58–4.52 (m, 3H, H-6′b, PhC*H*_2_), 4.51 (d, ^2^*J* = 11.9 Hz, PhC*H*H), 4.45 (d, ^2^*J* = 11.9 Hz, PhC*H*H), 4.25–4.20 (m, 1H, H-2), 3.71–3.67 (m, 2H, H-1), 3,62 (d, *J*_*2.3*_ = 5.3 Hz, 2H, H-3); ^13^C NMR (100.6 MHz, CDCl_3_): 166.1, 165.8, 165.7, 165.4 (4 × C=O), 138.2, 138.1, 133.4, 133.3, 133.2, 133.0, 130.0, 129.9, 129.8, 129.7, 129.6, 129.2, 129.1, 128.4, 128.4, 128.3, 127.7, 127.6 (Ar), 105.0 (C1′), 82.1 (C2′), 81.4 (C4′), 77.8 (C3′), 74.6 (C2), 73.5, 73.5 (2 × Ph*C*H_2_), 70.6 (C1), 70.3 (C5′), 70.2 (C3), 63.9 (C6′); HRMS (ESI^+^) m/z calc. for C_51_H_46_O_12_Na^+^ 873.2887 ([M+Na]^+^) found 873.2875 [M+Na]^+^.

### 4.3 1,3-Dihydroxypropan-2-yl 2,3,5,6-tetra-*O*-benzoyl-β-ᴅ-galactofuranoside (**11**)

To a solution of 1,3-bis(benzyloxy)propan-2-yl 2,3,5,6-tetra-*O*-benzoyl-β-ᴅ-galactofuranoside (**10**) (115 mg, 140 μmol) in EtOAc/EtOH (10:1) (20 mL) was added activated 10% palladium on charcoal (10 mg). The system was flushed with N_2_ ( × 3) followed by H_2_ ( × 3) and stirred overnight at room temperature. After the system had been flushed with N_2_ ( × 3) the catalyst was filtered off and the solvent removed under reduced pressure to give **11** (60 mg, 64%) as a colourless oil; R_f_ 0.18 (hexane-EtOAc 1:1); [α]_D_ = −1.1 (*c* 1.0, CHCl_3_); ^1^H NMR (400 MHz, CDCl_3_): 8.09–8.06 (m, 2H, Ar), 8.02–7.97 (m, 4H, Ar), 7.93–7.90 (m, 2H, Ar), 7.59–7.51 (m 4H, Ar), 7.49–7.30 (m, 8H, Ar), 6.00–5.96 (m 1H, H-5′), 5.73 (dd, *J*_2′,3′_ = 2.1 Hz, *J*_3′,4′_ = 5.7 Hz, 1H, H-3′), 5.52 (s, 1H, H-1′), 5.49 (d, *J*_2′,3′_ = 2.1 Hz, 1H, H-2′), 4.85 (dd, *J*_3′,4′_ = 5.7 Hz, *J*_4′,5′_ = 3.8 Hz, 1H, H-4′), 4.79 (dd, *J*_5′,6′a_ = 4.6 Hz, ^2^*J*_6′a,6′b_ = 11.8 Hz, 1H, H-6′a), 4.72 (dd, *J*_5′,6′b_ = 6.6 Hz, ^2^*J*_6′a,6′b_ = 11.8 Hz, 1H, H-6′b), 3.94–3.89 (m, 1H, H-2), 3.78–3.72 (m 4H, H-2,3), 2.56 (bs, 1H, OH), 2.47 (bs, 1H, OH); ^13^C NMR (100.6 MHz, CDCl_3_): 166.2, 166.2, 165.7, 165.6 (4 × C=O), 133.7, 133.7, 133.4, 133.2, 130.0, 129.9, 129.8, 129.5, 129.3, 128.8, 128.6, 128.5, 128.5, 128.4 (Ar), 106.5 (C1′), 83.3 (C2′), 81.1 (C4′), 80.8 (C2), 77.3 (C3′), 70.3 (C5′), 63.1 (C1), 63.1 (C6′), 62.4 (C3); HRMS (ESI^+^) m/z calc. for C_37_H_34_O_12_Na^+^ 693.1948 ([M+Na]^+^) found 693.1956 [M+Na]^+^.

### 4.4 1,3-Dihydroxypropan-2-yl β-ᴅ-galactofuranoside (**1**)

1,3-Dihydroxypropan-2-yl 2,3,5,6-tetra-*O*-benzoyl-β-ᴅ-galactofuranoside (**11**) (55 mg, 82 μmol) was dissolved into a solution of MeOH/H_2_O/NEt_3_ (5:2:1, 8 mL) and stirred vigorously for 18 h at room temperature. The solvent was removed *in vacuo*, the crude mixture was dissolved in MeOH (5 mL) and passed through Dowex^®^ 1X2-400 (OH^−^) ion exchange resin (1 g). The eluted solution was concentrated and the residue was dried *in vacuo* to give **1** (18 mg, 87%) as a colourless oil; R_f_ 0.06 (toluene/MeOH, 8:2); [α]_D_ −144 (c 0.5, MeOH); ^1^H NMR (400 MHz, CD_3_OD): 4.99 (d, *J*_1′,2′_ = 1.7 Hz, 1H, H-1′), 3.93–3.92 (m, 2H, H-3′,4′), 3.90 (dd, *J*_1′,2′_ = 1.7 Hz, *J*_2′,3′_ = 4.0 Hz, 1H, H-2′), 3.65–3.58 (m, 2H, H-5′,2), 3.56–3.48 (6H, H-1,3,6′a,6′b); ^13^C NMR (100.6 MHz, CD_3_OD): 107.8 (C1′), 83.4 (C3′), 81.6 (C2′), 78.8 (C2), 77.3 (C4′), 71.0 (C5′), 63.0 (C6′), 61.9 (C1), 61.1 (C3); HRMS (ESI^+^) calc. for C_9_H_18_O_8_Na^+^ 277.0899 ([M+Na]^+^) found 277.0895 [M+Na]^+^.

### 4.5 1,3-Bis(benzyloxy)propan-2-yl 2,3,4-tri-*O*-benzoyl-α-ʟ-arabinopyranoside (**14**)

2,3,4-Tri-*O*-benzoyl-β-ʟ-arabinopyranosyl bromide (**13**) [25] (2.1 g, 4.0 mmol) was dried azeotropically with dry toluene (3 × 20 mL), dissolved into dry toluene (10 mL) under N_2_ and then 1,3-*O*-di-benzyl-glycerol (**9**) (0.9 mL, 3.8 mmol) and Ag_2_CO_3_ (1.8 g, 6.4 mmol) were added. The mixture was stirred at 55 °C under N_2_ for 4 h after which time TLC (hexane/EtOAc 3:1) showed complete consumption of the glycosyl bromide. The reaction mixture was filtered and the filtrate was concentrated under reduced pressure. Purification by FCC gave **14** (1.5 g, 50%) as a colourless oil, R_f_ 0.48 (toluene/EtOAc 9:1); [α]_D_ +94.4 (*c* 1.0, CHCl_3_); ^1^H NMR (400 MHz, CDCl_3_): 8.05–7.94 (m, 5H, Ar), 7.58–7.17 (m, 20H, Ar), 5.71 (dd, *J*_1′,2′_ = 5.9 Hz, *J*_2′,3′_ = 8.3 Hz, 1H, H-2′), 5.67–5.65 (m, 1H, H-4′), 5.60 (dd, *J*_2′,3′_ = 8.3 Hz, *J*_3′,4′_ = 3.5 Hz, 1H, H-3′), 5.06 (d, *J*_1′,2′_ = 5.9 Hz, 1H, H-1′), 4.35 (s, 2H, PhC*H*_2_), 4.37–4.32 (m, 3H, H-5a′, PhC*H*_2_), 4.15–4.12 (m, 1H, H-2), 3.82 (dd, *J*_4′,5′_ = 2.3 Hz, ^2^*J*_5a’,5b’_ = 12.6 Hz, 1H, H-5b′), 3.69 (dd, *J*_1a,2_ = 5.3 Hz, ^2^*J*_1a,1b_ = 10.3 Hz, 1H, H-1a), 3.63–3.59 (m, 2H, H-1b,3a), 3.50 (dd, *J*_2,3b_ = 6.4 Hz, *J*_3a,3b_ = 10.3 Hz, 1H, H-3b); ^13^C NMR (100.6 MHz, CDCl_3_): 165.7, 165.6, 165.2 (3 × C=O), 138.2, 138.1, 133.4, 133.3, 129.9, 129.8, 129.5, 129.2, 128.5, 128.4, 128.3, 127.7, 127.6, 127.5, 127.5 (Ar), 100.4 (C1′), 77.6 (C2), 73.4, 73.4 (2 × Ph*C*H_2_), 70.8 (C1), 70.4 (C3′), 70.4 (C3), 70.2 (C2′), 68.3 (C4′), 62.0 (C5′); HRMS (ESI^+^) m/z calc. for C_43_H_40_O_10_Na^+^ 739.2519 [M+Na]^+^ found 739.2513 [M+Na]^+^.

### 4.6 1,3-Dihydroxypropan-2-yl 2,3,4-tri-*O*-benzoyl-α-ʟ-arabinopyranoside (**15**)

To a solution of 1,3-bis(benzyloxy)propan-2-yl 2,3,4-tri-*O*-benzoyl-α-ʟ-arabinopyranoside (**14**) (1.50 g, 2.1 mmol) in EtOAc/EtOH 10:1 (50 mL) was added palladium on activated charcoal (10% Pd basis) (50 mg). The system was flushed with N_2_ ( × 3) followed by H_2_ ( × 3) and stirred overnight at room temperature. After the system had been flushed with N_2_ ( × 3) the catalyst was filtered off and the solvent removed under reduced pressure to give **15** (182 mg, 22%) as a white powder. R_f_ 0.21 (EtOAc/hexane 6:4); [α]_D_ +261 (*c* 1.0, CHCl_3_); ^1^H NMR (400 MHz, CDCl_3_): 8.10–8.08 (m, 2H, Ar), 8.01–7.99 (m, 2H, Ar), 7.89–7.87 (m, 2H, Ar), 7.62–7.27 (m, 9H, Ar), 5.80 (dd, *J*_1′,2′_ = 7.3 Hz, *J*_2′,3′_ = 9.7 Hz, 1H, H-2′), 5.73–5.71 (m, 1H, H-4′), 5.62 (dd, *J*_2′,3′_ = 9.7 Hz, *J*_3′,4′_ = 3.4 Hz, 1H, H-3′), 4.90 (d, *J*_1′,2′_ = 7.3 Hz, 1H, H-1′), 4.36 (dd, *J*_4′,5′a_ = 2.8 Hz, ^2^*J*_5′a,5′b_ = 13.2 Hz, 1H, H-5′a), 3.96 (dd, *J*_4′,5′b_ = 1.5 Hz, ^2^*J*_5′a,5′b_ = 13.2 Hz, 1H, H-5′b), 3.86–3.83 (m, 1H, H-2), 3.69–3.67 (m, 2H, H-1), 3.58–3.55 (m, 2H, H-3), 2.85 (bs, 1H, OH),2.05 (bs, 1H, OH); ^13^C NMR (100.6 MHz, CDCl_3_): 165.7, 165.6, 165.6 (3 × C=O), 133.7, 133.6, 133.5, 129.9, 129.8, 129.7, 129.3, 129.0, 128.9, 128.6, 128.6, 128.4, (12 × Ar), 102.0 (C1′), 83.9 (C2), 71.0 (C3′), 70.4 (C2′), 68.7 (C4′), 64.0 (C5′), 62.7 (C1), 62.4 (C3); HRMS (ESI)^+^ calc. for C_29_H_28_O_10_Na^+^ 559.1580 [M+Na]^+^ found 559.1577 [M+Na]^+^.

### 4.7 1,3-Dihydroxypropan-2-yl α-ʟ-arabinopyranoside (**2**)

Sodium (20 mg) was added to dry methanol (18 mL) under N_2_. After the cessation of effervescence 1,3-dihydroxypropan-2-yl 2,3,4-tri-*O*-benzoyl-α-ʟ-arabinopyranoside (**15**) (180 mg, 0.3 mmol) was added in dry MeOH (8 mL) and the mixture was stirred overnight at room temperature. The reaction mixture was neutralised using Amberlite IR-120 (H^+^) resin, filtered and the solvent removed *in vacuo*. The crude mixture was dissolved in H_2_O and passed through Dowex^®^ 1X2-400 (OH^−^ form) resin (1 g) to give **2** (62 mg, 76%) as a colourless oil. [α]_D_ + 6.8 (*c* 1.0, H_2_O) [Lit: [α]_D_^27^ + 5 (H_2_O)]; ^1^H NMR (400 MHz, D_2_O): 4.35 (d, *J*_1′,2′_ = 7.5 Hz, 1H, H-1′), 3.83–3.78 (m, 2H, H-4′,5′a), 3.77–3.73 (m, 1H, H-2), 3.65–3.62 (m, 6H, H-1,2,5′b,3′), 3.45 (dd, *J*_1′,2′_ = 7.5 Hz, *J*_1′,2′_ = 9.4 Hz, 1H, H-2′); ^13^C NMR (100.6 MHz, D_2_O): 103.4 (C1′), 81.1 (C2), 72.9 (C3′), 71.3 (C2′), 68.4 (C4′), 66.2 (C5′), 61.4 (C1), 61.0 (C3); HRMS (ESI^+^) m/z calc. for C_8_H_16_O_7_Na^+^ 247.0794 [M+Na]^+^ found 247.0798 [M+Na]^+^.

### 4.8 1,3-Bis(benzyloxy)propan-2-yl 2,3,5-tri-*O*-benzyl-α-ᴅ-ribofuranoside (**19a**) and 1,3-bis(benzyloxy)propan-2-yl 2,3,5-tri-*O*-benzyl-β-ᴅ-ribofuranoside (**19b**)

2,3,5-Tri-O-benzyl-β-ᴅ-ribofuranosyl fluoride (**16**) [35] (210 mg, 0.5 mmol), SnCl_2_ (95 mg, 0.5 mmol) and triphenylmethyl perchlorate (170 mg, 0.5 mmol) were dissolved into dry Et_2_O (5 mL) containing 4 Å MS (1.0 g) under N_2_. The reaction vessel was wrapped with aluminium foil to exclude light, cooled to −15 °C. 1,3-Di-*O*-benzyl glycerol (**9**) (100 μL, 0.40 mmol) was added in a single portion and the reaction mixture was stirred at −15 °C for 6 h before kept at 4 °C overnight. The reaction mixture was diluted with Et_2_O (50 mL), filtered, and the filtrate washed with saturated aqueous NH_4_Cl solution (3 × 10 mL). The organic layer was separated, dried over MgSO_4_ and filtered before the solvent was removed under reduced pressure. The anomers were separated by FCC to give **19a** and **19b** as colourless oils.

The α-anomer **19a**: R_f_ 0.10 (hexane/Et_2_O 7:3); [α]_D_ +52.5 (*c* 1.0, CHCl_3_); ^1^H NMR (400 MHz, CDCl_3_): 7.32–7.18 (m, 25H, Ar), 5.38 (d, *J*_1′,2′_ = 4.3 Hz, 1H, H-1′), 4.73–4.38 (m, 10H, 5 × PhC*H*_2_), 4.26–4.24 (m, 1H, H-4′), 4.23–4.17 (m, 1H, H-2), 3.82 (dd, *J*_2′,3′_ = 7.1 Hz, *J*_3′,4′_ = 3.9 Hz, 1H, H-3′), 3.75 (dd, *J*_1′,2′_ = 4.3 Hz, *J*_2′,3′_ = 7.1 Hz, 1H, H-2′), 3.74–3.59 (m, 4H, H-1,3), 3.41 (dd, *J*_4′,5′a_ = 3.9 Hz, ^2^*J*_5′a,5′b_ = 10.6 Hz, 1H, H-5′a), 3.34 (dd, *J*_4′,5′b_ = 4.2 Hz, ^2^*J*_5′a,5′b_ = 10.6 Hz, 1H, H-5′b); ^13^C NMR (100.6 MHz, CDCl_3_): 138.6, 138.6, 138.4, 138.1, 138.0 (5 × Ar), 128.3, 128.3, 128.2, 128.0, 127.9, 127.6, 127.6, 127.6, 127.5, 127.5, 127.4 (Ar), 101.5 (C1′), 81.5 (C4′), 77.2 (C2′), 75.6 (C2), 75.5 (C3′), 73.4, 73.4, 73.3 72.2, 72.0 (5 × *C*H_2_Ph), 71.7 (C1), 70.7 (C3), 69.9 (C5′); HRMS (ESI^+^) m/z calc. for C_43_H_46_O_7_Na^+^ 697.3141 [M+Na]^+^ found 697.3129 [M+Na]^+^.

The β-anomer **19b**: R_f_ 0.20 (hexane/Et_2_O 7:3); [α]_D_ + 42.4 (*c* 1.0, CHCl_3_); ^1^H NMR (400 MHz, CDCl_3_): 7.31–7.22 (m, 25H, Ar), 5.30 (s, 1H, H-1′), 4.65–4.39 (m, 10H, PhC*H*_2_), 4.36–4.30 (m, 1H, H-4′), 4.05–4.01 (m, 1H, H-2), 4.03 (dd, *J*_2′,3′_ = 4.8 Hz, *J*_3′,4′_ = 7.4 Hz, 1H, H-3′), 3.92 (d, *J*_2′,3*′*_ = 4.8 Hz, 1H, H-2′), 3.65–3.42 (m, 6H, H-1,3,5′a,5′b); ^13^C NMR (100.6 MHz, CDCl_3_): 138.4, 138.3, 138.3, 138.0, 138.0, 128.4, 128.4, 128.3, 128.0, 127.8, 127.7, 127.6, 127.6, 127.5, 127.5 (Ar), 104.9 (C1′), 80.4 (C4′), 79.7 (C2′), 78.5 (C2), 74.8 (C3′), 73.4, 73.2, 73.0, 72.4, 72.1 (5 × Ph*C*H_2_), 71.5 (C5′), 70.2 (C1), 70.2 (C3).

### 4.9 1,3-Dihydroxypropan-2-yl α-ᴅ-ribofuranoside (**3**)

1,3-Bis(benzyloxy)propan-2-yl 2,3,5-tri-*O*-benzyl-α-ᴅ-ribofuranoside (**19a**) (80 mg, 120 μmol) was dissolved in MeOH-EtOH (5:1, 12 mL) and palladium on activated charcoal (10% Pd basis) (50 mg) was added. The system was flushed with N_2_ ( × 3) followed by H_2_ ( × 3) and stirred overnight at room temperature. After the system had been flushed with N_2_ ( × 3) the catalyst was filtered through Celite^®^ and the filter was washed with AcOH (50 mL). The organic washes were combined and the solvent was removed under reduced pressure to give **3** (20 mg, 75%); [α]_D_ +68.2 (*c* 1.0, MeOH); ^1^H NMR (400 MHz, CD_3_OD): 5.11 (d, *J*_1′,2′_ = 4.3 Hz, 1H, H-1′) 4.01–3.89 (m, 3H, H-4′,2′,3′), 3.72–3.47 (m, 7H, H-2,5′a,5′b,1,3); ^13^C NMR (100.6 MHz, CD_3_OD): 102.0 (C1′), 85.9 (C4′), 79.0 (C2), 71.9 (C2′), 70.1 (C3′), 61.9 (C5′), 61.5 (C1), 60.8 (C3); HRMS (ESI^+^) m/z calc. for C_8_H_16_O_7_Na^+^ 247.0794 [M+Na]^+^ found 247.0798 [M+Na]^+^.

### 4.10 1,3-Bis(benzyloxy)propan-2-yl 2,3,5-tri-*O*-benzyl-α-ʟ-xylofuranoside (**20a**) and 1,3-bis(benzyloxy)propan-2-yl 2,3,5-tri-*O*-benzyl-β-ʟ-xylofuranoside (**20b**)

2,3,5-Tri-*O*-benzyl-α/β-ʟ-xylofuranosyl fluoride (**17**) (200 mg, 0.47 mmol) and 1,3-di-*O*-benzyl glycerol (**10**) (100 μL, 0.4 mmol) were dissolved into Et_2_O (5 mL) containing 4 Å MS (1.0 g). The suspension was cooled in an ice bath and SnCl_2_ (90 mg, 0.47 mmol) was added in a single portion to initiate the reaction. The reaction mixture was then kept overnight at 4 °C, filtered and the filtrate was concentrated *in vacuo* to give a crude product, which contained a mixture of α/β -glycosides in 1.0:0.8 ratio as judged by ^1^H NMR. Purification by FCC afforded **20a** and **20b** as colourless oils.

The α-anomer **20a**: colourless oil; yield 59 mg, (19%); R_f_ 0.48 (hexane/Et_2_O 7:3); [α]_D_ −39.6 (*c* 1.0, DCM); ^1^H NMR (400 MHz, CDCl_3_): 7.31–7.22 (m, 25H, Ar), 5.35 (d, *J*_1′,2′_ = 4.3 Hz, 1H, H-1′), 4.71 (d, ^2^*J* = 11.9 Hz, 1H, PhC*H*H), 4.63 (d, ^2^*J* = 11.9 Hz, 1H, PhCH*H*), 4.57 (d, ^2^*J* = 12.1 Hz, 1H, PhC*H*H), 4.53–4.45 (m, 7H, PhCH_2_), 4.43–4.41 (m, 1H, H-4′), 4.31 (dd, *J*_2′,3′_ = 5.9 Hz, *J*_3′,4′_ = 7.0 Hz, 1H, H-3′), 4.20–4.15 (m, 1H, H-2), 3.97 (dd, *J*_1′,2′_ = 4.3 Hz, *J*_2′,3′_ = 5.9 Hz, 1H, H-2′), 3.74–3.54 (m, 6H, H-5′a,1,3,5′b); ^13^C NMR (100.6 MHz, CDCl_3_): 138.4, 138.4, 138.3, 138.3, 137.9, 128.4, 128.4, 128.3, 128.3, 128.0, 127.7, 127.7, 127.6, 127.6, 127.5, 127.5 (Ar), 99.9 (C1′), 83.8 (C2), 81.6 (C3′), 76.0 (C4′), 75.4 (C2), 73.4, 73.4, 73.3, 72.4, 71.8 (4 × Ph*C*H_2_), 71.5 (C1), 70.4 (C3), 69.4 (C5′); HRMS (ESI^+^) m/z calc. for C_43_H_46_O_7_Na^+^ 697.3141 ([M+Na]^+^) found 697.3129 [M+Na]^+^.

The β-anomer **20b**: colourless oil; yield 32 mg(12%); R_f_ 0.54 (hexane/Et_2_O 7:3); [α]_D_ +5.6 (*c* 1.0, DCM); ^1^H NMR (400 MHz, CDCl_3_): 7.32–7.23 (m, 25H, Ar), 5.31 (d, *J*_1′,2’_ = 1.9 Hz, 1H, H-1′), 4.58–4.40 (m, 11H, 5 × PhCH_2_, H-4′), 4.11–4.06 (m, 3H, H-2′,3′,2), 3.76 (dd, *J*_4′,5a′_ = 4.9 Hz, ^2^*J*_5′a,5′b_ = 10.3 Hz, 1H, H-5′a), 3.71 (dd, *J*_4′,5′b_ = 3.7 Hz, ^2^*J*_5′a,5′b_ = 10.3 Hz, 1H, H-5′b), 3.71–3.55 (m, 4H, H-1,3); ^13^C NMR (100.6 MHz, CDCl_3_): 138.5, 138.4, 138.3, 138.0, 137.7, 128.4, 128.3, 128.3, 128.3, 127.8, 127.7, 127.7, 127.6, 127.6, 127.6, 127.5, 127.5, 127.5 (Ar), 107.0 (C1′), 86.8 (C2′), 82.0 (C3′), 79.9 (C4′), 75.7 (C2), 73.4, 73.3, 73.3, 72.0, 71.8 (5 × Ph*C*H_2_), 70.7 (C1), 70.4 (C3), 69.8 (C5′);

### 4.11 1,3-Dihydroxypropan-2-yl α-ʟ-xylofuranoside (**4**)

1,3-Bis(benzyloxy)propan-2-yl 2,3,5-tri-*O*-benzyl-α-ʟ-xylofuranoside (**20a**) (100 mg, 145 μmol) was dissolved in a mixture of MeOH/n-PrOH (9:1) (20 mL) and palladium on activated charcoal (10% Pd basis) (50 mg) was added. The system was flushed with N_2_ ( × 3) followed by H_2_ ( × 3) and stirred overnight at room temperature. After the system had been flushed with N_2_ ( × 3) the catalyst was filtered through Celite^®^ and the filter was washed with MeOH (20 mL). The organic washes were combined and the solvent was removed under reduced pressure to give **4** (16 mg, 45%) as a colourless oil; R_f_ 0.28 (DCM/MeOH 85:15); [α]_D_ −139 (*c* 1.0 MeOH); ^1^H NMR (400 MHz, CD_3_OD): 5.09 (d, *J*_1′2′_ = 4.4 Hz, 1H, H-1′), 4.16–4.08 (m, 2H, H-3′,4′), 3.92 (dd, *J*_1′2′_ = 4.4 Hz, *J*_2′,3′_ = 4.4 Hz, 1H, H-2′), 3.65–3.51 (m, 7H, H-2,1,3,5′); ^13^C NMR (100.6 MHz, CD_3_OD): 101.3 (C1′), 80.0 (C2), 78.7 (C4′), 78.1 (C2′), 75.7 (C3′), 61.7 (C5′), 61.2 (C1), 61.0 (C3); (HRMS ESI^+^) m/z calc for C_8_H_16_NaO_7_^+^ 247.0794 ([M+Na]^+^) found 247.0785 [M+Na]^+^.

### 4.12 1,3-Bis(benzyloxy)propan-2-yl 2,3,5-tri-*O*-benzyl-β-ᴅ-arabinofuranoside (**21a**) and 1,3-bis(benzyloxy)propan-2-yl 2,3,5-tri-*O*-benzyl-α-ᴅ-arabinofuranoside (**21b**)

2,3,5-Tri-*O*-benzyl-α,β-ᴅ-arabinofuranosyl fluoride (**18**) (950 mg, 2.3 mmol) and 1,3-di-*O*-benzyl glycerol (**9**) (540 μL, 2.2 mmol) were dissolved into a suspension of 4 Å MS (1.0 g) in Et_2_O (7 mL). The suspension was cooled in an ice bath and SnCl_2_ (440 mg, 2.3 mmol) was added in a single portion to initiate the reaction. The reaction mixture was then kept in a refrigerator overnight at 4 °C, the solid materials were filtered off and the solvent removed *in vacuo* to give a mixture of anomers which had very close R_f_. Three rounds of purification by FCC gave the β-anomer **21a** (560 mg), α-anomer **21b** (47 mg) and some remaining unseparated mixture of both.

Compound **21a**: colourless oil; R_f_ 0.46 (hexane/Et_2_O 6:4); [α]_D_ −38.7 (*c* 1.0, CHCl_3_); ^1^H NMR (400 MHz, CDCl_3_): 7.32–7.24 (m, 25H, Ar), 5.32 (d, *J*_1′,2′_ = 4.4 Hz, 1H, H-1′), 4.71–4.36 (m, 10H, 5 × PhC*H*_2_), 4.13–4.06 (m, 3H, H-3′,4′,2), 4.03 (dd, *J*_1′,2′_ = 4.4 Hz, *J*_2′,3′_ = 7.0 Hz, 1H, H-2′), 3.71 (dd, *J*_4′,5′_ = 3.4 Hz, ^2^*J*_5a′,5b′_ = 10.3 Hz, 1H, H-5a′), 3.64–3.47 (m, 5H, H-5b′,1,3); ^13^C NMR (100.6 MHz, CDCl_3_): 138.4, 138.3, 138.3, 138.2, 137.9 (5 × Ar), 128.4, 128.4, 128.4, 128.3, 128.0, 127.8, 127.7, 127.7, 127.7, 127.6, 127.6, 127.6, (Ar), 100.8 (C1′), 83.7 (C2′), 83.1 (C3′), 80.1 (C4′), 75.6 (C2), 73.5, 73.3, 73.1 (3 × Ph*C*H_2_), 72.6 (C1), 72.3, 71.8 (2 × Ph*C*H_2_), 71.3 (C5′), 70.3 (C3); HRMS (ESI^+^) m/z calc. for C_43_H_46_O_7_ 697.3136 [M+Na]^+^ found 697.3132 [M+Na]^+^.

Compound **21b**: colourless oil; R_f_ 0.46 (hexane/Et_2_O 6:4); [α]_D_ + 26.8 (*c* 1.0, CHCl_3_); ^1^H NMR (400 MHz, CDCl_3_): 7.35–7.22 (m, 25H, Ar), 5.35 (bs, 1H, H-1′), 4.57–4.41 (m, 10H, 5 × PhC*H*_2_), 4.23 (dm, *J*_3′,4′_ = 7.3 Hz, 1H, H-4′), 4.15–4.09 (m, 1H, H-2), 4.08 (d, *J*_2′,3′_ = 3.4 Hz, 1H, H-2′), 3.92 (dd, *J*_2′,3′_ = 3.4 Hz, *J*_3′,4′_ = 7.3 Hz, 1H, H-3′), 3.70 (dd, *J*_1,2_ = 3.8 Hz, ^2^*J*_1a,1b_ = 10.2 Hz, 1H, H-1a), 3.65–3.54 (m, 5H, H-1b,3,5′); ^13^C NMR (100.6 MHz, CDCl_3_): 138.4, 138.3, 138.2, 138.0, 137.7 (5 × Ar), 128.4, 128.3, 128.0, 127.8, 127.7, 127.7, 127.6, 127.6, 127.5 (Ar), 106.1 (C1′), 88.5 (C2′), 83.7 (C3′), 80.3 (C4′), 75.0 (C2), 73.4 73.3, 73.3, 72.1, 71.8 (5 × Ph*C*H_2_), 70.8 (C1), 70.5 (C3), 69.6 (C5′); HRMS (ESI^+^) m/z calc. for C_43_H_46_O_7_ 697.3136 [M+Na]^+^ found 697.3132 [M+Na]^+^.

### 4.13 1,3-Dihydroxypropan-2-yl β-ᴅ-arabinofuranoside (**5**)

1,3-Bis(benzyloxy)propan-2-yl 2,3,5-tri-*O*-benzyl-β-ᴅ-arabinofuranoside (**21a**) (560 mg, 0.8 mmol) was dissolved in a mixture of MeOH/EtOAc (9:1) (50 mL) and palladium on activated charcoal (10% Pd basis) (50 mg) was added. The system was flushed with N_2_ ( × 3) followed by H_2_ ( × 3) and stirred for 48 h at room temperature. After the system had been flushed with N_2_ ( × 3) the catalyst was filtered through Celite^®^ and the filter was washed with MeOH (20 mL). The organic washes were combined and the solvent was removed under reduced pressure to give **5** (80 mg, 45%) as a colourless oil; R_f_ 0.45 (DCM/MeOH 85:15); [α]_D_ −78.1 (*c* 1.0, MeOH); ^1^H NMR (400 MHz, CD_3_OD): 5.05 (d, *J*_1′,2′_ = 4.7 Hz, 1H, H-1′), 4.18 (dd, *J*_2′,3′_ = 8.0 Hz, *J*_3′,4′_ = 8.0 Hz, 1H, H-3′), 4.00 (dd, *J*_1′,2′_ = 4.7 Hz, *J*_2′,3′_ = 8.0 Hz, 1H, H-2′), 3.78–3.73 (m, 3H, H-1,4′), 3.72–3.68 (m, 1H, H-2), 3.66–3.61 (m, 4H, H-5′,3); ^13^C NMR (100.6 MHz, CD_3_OD): 101.3 (C1′), 82.5 (C2), 80.8 (C4′), 77.6 (C2′), 73.5 (C3′), 61.7 (C5′), 61.4 (C1,C3); HRMS (ESI^+^) m/z calc. for C_8_H_16_O_7_Na^+^ 247.0794 [M+Na]^+^ found 247.0788 [M+Na]^+^.

### 4.14 Prop-2-en-1-yl 2,3,5-tri-*O*-benzoyl-α-ʟ-arabinopyranoside (**22**)

A mixture of 2,3,4-tri-*O*-benzoyl-β-ʟ-arabinopyranosyl bromide (**13**) [25] (1.8 g,3.5 mmol), allyl alcohol (290 μL, 4.2 mmol) and 4 Å MS (2.0 g) in DCE (30 mL) was stirred at room temperature for 30 min. Silver carbonate (1.2 g, 4.2 mmol) was added and stirring continued in the dark at room temperature overnight. The reaction mixture was filtered through Celite^®^ and the volatile components were evaporated *in vacuo* to give a crude syrup which was purified by FCC to give α-glycoside **22** (1.1 g, 62%) as a colourless oil; R_f_ 0.64 (hexane/EtOAc 7:3); [α]_D_ +106° (*c* = 1.0, CHCl_3_); ^1^H NMR (400 MHz, CDCl_3_): 8.05–8.01 (m, 4H, Ar), 7.95–7.93 (m, 2H, Ar), 7.59–7.31 (m, 9H, Ar), 5.86 (m, 1H, H-2), 5.74 (dd, *J*_1′,2′_ = 6.1 Hz, *J*_2′,3′_ = 8.7 Hz, 1H, H-2′), 5.71–5.68 (m, 1H, H-4′), 5.62 (dd, *J*_2′,3’_ = 8.7 Hz, *J*_3′,4’_ = 3.4 Hz, 1H, H-3′), 5.29 (dq, *J*_2,3a_ = 17.3 Hz, ^2^*J*_3a,3b_ = 1.7 Hz, 1H, H-3a), 5.17 (dq, *J*_2,3b_ = 10.5 Hz, ^2^*J*_3a,3b_ = 1.7 Hz, 1H, H-3b), 4.82 (d, *J*_1′,2′_ = 6.1 Hz, 1H, H-1′), 4.38 (ddt, *J*_1a,1b_ = 13.1 Hz, *J*_1a,2_ = 5.0 Hz, ^4^*J*_1a,3_ = 1.6 Hz, 1H, H-1a), 4.33 (dd, *J*_4,5a′_ = 4.4 Hz, ^2^*J*_5a′,5b′_ = 12.7 Hz, 1H, H-5a′), 4.16 (ddt, *J*_1a,1b_ = 13.1 Hz, *J*_1b,2_ = 6.2 Hz, ^4^*J*_1b,3_ = 1.3 Hz, 1H, H-1b), 3.90 (dd, *J*_4′,5b′_ = 2.3 Hz, ^2^*J*_5a′,5b′_ = 12.7 Hz, 1H, H-5b′); ^13^C NMR (100.6 MHz, CDCl_3_): 165.7, 165.6, 165.3 (3 × C=O), 133.5 (C2), 133.4, 133.3, 133.3, 129.9, 129.9, 129.8, 129.8, 129.4, 129.4, 129.1, 128.5, 128.5, 128.4, 117.8 (C3), 99.4 (C1′), 70.5 (C3′), 70.0 (C2′), 69.7 (C1), 68.3 (C4′), 62.3 (C5′); HRMS (ESI^+^) m/z calc. for C_29_H_26_O_8_Na^+^ 525.1525 [M+Na]^+^ found 525.1522 [M+Na]^+^.

### 4.15 (*R,S*)*-*Oxiranyl)methyl 2,3,5-tri-*O*-benzoyl-α-ʟ-arabinopyranoside (**23**)

Prop-2-en-1-yl 2,3,5-tri-*O*-benzoyl-α-ʟ-arabinopyranoside (**22**) (1.1 g, 2.2 mmol) and mCPBA (450 mg, 2.6 mmol) were dissolved into DCE (20 mL) and heated to 80 °C overnight. The solvent was removed *in vacuo*, the crude product was dissolved into EtOAc (10 mL) and washed with sat. NaHCO_3_ solution (3 × 3 mL). The organic layer was separated and dried over MgSO_4_, then filtered and the solvent evaporated *in vacuo* to give **23** (640 mg, 56%) as a white powder (1:1 *R,S*-mixture, as judged by the integration of the H-1′ signals at 4.92 and 4.82 ppm). R_f_ 0.5 (hexane/EtOAc 7:3); ^1^H NMR (400 MHz, CDCl_3_): 8.08–7.94 (m, 12H, Ar), 7.60–7.32 (m, 18H, Ar), 5.73 (dd, *J*_1′,2′_ = 6.0 Hz, *J*_2′,3′_ = 8.4 Hz, 2H, H-2′,2′*), 5.70–5.69 (m, 2H, H-4′,4′*), 5.62 (dd, *J*_2′3′_ = 8.4 Hz, *J*_3′,4′_ = 3.4 Hz, 2H, H-3′,3′*), 4.92 (d, *J*_1′a,2′_ = 6.0 Hz, 1H, H-1′), 4.82 (d, *J*_1′b,2′_ = 6.0 Hz, 1H, H-1′*), 4.37–4.31 (m, 2H, H-5a′,5a′*), 4.09 (dd, *J*_1a,1b_ = 12.0 Hz, *J*_1a,2_ = 3.0 Hz, 1H, H-1a), 3.92–3.83 (m, 4H, H-5b′,5b′*,1a*, 1b*), 3.19–3.12 (m, 2H, H-2, 2*), 2.76–2.73 (m, 2H, H-3a,3a*), 2.62 (dd, ^2^*J*_3b*,3a*_ = 5.1 Hz, *J*_3b*,2*_ = 2.6 Hz, 1H, H-3b*), 2.58 (dd, ^2^*J*_3b*,3a*_ = 5.1 Hz, *J*_3a*,2*_ = 2.6 Hz, 1H, H-3a*); ^13^C NMR (100.6 MHz, CDCl_3_): 165.7, 165.6, 165.3, (3 × C=O), 133.4, 133.4, 133.3, 130.2, 129.9, 129.8, 129.4, 129.3, 129.1, 128.5, 128.4, 128.3, 100.5 (C1′), 100.2 (C1′*), 70.4 (C3′), 70.2 (C3′*), 69.9, (C2′), 69.9 (C2′*), 69.9 (C1), 68.9 (C1*), 68.2 (C4), 68.1 (C4*), 50.7 (C2), 50.4 (C2*), 44.3 (C3), 44.1 (C3*); HRMS (ESI^+^) m/z calc. for C_29_H_26_O_9_Na^+^ 541.1469 [M+Na]^+^ found 541.1467 [M+Na]^+^. Two sets of signals belonging to spin systems of two individual diastereomers are distinguished by marking one of the sets with asterisk.

### 4.16 3-(3-Azidopropoxy)-2-(*R,S*)-hydroxypropyl 2,3,4-tri-*O*-benzoyl-α-ʟ-arabinopyranoside (**24**)

(*R,S*)*-*Oxiranyl)methyl 2,3,5-tri-*O*-benzoyl-α-ʟ-arabinopyranoside (**23**) (1.0 g, 1.9 mmol), 3-azido propanol (230 μL, 2.5 mmol) and Sc(OTf)_3_ (140 mg, 0.3 mmol) were dissolved into toluene (100 mL) and stirred vigorously at room temperature overnight. The reaction mixture was then washed with sat. NaHCO_3_ solution (3 × 30 mL) and the organic layer dried over MgSO_4_, filtered and the solvent evaporated *in vacuo*. The crude product was then purified by FCC to give **24** (480 mg, 40%) as a colourless oil (1:1 mixture of diastereoisomers as judged by the integration of the ^1^H signals at 4.81 and 4.79 ppm). R_f_ 0.27 (hexane/EtOAc 7:3); ^1^H NMR (400 MHz, CDCl_3_): 8.08–8.00 (m, 8H, Ar), 7.93–7.90 (m, 4H, Ar), 7.61–7.30 (m, 18H, Ar), 5.74 (dd, *J*_1′,2′_ = 6.5 Hz, *J*_2′,3′_ = 8.9 Hz, 2H, H-2′, H-2′*), 5.71–5.67 (m, 2H, H-4′, H-4′*), 5.61 (dd, *J*_2′,3′_ = 8.9 Hz, *J*_3′,4′_ = 3.5 Hz, 2H, H-3′,3′*), 4.81 (d, *J*_1′,2′_ = 6.5 Hz, 1H, H-1′), 4.79 (d, *J*_1′*,2′*_ = 6.5 Hz, 1H, H-1′*), 4.34 (dd, *J*_5′a,5′b_ = 12.9 Hz, *J*_*4′,5′a*_ = 3.7 Hz, 2H, H-5′a,5′a*), 3.98–3.86 (m, 6H, H-5′b,5′b*,2,2*,3a,3a*), 3.75 (dd, ^2^*J*_3a,3b_ = 10.2 Hz, *J*_2,3a_ = 4.4 Hz, 1H, H-3b), 3.67 (dd, ^2^*J*_3a*,3b*_ = 9.8 Hz, *J*_2,3a*_ = 3.5 Hz, 1H, H-3b*), 3.45–3.28 (m, 10H, H-4,1a,1a*,1b,1b*,6,6*), 2.35 (s, 1H, OH), 2.17 (s, 1H, OH*), 1.78–1.73 (m, 2H, H-5,5*); ^13^C NMR (100.6 MHz, CDCl_3_): 165.7, 165.6, 165.3 (3 × C=O), 133.5, 133.4, 129.9, 129.9, 129.8, 129.4, 129.3, 129.2, 129.0, 128.5, 128.5, 128.4, 128.2, 101.5 (C1), 101.4 (C1*), 71.6 (C4), 71.6 (C4*), 71.3 (C3), 71.1 (C3*), 70.6 (C3′3′*), 69.3 (C2), 69.3 (C2*), 68.4 (C4′,4′*), 68.0 (C1,1*), 63.0 (C5′,5′*), 48.4 (C6,6*), 29.0 (C5), 28.9 (C5*); HRMS (ESI^+^) m/z calc. for C_32_H_33_N_3_O_10_Na^+^ 642.2058 [M+Na]^+^ found 642.2051 [M+Na]^+^. Two sets of signals belonging to spin systems of two individual diastereomers are distinguished by marking one of the sets with asterisk.

### 4.17 (2R)-3-(3-Azidopropoxy)-2-(2,3,5-tri-*O*-benzyl-α-ᴅ-ribofuranosyloxy)propyl 2,3,4-tri-*O*-benzoyl-α-ʟ-arabinopyranoside (27) and (2*S*)-3-(3-azidopropoxy)-2-(2,3,5-tri-*O*-benzyl-α-ᴅ-ribofuranosyloxy)propyl- 2,3,4-tri-*O*-benzoyl-α-ʟ-arabinopyranoside (**28**)

A suspension of 3-(3-azidopropoxy)-2-hydroxypropyl 2,3,4-tri-*O*-benzoyl-α-ʟ-arabinopyranoside (**24**) (200 mg, 0.3 mmol), 2,3,5-tri-*O*-benzyl-1-(*N*-phenyl)-2,2,2-trifluoroacetimidoyl)-β-ᴅ-ribofuranose (**25**) [40] (270 mg, 0.45 mmol) and 4 Å MS (1.0 g) in dry dichloromethane (10 mL was stirred under a nitrogen atmosphere for 30 min. The mixture was then cooled to −78 °C and TMSOTf (11 μL, 60 μm mol) was added by syringe. After 2 h of stirring at −78 °C, the reaction was quenched with Et_3_N (20 μL) and the solvents were removed *in vacuo*. Excess of the acceptor was removed by FCC and the diastereomeric products were then separated by preparative TLC (hexane/EtOAc 6:4) to give **27** (45 mg, 15%) and **28** (30 mg, 10%) as colourless oils.

Compound **27**: R_f_ 0.45 (hexane/EtOAc 6:4); [α]_D_ + 95.9 (*c* 1.0, CHCl_3_); ^1^H NMR (400 MHz, CDCl_3_): 8.01–8.00 (m, 4H, Bz), 7.87 (dd, *J* = 8.2 Hz, *J* = 1.0 Hz, 2H, Bz), 7.56–7.22 (m, 24H, Ar), 5.74 (dd, *J*_1′,2′_ = 7.0 Hz, *J*_2′,3′_ = 9.4 Hz, 1H, H-2′), 5.60–5.58 (m, 1H, H-4′), 5.52 (dd, *J*_2′,3′_ = 9.4 Hz, *J*_3′,4′_ = 3.6 Hz, 1H, H-3′), 5.21 (d, *J*_1″,2″_ = 4.2 Hz, 1H, H-1″), 4.88 (d, *J*_1′,2′_ = 7.0 Hz, 1H, H-1′), 4.71 (d, ^2^*J* = 12.1 Hz, 1H, C*H*HPh), 4.69 (d, ^2^*J* = 12.1 Hz, 1H, CH*H*Ph), 4.56 (d, ^2^*J* = 11.7 Hz, 1H, C*H*HPh), 4.53 (d, ^2^*J* = 11.7 Hz, 1H, CH*H*Ph), 4.50 (d, ^2^*J* = 11.9 Hz, 1H, C*H*HPh), 4.44 (d, ^2^*J* = 11.9 Hz, 1H, CH*H*Ph), 4.24 (dd, *J*_3″,4″_ = 8.3 Hz, *J*_4″,5″_ = 4.0 Hz, 1H, H-4″), 4.18 (dd, *J*_4′,5a′_ = 3.3 Hz, ^2^*J*_5a′,5b′_ = 13.1 Hz, 1H, H-5a′), 4.02–3.95 (m, 2H, H-3a,2), 3.85–3.79 (m, 2H, H-3b,3″), 3.72–3.69 (m, 2H, H-5b′,2″), 3.50 (dd, *J*_4″,5″a_ = 5.0 Hz, ^2^*J*_5a″,5b″_ = 10.3 Hz, 1H, H-5a″), 3.45–3.39 (m, 2H, H-5b″,1), 3.34–3.27 (m, 2H, H-4), 3.22 (t, *J*_5,6_ = 7.0 Hz, 2H, H-6), 1.67–1.61 (m, 2H, H-5); ^13^C NMR (100.6 MHz, CDCl_3_): 165.7, 165.6, 165.3 (3 × C=O), 133.3, 133.3, 129.9, 129.8, 129.4129.4, 129.1, 128.5, 128.4, 128.4, 128.3, 128.1, 128.0, 127.7, 127.7, 127.6, 101.5 (C1″), 101.1 (C1′), 81.6 (C4″), 77.7 (C2″), 75.7 (C2), 75.5 (C3″), 73.5 (CH_2_Ph), 72.3 (CH_2_Ph), 72.3 (CH_2_Ph), 71.0 (C3′), 70.6 (C5″), 70.2 (C2′), 70.0 (C1), 69.2 (C3), 68.9 (C4′), 67.9 (C4), 63.3 (C5′), 48.4 (C6), 29.0 (C5); HRMS (ESI^+^) m/z calc. for C_58_H_59_N_3_O_14_Na^+^1044.3889 [M+Na]^+^ found 1044.3885 [M+Na]^+^.

Compound **28**: R_f_ 0.53 (hexane-EtOAc 6:4); [α]_D_ +120 (*c* 1.0, CHCl_3_); ^1^H NMR (400 MHz, CDCl_3_): 8.04–8.00 (m, 4H, Bz), 7.93 (dd, *J* = 8.2 Hz, *J* = 1.1 Hz, 2H, Bz), 7.57–7.19 (m, 24H, Ar), 5.67 (dd, *J*_1′,2′_ = 6.0 Hz, *J*_2′,3′_ = 7.9 Hz, 1H, H-2′), 5.56 (m, 2H, H-4′,3′), 5.23 (d, *J*_1″,2″_ = 4.4 Hz, 1H, H-1″), 4.91 (d, *J*_1′,2′_ = 6.0 Hz, 1H, H-1′), 4.69 (d, ^2^*J*_CHHPh, CHHPh_ = 10.0 Hz, 1H, C*H*HPh), 4.66 (d, ^2^*J*_CHHPh, CHHPh_ = 10.0 Hz, 1H, CH*H*Ph), 4.56 (d, ^2^*J*_CHHPh, CHHPh_ = 15.2 Hz, 1H, C*H*HPh), 4.53 (d, ^2^*J*_CHHPh, CHHPh_ = 15.2 Hz, 1H, CH*H*Ph), 4.46 (d, ^2^*J*_CHHPh, CHHPh_ = 12.2 Hz, 1H, C*H*HPh), 4.40 (d, ^2^*J*_CHHPh, CHHPh_ = 12.2 Hz, 1H, CH*H*Ph), 4.27 (dd, *J*_3″,4″_ = 7.4 Hz, *J*_4″,5″_ = 3.7 Hz, 1H, H-4″), 4.20 (dd, *J*_4′,5a′_ = 3.8 Hz, ^2^*J*_5a′,5b′_ = 12.8 Hz, 1H, H-5a′), 4.03–4.00 (m, 1H, H-2), 3.92 (dd, *J*_2,3a_ = 6.3, *J*_3a,3b_ = 10.9, 1H, H-3a), 3.86–3.81 (m, 2H, H-3″,3b), 3.77 (dd, *J*_1″,2″_ = 4.4 Hz, *J*_2″,3″_ = 6.5 Hz, 1H, H-2″), 3.72 (dd, *J*_4′,5b′_ = 1.8 Hz, ^2^*J*_5a′,5b′_ = 12.8 Hz, 1H, H-5b′), 3.51 (d, *J*_1,2_ = 5.4 Hz, 2H, H-1), 3.36 (dd, *J*_4″,5″_ = 3.7 Hz, ^2^*J*_5a″,5b″_ = 3.7 Hz, 2H, H-5a″,5b″), 3.34–3.26 (m, 2H, H-4), 3.27 (t, *J*_5,6_ = 6.8 Hz, 2H, H-6), 1.69–1.62 (m, 2H, H-5); ^13^C NMR (100.6 MHz, CDCl_3_): 165.7, 165.6, 165.3 (3 × C=O), 138.4, 138.1, 138.0, 133.3, 133.3, 129.9, 129.9, 129.5, 129.2, 128.5, 128.4, 128.4, 128.4, 128.4, 128.3, 128.1, 127.8, 127.7, 127.7, 127.6, 127.6, 127.6, 101.4 (C1″), 100.5 (C1′), 81.8 (C4″), 75.7 (C2), 75.6 (C3″), 74.4 (CH_2_Ph), 72.4 (CH_2_Ph), 72.2 (CH_2_Ph), 71.5 (C1), 70.5 (C3′), 70.1 (C2′), 70.0 (C5″), 67.0 (C3), 68.5 (C4′), 67.9 (C4), 62.2 (C5′), 48.4 (C6), 29.1 (C5); HRMS (ESI^+^) m/z calc. for C_58_H_59_N_3_O_14_Na^+^ 1044.3889 [M+Na]^+^ found 1044.3890 [M+Na]^+^.

### 4.18 General procedure for deprotection of compounds **27** and **28**

(2*R*)- or (2*S*)-3-(3-Azidopropoxy)-2-(2,3,5-tri-*O*-benzyl-α-ᴅ-ribofuranosyloxypropyl 2,3,4-tri-*O*-benzoyl-α-ʟ-arabinopyranoside (**27**) or (**28**) respectively was dissolved in a mixture of MeOH (10 mL) and EtOAc (5 mL) and hydrogenated over 10% Pd/C (5 mg) under a H_2_ at 50 °C for 24 h. The resulting mixture was filtered through Celite^®^, the filter was washed with additional MeOH (25 mL) and the solvent was removed *in vacuo*. The residue was dissolved in a mixture of MeOH/H_2_O/Et_3_N (5:2:1 mL) and stirred at room temperature overnight. The mixture was concentrated *in vacuo* and the residue was passed through a short column of Dowex^®^ 1X2-400 resin (OH^−^ form) in water. The sample was lyophilised to give target compounds **6** and **7**.

(2*R*)-3-(3-Aminopropoxy)-2-(α-ᴅ-ribofuranosyloxy)propyl α-ʟ-arabinopyranoside (**6**) was prepared from **27** (45 mg, 44 μmol) as a white powder (10.7 mg, 59%); [α]_D_ +40.4 (*c* = 1.0, H_2_O); ^1^H NMR (400 MHz, D_2_O): 5.24 (d, *J*_1″,2″_ = 4.4 Hz, 1H, H-1″), 4.29 (d, *J*_1′,2′_ = 7.7 Hz, 1H, H-1′); 4.07 (dd, *J*_3″,4″_ = 6.6 Hz, *J*_4″,5a″_ = 3.7 Hz, 1H, H-4″), 4.04–4.00 (m, 2H, H-4′,2″), 3.95 (dd, *J*_2″,3″_ = 3.5 Hz, *J*_3″,4″_ = 6.6 Hz, 1H, H-3″), 3.90–3.83 (m, 4H, H-3a, 2, 5a′), 3.75 (dd, *J*_2,3b_ = 4.4 Hz, *J*_3a,3b_ = 11.4 Hz, 1H, H-3b), 3.68 (dd, *J*_4″,5a″_ = 3.7 Hz, *J*_5a″,5b″_ = 12.4 Hz), 1H, H-5a″), 3.63–3.46 (m, 8H, H-1,5b″,3′,5′,4,2′), 2.60 (t, *J*_5,6_ = 6.9 Hz, 1H, H-6), 1.70–1.61 (m, 1H, H-5); ^13^C NMR (100.6 MHz, D_2_O): 103.4 (C1′), 101.9 (C1″), 84.7 (C4″) 75.7 (C4′), 72.3 (C3′), 71.3 (C2″), 70.7 (C2′), 70.2 (C1), 69.6 (C3″), 69.3 (C4), 69.1 (C3), 68.2 (C2), 66.2 (C5′), 61.5 (C5″), 37.7 (C6), 31.4 (C5); HRMS (ESI^+^) m/z calc. for C_16_H_31_NO_11_Na^+^ 414.1970 [M+Na]^+^ found 414.1968 [M+Na]^+^.

(2*S*)-3-(3-Aminopropoxy)-2-(α-ᴅ-ribofuranosyloxypropyl) α-ʟ-arabinopyranoside (**7**) was prepared by deprotection of (**28**) (30 mg, 29 μmol) as a white powder; yield 7.4 mg (41%); [α]_D_ +35.5 (*c* 1.0, H_2_O); ^1^H NMR (400 MHz, D_2_O): 5.24 (t, *J*_1″,2″_ = 3.9 Hz, 1H, H-1″), 4.29 (d, *J*_1′,2′_ = 7.6 Hz, 1H, H-1′), 4.11 (dd, *J*_3″,4″_ = 7.6 Hz, *J*_4″,5″_ = 3.7 Hz, 1H, H-4″), 4.05–3.92 (m, 4H, H-4′,2″,3″,3a), 3.87–3.82 (m, 2H, H-5′,2), 3.74 (dd, *J*_2,3a_ = 4.9 Hz, *J*_3a,3b_ = 10.5 Hz, 1H, H-3b), 3.70–3.47 (m, 8H, H-1,5a″,5″b,3′,5b′,4,2′), 2.83 (t, *J*_5,6_ = 7.2 Hz, 1H, H-6), 1.80–1.73 (m, 1H, H-5); ^13^C NMR (100.6 MHz, D_2_O): 103.5 (C1′), 102.0 (C1″), 84.7 (C4″), 75.7 (C4′), 72.3 (C3′), 71.4 (C2″), 70.7 (C2′), 69.7 (C1), 69.6 (C3″), 69.6 (C3), 69.1 (C4), 68.2 (C2), 66.3 (C5′), 61.4 (C5″), 37.7 (C6), 29.0 (C5); HRMS (ESI^+^) m/z calc. for C_16_H_31_NO_11_Na^+^ 414.1970 [M+Na]^+^ found 414.1969 [M+Na]^+^.

## Appendix A Supplementary data

Supporting Material_revised_V2 Supporting Material_revised_V2
