# Supplementary material for: Synthesis of glyceryl glycosides related to A-type prymnesin toxins
Source: Carbohydr Res. 2018 Jun 30;463:14–23. doi: 10.1016/j.carres.2018.04.008 (PMC5999359; doi:10.1016/j.carres.2018.04.008)
Supplement: Supporting Material_revised_V2 [file mmc1.pdf]

Supporting Material

Synthesis of glyceryl glycosides related to A-type prymnesin toxins

Edward S. Hems, Sergey A. Nepogodiev, Martin Rejzek, Robert A. Field*

Dept. of Biological Chemistry, John Innes Centre, Norwich Research Park, Colney Lane, Norwich, NR4 7UH, UK

Contents

Synthesis of Per-O-benzoyl-β-D-galactofuranose (8)[1] 3

Experimental 3

2,3,5,6-Tetra-O-benzoyl-D-galacto-1,4-lactone[2] 3

Per-O-benzoyl-β-D-galactofuranose (8)[1,2] 4

Per-O-benzoyl-β-L-arabinopyranose (12)[4] 4

2,3,4-Tri-O-benzoyl-β-L-arabinopyranosyl bromide (13)[5] 5

Trityl perchlorate ($Ph_3C^+.ClO_4^-$)[6] 5

2,3,5-Tri-O-benzyl-β-D-ribofuranosyl fluoride (16)[7] 6

2,3,5-Tri-O-benzyl-α/β-L- xylofuranosyl fluoride (17) 6

2,3,5-Tri-O-benzyl-α/β-D-arabinofuranosyl fluoride (18)[9] 7

2',3',5'-Tri-O-benzyl-β-D-ribofuranosyl (N-phenyl)-2,2,2-trifluoroacetimidate (25) [10] 8

NMR Spectra 9

1,3-Bis(benzyloxy)propan-2-yl 2,3,5,6-tetra-O-benzoyl-β-D-galactofuranoside (10) 9

1,3-Dihydroxypropan-2-yl 2,3,5,6-tetra-O-benzoyl-β-D-galactofuranoside (11) 10

1,3-Dihydroxypropan-2-yl β-D-galactofuranoside (1) 11

1,3-Bis(benzyloxy)propan-2-yl 2,3,4-tri-O-benzoyl-α-L-arabinopyranoside (14) 12

1,3-Dihydroxypropan-2-yl 2,3,4-tri-O-benzoyl-α-L-arabinopyranoside (15) 13

1,3-Dihydroxypropan-2-yl α-L-arabinopyranoside (2) 14

* Corresponding author: rob.field@jic.ac.uk

1,3-Bis(benzyloxy)propan-2-yl 2,3,5-tri-O-benzyl-α-ᴅ-ribofuranoside (19a) 15
1,3-Bis(benzyloxy)propan-2-yl 2,3,5-tri-O-benzyl-β-ᴅ-ribofuranoside (19b) 16
1,3-Dihydroxypropan-2-yl α-ᴅ-ribofuranoside (3) 17
1,3-Bis(benzyloxy)propan-2-yl 2,3,5-tri-O-benzyl-α-ʟ-xylofuranoside (20a) 18
1,3-Bis(benzyloxy)propan-2-yl 2,3,5-tri-O-benzyl-β-ʟ-xylofuranoside (20b) 19
1,3-Dihydroxypropan-2-yl α-ʟ-xylofuranoside (4) 20
1,3-Bis(benzyloxy)propan-2-yl 2,3,5-tri-O-benzyl-β-ᴅ-arabinofuranoside (21a) 21
1,3-Bis(benzyloxy)propan-2-yl 2,3,5-tri-O-benzyl-α-ᴅ-arabinofuranoside (21b) 22
1,3-Dihydroxypropan-2-yl β-ᴅ-arabinofuranoside (5) 23
Prop-2-en-1-yl 2,3,5-tri-O-benzoyl-α-ʟ-arabinopyranoside (22) 24
(R/S-Oxiranyl)methyl 2,3,5-tri-O-benzoyl-α-ʟ-arabinopyranoside (23) 25
3-(3-Azidopropoxy)-2-hydroxypropyl 2,3,4-tri-O-benzoyl-α-ʟ-arabinopyranoside (24) 26
3-(3-Azidopropoxy)-2R-[(2,3,5-tri-O-benzyl-α-ᴅ-ribofuranosyl)oxy]propyl-2,3,4-tri-O-benzoyl-α- ʟ -arabinopyranoside (27) 27
3-(3-Azidopropoxy)-2S-[(2,3,5-tri-O-benzyl-α-ᴅ-ribofuranosyl)oxy]propyl-2,3,4-tri-O-benzoyl-α- ʟ -arabinopyranoside (28) 28
(2R)-3-(3-aminopropoxy)-2-(α-ᴅ-ribofuranosyloxy)propyl α-ʟ-arabinopyranoside (6) 29
(2S)-3-(3-aminopropoxy)-2-(α-ᴅ-ribofuranosyloxy)propyl α-ʟ-arabinopyranoside (7) 30
References: 31

Synthesis of Per-*O*-benzoyl-β-ᴅ-galactofuranose (8)[1]

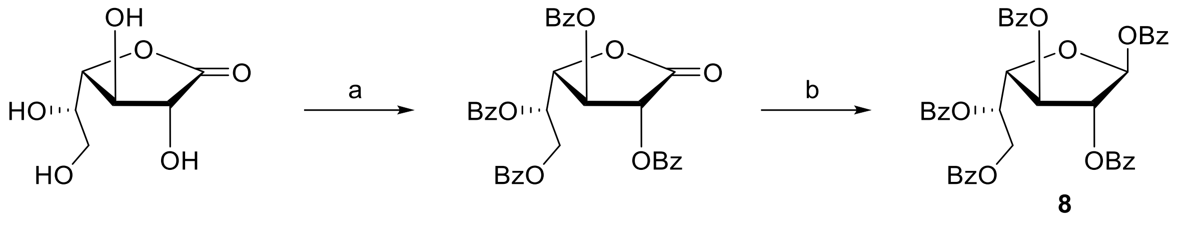

Supplemental Scheme 1 - Synthesis of per-*O*-benzoyl-β-ᴅ-galactofuranose (**8**). (a) BzCl, DMAP, Pyr, 98%; (b) i) L-Selectride®, THF; (ii) BzCl, Pyr, 76% over two steps.

Experimental

2,3,5,6-Tetra-O-benzoyl-ᴅ-galacto-1,4-lactone[2]

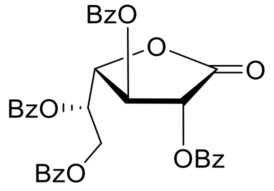

Galactono-1,4-lactone (2.00 g, 11.2 mmol) and DMAP (20mg, 160 µmol) were dissolved into dry pyridine (30 mL) under N_2. BzCl (8.0 mL, 68 mmol) was added dropwise to the solution and the reaction was stirred at room temperature for 2 hours. The reaction mixture was diluted into DCM (50 mL) and washed with HCl (1.0 M, 2 × 10 mL) and brine (10 mL) before being dried over $MgSO_4$, filtered and dried *in vacuo*. The crude residue was purified by FCC to give the title compound (6.6 g, 98%) as a yellow oil; R_f 0.45 (hexane/EtOAc 7:3); ν_{max}/cm^{-1} (FTR-IR), 1720 (C=O), 1601 (C=C) 1245 (C-O), 1091 (C-O); ^{1}H NMR (400 MHz, $CDCl_3$): 8.17-7.26 (m, 20H, Ar), 6.09 (d, $J_{2,3}$ = 5.7 Hz, 1H, H-2), 6.08-6.04 (m, 1H, H-5), 5.87 (dd, $J_{2,3}$ = 5.7 Hz, $J_{3,4}$ = 5.7 Hz, 1H, H-3), 5.06 (dd, $J_{3,4}$ = 5.7 Hz, $J_{4,5}$ = 2.7 Hz, 1H, H-4), 4.78-4.70 (m, 2H, H-6a,6b); ^{13}C NMR (100.6 MHz, $CDCl_3$): 168.7 (C1), 165.9, 165.5, 165.2, 165.0 (4 × C=O), 134.6, 134.0, 133.8, 133.8, 133.3, 130.6, 130.2, 130.1, 130.0, 129.8, 129.3, 128.9, 128.8, 128.7, 128.6, 128.5, 128.4, 128.1, 127.9 (Ar), 79.5 (C4), 74.3 (C3), 72.3 (C2), 70.1 (C5), 62.4 (C6). The ^{1}H NMR data were in accordance with the literature.[3]

Per-*O*-benzoyl-β-D-galactofuranose (8)[1,2]

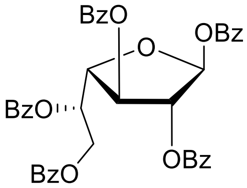

A solution of 2,3,5,6-tetra-*O*-benzoyl-D-galacto-1,4-lactone (6.0 g, 10 mmol) in THF (30 mL) was cooled to -78 °C in a bath of dry ice and acetone. L-Selectride® (1M in THF) (15 mL, 1.5 mmol) was slowly added by syringe and the reaction mixture was stirred at -78 °C for 2h, after which time analysis by TLC showed consumption of the starting lactone (R_f 0.58, hexane/EtOAc 7:3) and a new spot (R_f 0.48 hexane/EtOAc 7:3). Pyridine (4 mL) was added in a single portion followed the dropwise addition of benzoyl chloride (5 mL, 43 mmol). The reaction mixture was then removed from the dry ice bath and allowed to warm to room temperature overnight. The solvent was removed under reduced pressure and the crude reaction mixture purified by FCC to give **8** (5.4 g, 76% over 2 steps) as a white powder; R_f 0.58 (hexane/EtOAc 7:3); ^{1}H NMR (400 MHz, $CDCl_3$): 8.12-7.26 (m, 25H, Ar), 6.78 (s,1H, H-1), 6.15-6.11 (m, 1H, H-5), 5.80 (dd, $J_{2,3}$ = 0.8 Hz, $J_{3,4}$ = 4.1 Hz, 1H, H-3), 5.77 (d, $J_{2,3}$ = 0.8 Hz, 1H, H-2), 4.88 (dd, $J_{3,4}$ = $J_{4,5}$ = 4.1 Hz, 1H, H-4), 4.83-4.73 (m, 2H, H-6a,6b); ^{13}C NMR (100.6 MHz, $CDCl_3$): 166.0, 165.8, 165.4, 165.2, 164.5 (5 × C=O), 133.7, 133.7, 133.6, 133.3, 133.0, 130.0, 129.9, 129.7, 129.5, 129.4, 129.3, 128.9, 128.6, 128.5, 128.5, 128.4, 128.3, 128.2 (Ar), 99.8 (C1), 84.3 (C4), 81.0 (C2), 77.2 (C3), 70.3 (C5), 63.6 (C6); the ^{1}H NMR data were in accordance with the literature.[1]

Per-*O*-benzoyl-β-L-arabinopyranose (12)[4]

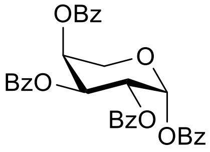

A solution of L-arabinose (1.0 g, 6.7 mmol) and DMAP (1 mol %) in dry pyridine (15 mL) was cooled in an ice bath and benzoyl chloride (5.0 mL, 43 mmol) was added dropwise over 30 minutes. The reaction was left to stir overnight at room temperature. The solvent was removed *in vacuo* and the crude mixture was dissolved in EtOAc (20 mL) and washed with 1M HCl solution (3 × 5 mL) to remove any residual pyridine. The organic layer was dried over $MgSO_4$ and the solvent was removed under reduced pressure before the crude mixture was purified by FCC to give **12** (3.40 g, 90%) as a white foam, R_f 0.4 (hexane/EtOAc 3:1); $[\alpha]_D$ +301 (c 1.0, $CHCl_3$) (lit.[4] +291.2 (*c* 0.1, $CHCl_3$)); ^{1}H NMR (400 MHz, $CDCl_3$): 8.14-8.12 (m, 4H, Ar), 7.90-7.87 (m, 4H, Ar), 7.65-7.61 (m, 2H, Ar), 7.55-7.45 (m, 6H, Ar), 7.32-7.28 (m, 4H, Ar), 6.87 (bs, 1H, H-1), 6.07-6.06 (2H, m, H-2,3), 5.90 (m, 1H, H-4), 4.42 (dd, $J_{4,5a}$ =

1.0 Hz, $^2J_{5a,5b}$ = 13.5 Hz, 1H, H-5a), 4.18 (dd, $J_{4,5b}$ = 2.1 Hz, $^2J_{5a,5b}$ = 13.5 Hz); ^{13}C NMR (100.6 MHz, $CDCl_3$): 165.8, 165.7, 165.6, 164.7 (4 × C=O), 133.8, 133.6, 133.5, 133.4, 129.9, 129.8, 129.4, 129.1, 128.9, 128.8, 128.8, 128.6, 128.4, 128.4 (Ar), 91.1 (C1), 69.5 (C4), 68.2 (C3), 67.8 (C2), 63.0 (C5). The ^{1}H and ^{13}C NMR data were in accordance with the literature.[4]

2,3,4-Tri-*O*-benzoyl-β-L-arabinopyranosyl bromide (13)[5]

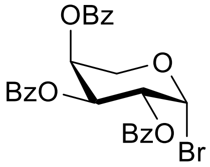

Per-*O*-benzoyl-β-L-arabinopyranose (**12**) (2.4 g, 4.2 mmol) was dissolved into dry DCM (10 mL) under N_2. A solution of 33% HBr in AcOH (2.5 mL) was added in a single portion and the reaction mixture stirred for 3 hours at room temperature after which time TLC showed consumption of the start material. The solvent was removed under reduced pressure and the reaction mixture was re-dissolved into EtOAc (10 mL) before being washed with ice cold saturated $NaHCO_3$ (3 × 3 mL). The organic layer was dried over $MgSO_4$ and the solvent removed under reduced pressure to give **13** (2.1 g, 95%) as an oil which was immediately used in the next step, R_f 0.42 (hexane/EtOAc 3:1); ^{1}H NMR (400 MHz, $CDCl_3$): 8.11-8.08 (m, 1H, Ar), 8.03-8.01 (m, 1H, Ar), 7.87-7.85 (m, 1H, Ar), 7.63-7.30 (m, 9H, Ar), 6.94 (d, $J_{1,2}$ = 3.9 Hz, 1H, H-1), 6.00 (dd, $J_{2,3}$ = 10.5 Hz, $J_{3,4}$ = 3.9 Hz, 1H, H-3), 5.84-5.83 (m, 1H, H-4), 5.71 (dd, $J_{1,2}$ = 3.9 Hz, $J_{2,3}$ = 10.5 Hz, 1H, H-2), 4.47 (dm, $^2J_{5a,5b}$ = 12.9 Hz, 1H, H-5a), 4.23 (dd, $J_{4,5b}$ = 1.9 Hz, $^2J_{5a,5b}$ = 12.9 Hz, 1H, H-5b); ^{13}C NMR (100.6 MHz, $CDCl_3$): 165.6, 165.6, 165.4 (3 × C=O), 133.8, 133.7, 133.4, 130.0, 129.9, 129.8, 128.7, 128.6, 128.4 (12 × Ar) 89.8 (C1), 68.9 (C4), 68.7 (C2), 65.0 (C3), 60.4 (C5). The ^{1}H NMR data was in agreement with literature values.[5]

Trityl perchlorate ($Ph_3C^+.ClO_4^-$)[6]

Triphenyl methanol (500 mg, 1.9 mmol) was dissolved into acetic anhydride (8 mL) and in an ice bath. 70% w/w Perchloric acid (700 µL, 8.2 mmol) was added dropwise and the reaction mixture immediately turned dark yellow. The reaction mixture was stirred for 1 hour at 0 °C before the solvent was carefully filtered off and the remaining yellow powder was rinsed with dry Et_2O (5 × 2 mL). The reaction mixture was then dried in the dark on a high vacuum line overnight to give the title compound (455 mg, 69%) as a yellow powder; ν_{max}/cm^{-1} (FTR-IR) 1579 (s), 1483 (m), 1447 (m), 1353 (s), 1294 (m), 1191 (w), 1166 (w), 1073 (broad, vs), δ_H(400 MHz; CD_3CN) 7.37-7.27 (m, 15H, Ar), δ_C(100 MHz; CD_3CN) 148.1 (Ph_3C^+), 128.4 (6 × Ar), 128.3 (6 × Ar), 127.6 (3 × Ar). The infrared spectrum is in good agreement with literature values.[6]

2,3,5-Tri-*O*-benzyl-β-D-ribofuranosyl fluoride (16)[7]

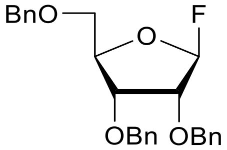

2,3,5-Tri-*O*-benzyl-D-ribofuranose (1.0 g, 2.4 mmol) was dissolved into dry THF (10 mL) and cooled to -30 °C. Diethylaminosulfur trifluoride (DAST) (0.4 mL, 2.9 mmo) was added in a single portion and the reaction mixture was allowed to warm to room temperature. After 20 minutes TLC indicated all start material had been consumed. The reaction mixture was cooled back down to -30 °C and the residual DAST was quenched with MeOH (0.5 mL). The solvent was removed under reduced pressure and the anomeric glycosyl fluorides were separated by FCC to give **16** (645 mg, 64%) as a colourless oil; R_f 0.82 (hexane/EtOAc 9:1) $[\alpha]_D$ +49.4 (c 1.0, $CHCl_3$); ^{1}H NMR (400 MHz, $CDCl_3$): 7.35-7.29 (m, 15H, Ar), 5.67 (d, $J_{1,F}$ = 63.3 Hz, 1H, H-1), 4.64-4.44 (m, 6H, PhCH_2,), 4.46-4.41 (m, 1H, H-4), 4.14-4.10 (m, 1H, H-3), 3.99 (dd, $J_{2,3}$ = 4.2 Hz, $J_{2,F}$ = 4.2 Hz, 1H, H-2), 3.68 (dd, $J_{4,5a}$ = 3.4 Hz, $^2J_{5a,5b}$ = 11.1 Hz, 1H, H-5a), 3.58 (dd, $J_{4,5b}$ = 5.3 Hz, $^2J_{5a,5b}$ = 11.1 Hz, 1H, H-5b); ^{13}C NMR (100.6 MHz, $CDCl_3$): 138.1, 137.5, 137.4 (3 × Ar), 128.6, 128.5, 128.4, 128.1, 128.0, 128.0, 127.7, 127.6 (Ar), 112.6 (d, $J_{C1,F}$ = 224 Hz, C1), 82.4 (C4), 78.9 (d, $J_{C2,F}$ = 30.1 Hz, C2), 77.1 (C3), 73.4, 72.8, 72.8 (3 × CH_2Ph), 70.2 (C5); ^{19}F NMR (376 MHz; $CDCl_3$) -115.3 (dm, $J_{1,F}$ = 63.3 Hz). The diagnostic NMR signals are in agreement with literature values.[8]

2,3,5-Tri-*O*-benzyl-α/β-L- xylofuranosyl fluoride (17)

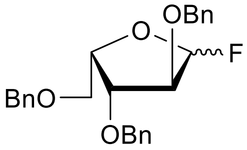

2,3,5-Tri-*O*-benzyl-L-xylofuranose (500 mg, 1.2 mmol) was dissolved in dry THF (10 mL), the solution was cooled to -30 °C and DAST (0.2 mL, 1.5 mmol) was added in a single portion. The reaction mixture was allowed to warm to room temperature and stirred for 20 minutes, after which time TLC (hexane/EtOAc 8:2) indicated the starting material had been consumed. The reaction mixture was cooled back to – 30 °C and quenched with MeOH (0.5 mL). The solvent was removed under reduced pressure and the crude mixture of anomers was passed through a short silica plug and the solvent was removed under educed pressure to give the mixture of fluoride anomers **17** (450 mg, 90%) as a colourless oil. The ratio of anomers was judged to be α/β 3.3:1 by integration of the anomeric ^{1}H NMR signals; R_f 0.31 (hexane/EtOAc 8:2); ^{1}H NMR (400 MHz, $CDCl_3$): 7.38-7.25 (m, 26H, Ar), 5.74 (d, $J_{1\beta,F}$ = 64.6 Hz, 1H, H-1β), 5.67 (dd, $J_{1\alpha,F}$ = 64.8 Hz, $J_{1\alpha,2\alpha}$ = 3.5 Hz, 0.3H, H-1α), 4.68-5.90 (m, 11.7H, H-4α,4β,PhCH_2), 4.35 (dd, $J_{2\alpha,3\alpha}$ = 6.5 Hz, $J_{3\alpha,4\beta}$ = 6.5 Hz, 0.3H, H-3α), 4.15 (d, $J_{2\beta,F}$ = 6.8 Hz, 1H, H-2β), 4.13

(ddd, $J_{2\alpha,F}$ = 18.9 Hz, $J_{1\alpha,2\alpha}$ = 3.5 Hz, $J_{2\alpha,3\alpha}$ = 6.5 Hz, 0.3H, H-2α) 4.07 (d, $J_{3\beta,4\beta}$ = 5.7 Hz, 1H, H-3β), 3.84 (dd, $J_{4\alpha,5a\alpha}$ = 5.1 Hz, $J_{5a\alpha,5b\alpha}$ = 10.3 Hz, 1H, H-5aα), 3.76 (dd, $J_{4\alpha,5b\alpha}$ = 7.0 Hz, $^{2}J_{5a\alpha,5b\alpha}$ = 10.3 Hz, 1H, H-5bα), 3.70 (dd, $J_{4\beta,5a\beta}$ = 3.9 Hz, $J_{5a\beta,5b\beta}$ = 10.9 Hz, 1H, H-5aβ), 3.60 (dd, $J_{4\beta,5b\beta}$ = 5.8 Hz, $^{2}J_{5a\beta,5b\beta}$ = 10.9 Hz, 1H, H-5bβ); ^{13}C NMR (100.6 MHz, $CDCl_3$): 138.2 (C=O β), 138.1 (C=O α), 137.8 (C=O α), 137.5 (C=O β), 134.4 (C=O α), 137.0 (C=O β), 128.6, 128.6, 128.5, 128.5, 128.4, 128.2, 128.1, 128.0, 127.9, 127.8, 127.8, 127.8, 127.7, 127.7, 127.7, 127.6 (Ar), 113.3 (d, $J_{C1\beta,F}$ = 225 Hz, C1β), 107.5 (d, $J_{C1\alpha,F}$ = 230 Hz, C1α), 85.1 (d, $J_{2\beta,F}$ = 31.0 Hz, C2β), 83.9 (d, $J_{2\alpha,F}$ = 21.0 Hz, C2α), 83.3 (d, $J_{4,F}$ = 2.4 Hz, C4β), 80.1 (C3β), 80.0 (C3α), 78.4 (C4α), 73.5 (Ph*C*H_2 β), 73.5 (Ph*C*H_2 α), 72.9 (Ph*C*H_2 α), 72.8 (Ph*C*H_2 α), 72.5 (Ph*C*H_2 β), 72.2 (Ph*C*H_2 β), 69.3 (C5β), 68.7 (C5α); ^{19}F NMR (376 MHz; $CDCl_3$) -177.4 (ddd, $J_{1,F}$ = 64.6, $J_{2,F}$ = 6.8 Hz, $J_{4,F}$ = 6.8 Hz, Fβ), -133.0 (dd, $J_{1,F}$ = 64.8 Hz, $J_{2,F}$ = 18.9 Hz, Fα); HRMS (ESI$^+$) m/z calc. for $C_{26}H_{27}FO_4Na^+$ 445.1786 $[M+Na]^+$ found 445.1791 $[M+Na]^+$.

2,3,5-Tri-*O*-benzyl-α/β-D-arabinofuranosyl fluoride (18)[9]

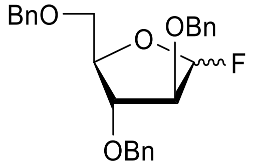

2,3,5-Tri-*O*-benzyl-α-D-arabinofuranose (1.0 g, 2.4 mmol) was dissolved into dry DCM (10 mL) and the solution cooled in an ice bath. DAST (390 µL, 2.9 mmol) was added in a single portion and the reaction mixture was allowed to warm to and was stirred at room temperature for 30 minutes. The reaction mixture was then cooled in an ice bath and MeOH (300 µL) was added to quench any unreacted DAST and the reaction mixture was again allowed to warm to and was stirred at room temperature for 30 minutes. The reaction mixture was then diluted with DCM (20 mL) and washed with saturated aqueous $NaHCO_3$ solution (2 × 10 mL). The aqueous extract was washed with DCM (3 × 5 mL) and the organic layers were combined, dried over $MgSO_4$, filtered and dried *in vacuo* to give the title compounds as a 5.3:1 mixture α/β mixture of anomers **18** (950 mg, 94%). The crude mixture was judged by NMR as clean enough to be used in the next step without further purification; R_f 0.73 (α) 0.66 (β) (hexane/ethyl acetate 8:2); ^{1}H NMR (400 MHz, $CDCl_3$): 7.37-7.25 (m, 15H, Ar), 5.78 (d, $J_{1,F}$ = 61.5 Hz, 1H, H-1α), 5.62 (dd, $J_{1,F}$ = 64.9 Hz, $J_{1,2}$ = 3.5 Hz, H-1β), 4.56-4.46 (m, 7H, 3 × PhC*H*$_2$ & H-4α), 4.16 (dd, $J_{2,F}$ = 9.3 Hz, $J_{2,3}$ = 2.0 Hz, 1H, H-2α), 3.97 (dd, $J_{2,3}$ = 2.0 Hz, $J_{3,4}$ = 5.2 Hz, 1H, H-3α), 3.64-3.57 (m, 2H, H-5α & H-5α'); ^{13}C NMR (100.6 MHz, $CDCl_3$): 137.9, 137.5, 137.0 (3 × Ar), 128.6, 128.5, 128.4, 128.1, 127.9, 127.8, 127.8, 127.8, 127.7, 113.6 (d, $J_{C1,F}$ = 224.7 Hz, C1α), 108.4 (d, $J_{C1,F}$ = 232.3 Hz, C1β), 86.9 (d, $J_{C2,F}$ = 33.9 Hz, C2α), 84.1 (C4α), 82.5 (C3α), 73.5, 72.1, 72.1 (3 × Ph*C*H_2), 69.4 (C5α); ^{19}F NMR (376 MHz; $CDCl_3$) -120.6 (dd, $J_{1,F}$ = 61.4 Hz, $J_{2,F}$ = 9.0 Hz, α-fluoride), 126.6 (ddd, $J_{1,F}$ = 64.6 Hz, $J_{2,F}$ = 20.6 Hz, $J_{3,F}$ = 6.1 Hz, β-

fluoride); HRMS (ESI$^+$) m/z calc. for $C_{26}H_{27}FO_4Na$ 445.1786 [M+Na]$^+$ found 445.1784 [M+Na]$^+$. The ^1H, ^{13}C and ^{19}F NMR diagnostic signals were in agreement with literature values. [9]

2',3',5'-Tri-*O*-benzyl-β-D-ribofuranosyl (*N*-phenyl)-2,2,2-trifluoroacetimidate (25) [10]

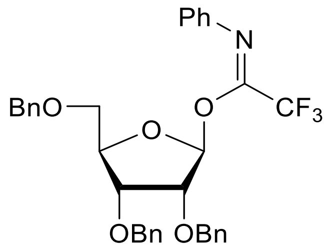

2,3,5-Tri-*O*-benzyl-β-D-ribofuranose (840 mg, 2.0 mmol), 2,2,2-trifluoro-*N*-phenylacetimidoyl chloride (600 µL, 4.0 mmol), caesium carbonate (720 mg, 2.2 mmol) and water (100 µL) were dissolved into acetone and stirred for 3 hours at room temperature. The reaction mixture was then filtered through Celite® and the solvent was removed *in vacuo* to give a crude syrup. The crude mixture was purified by FCC to give **25** (800 mg, 59%) as an off white powder; R_f 0.61 (hexane/EtOAc 8:2); ^1H NMR (400 MHz, $CDCl_3$): 7.54-7.52 (m, Ar, 3H), 7.32-7.25 (m, Ar,) 7.17 (t, J = 7.5, Ar, 1H), 7.08 (t, J = 7.5, Ar, 1H), 6.79 (d, J = 7.8, 1H), 6.32 (bs, 1H, H-1), 4.66-4.43 (m, 7H, 3 × CH_2Ph & H-4), 4.17-4.06 (m, 2H, H-3 & H-4), 3.70 (dd, $J_{4,5}$ = 2.8 Hz, $^2J_{5,5'}$ = 10.9 Hz, 1H, H-5), 3.59 (dd, $J_{4,5'}$ = 5.2 Hz, $^2J_{5,5'}$ = 10.9 Hz, 1H, H-5'); ^{13}C NMR (100.6 MHz, $CDCl_3$): 143.8 (C=N), 138.1, 137.5, 137.4, 135.4, 129.5, 128.8, 128.5, 128.5, 128.4, 128.2, 128.1, 128.0, 128.0, 127.7, 126.3, 124.4, 119.7 (CF_3), 102.5 (C1), 82.3 (C4), 78.7 (C2), 77.4 (C3) , 73.4, 72.8, 72.4 (3 × CH$_2$Ph), 70.2 (C5); δ_F (376 MHz; $CDCl_3$) -75.6 (CF_3); LRMS (ESI$^+$) m/z calc. for $C_{34}H_{32}F_3NO_5Na^+$ 614.2 [M+Na]$^+$ found 613.6 [M+Na]$^+$. The NMR data were in accordance with the literature.[10]

NMR Spectra

1,3-Bis(benzyloxy)propan-2-yl 2,3,5,6-tetra-*O*-benzoyl-β-D-galactofuranoside (10)

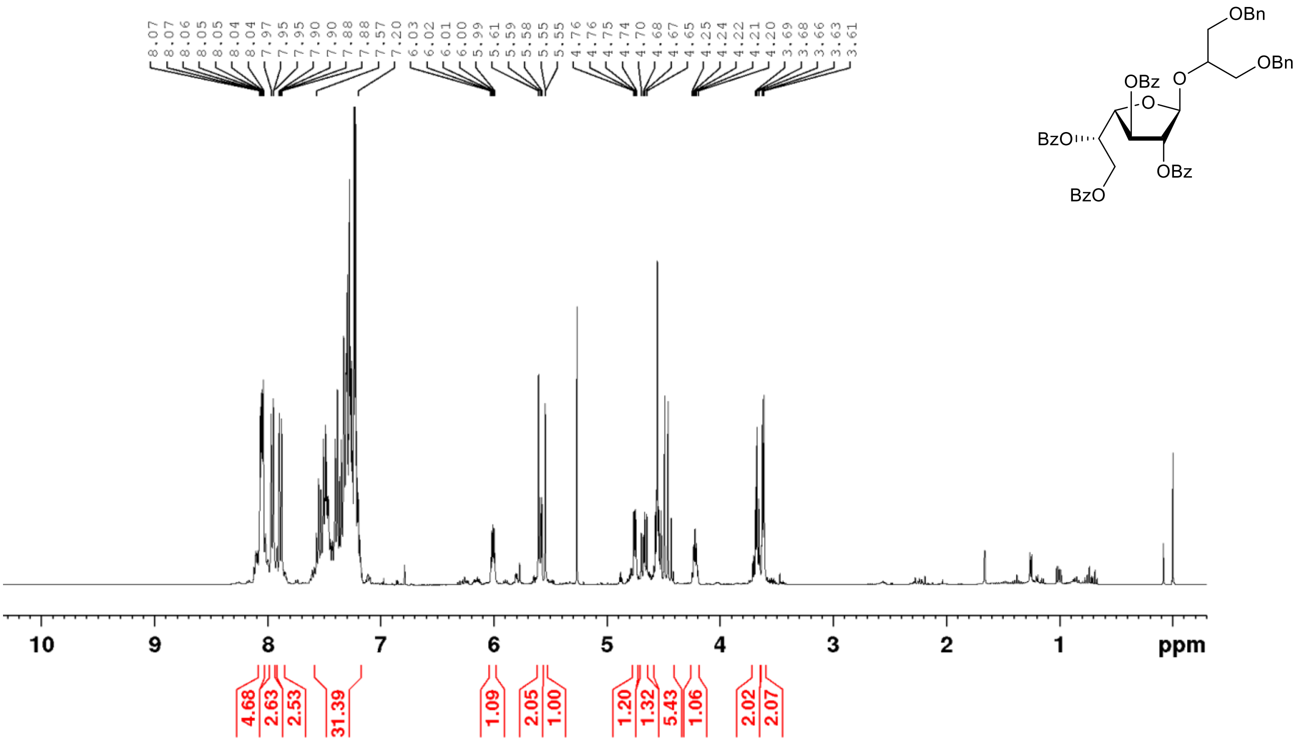

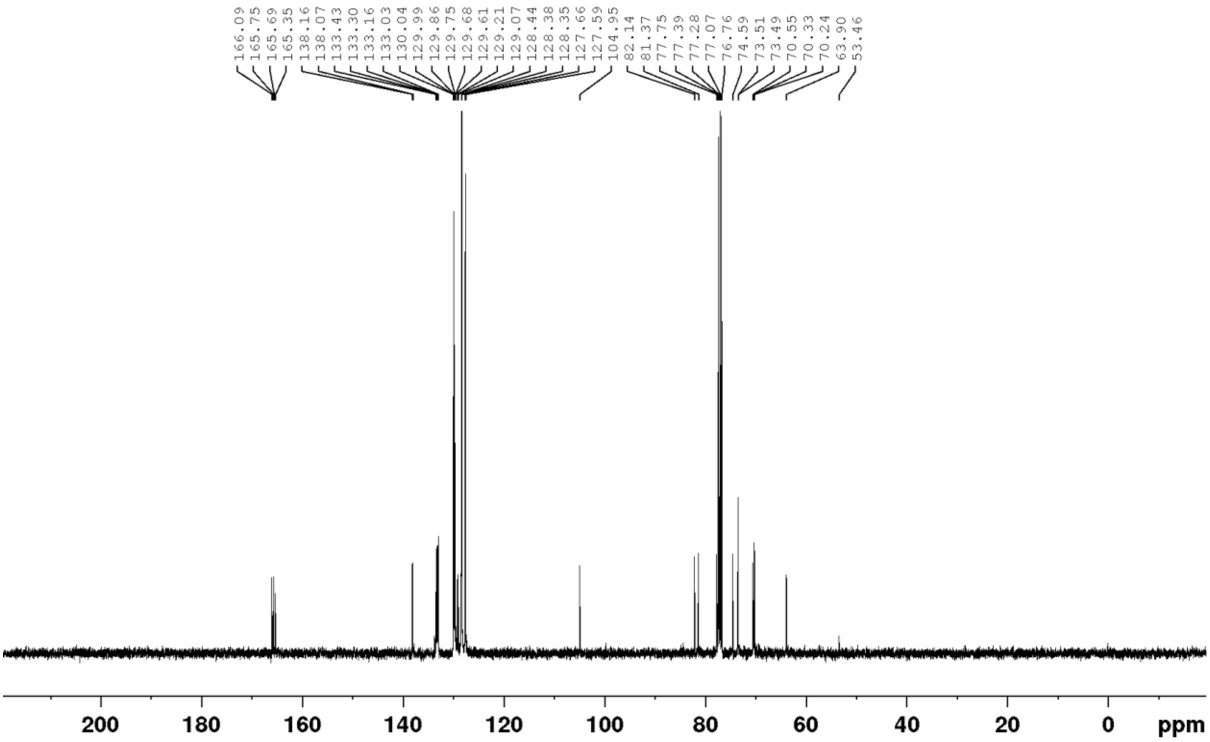

1,3-Dihydroxypropan-2-yl 2,3,5,6-tetra-O-benzoyl-β-ᴅ-galactofuranoside (11)

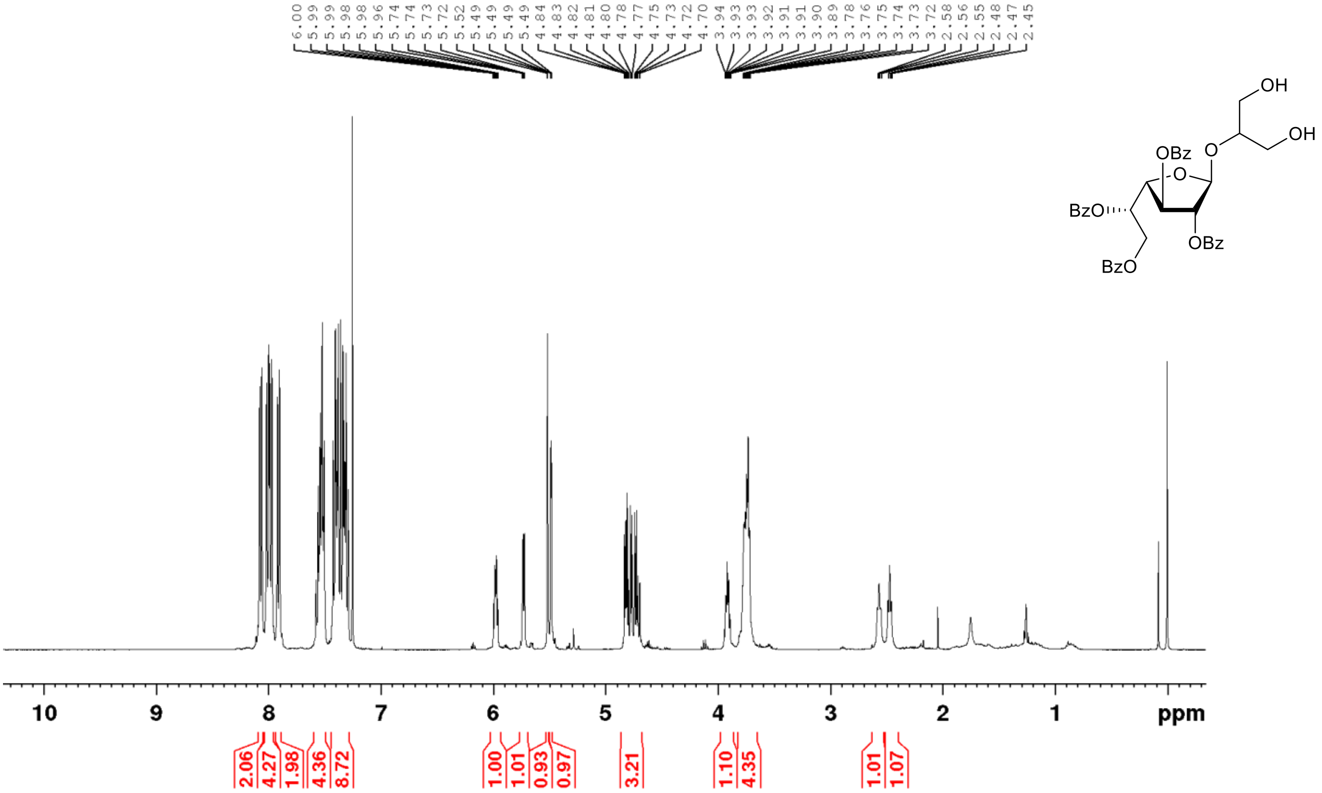

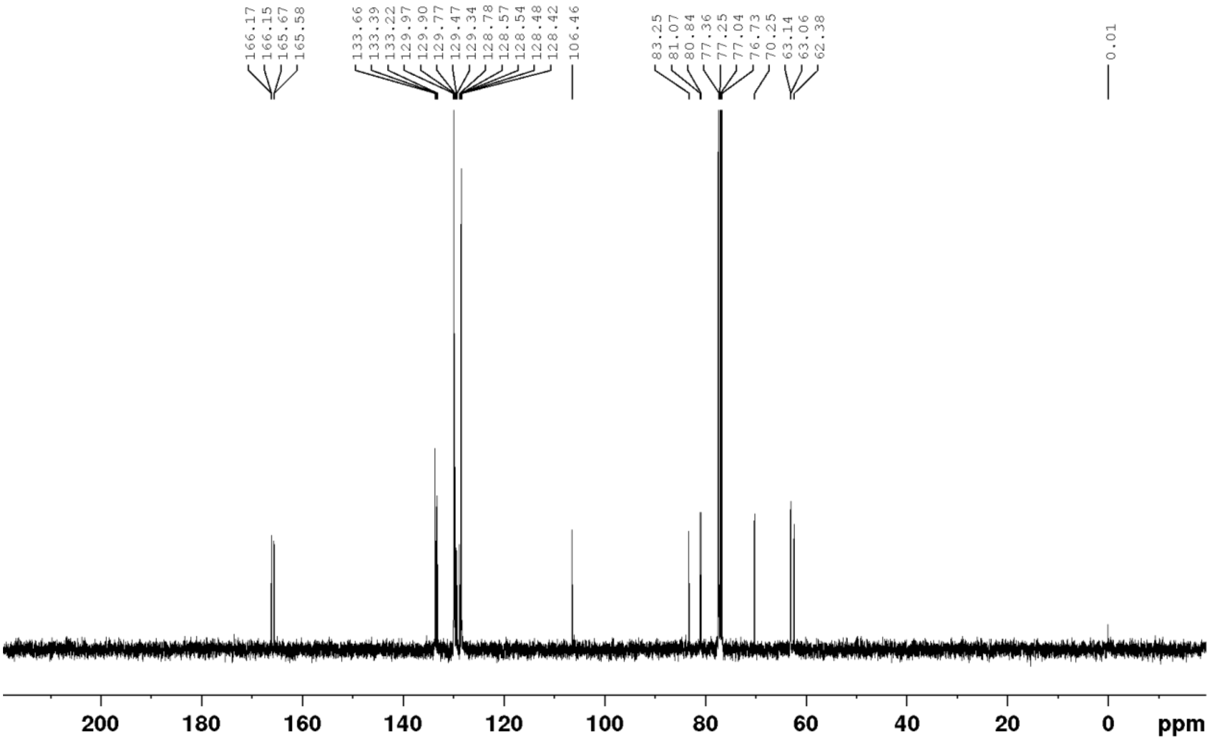

1,3-Dihydroxypropan-2-yl β-D-galactofuranoside (1)

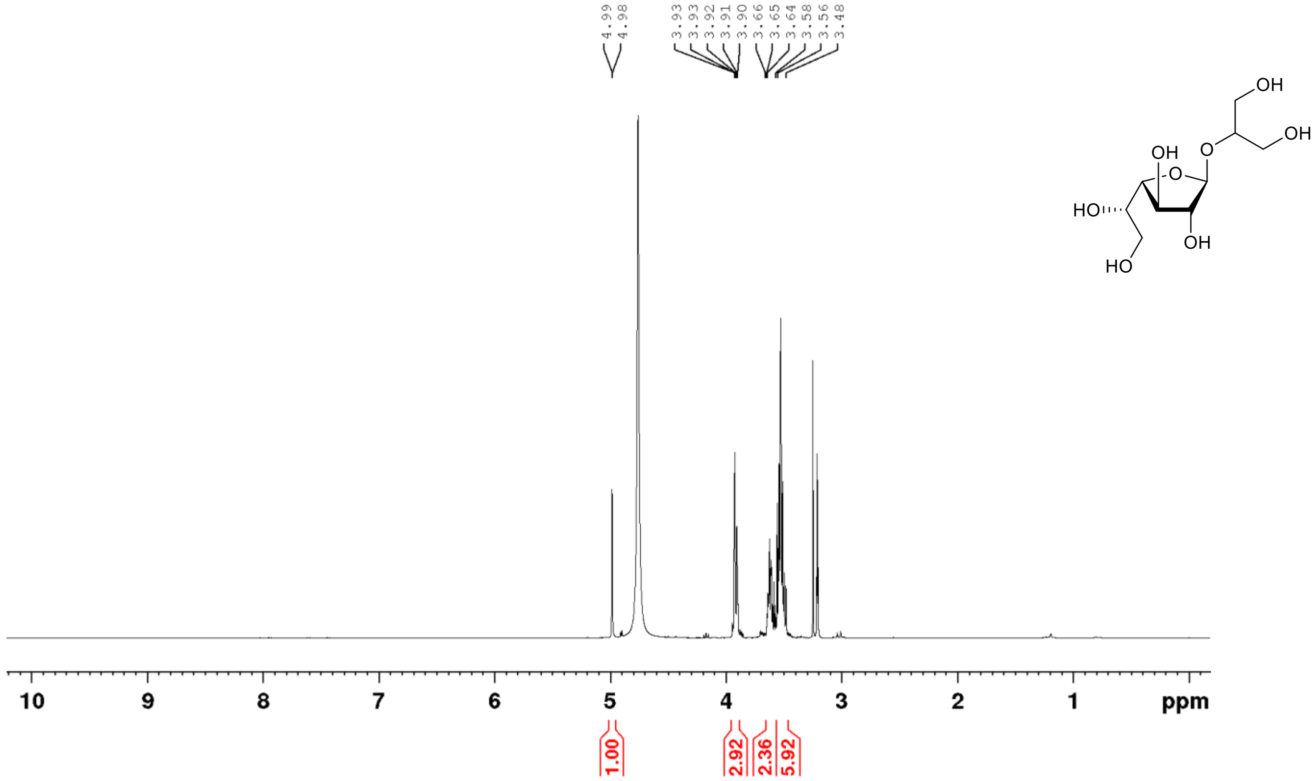

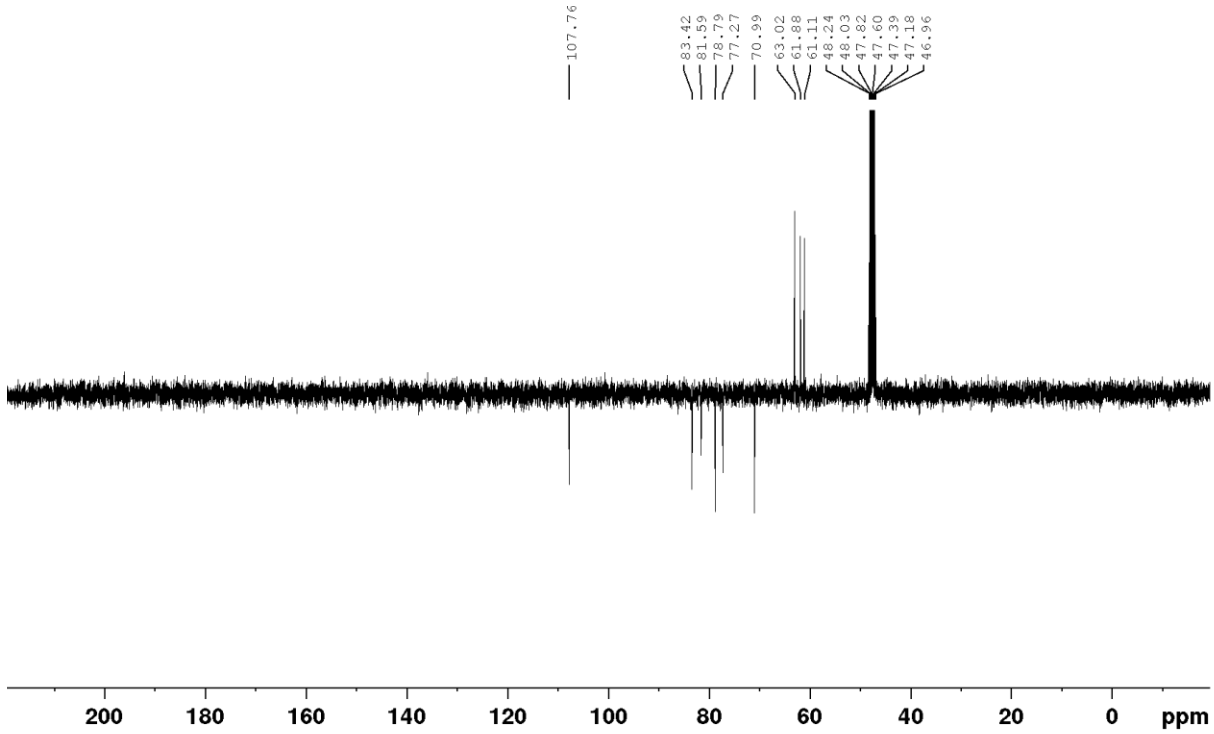

1,3-Bis(benzyloxy)propan-2-yl 2,3,4-tri-*O*-benzoyl-α-L-arabinopyranoside (14)

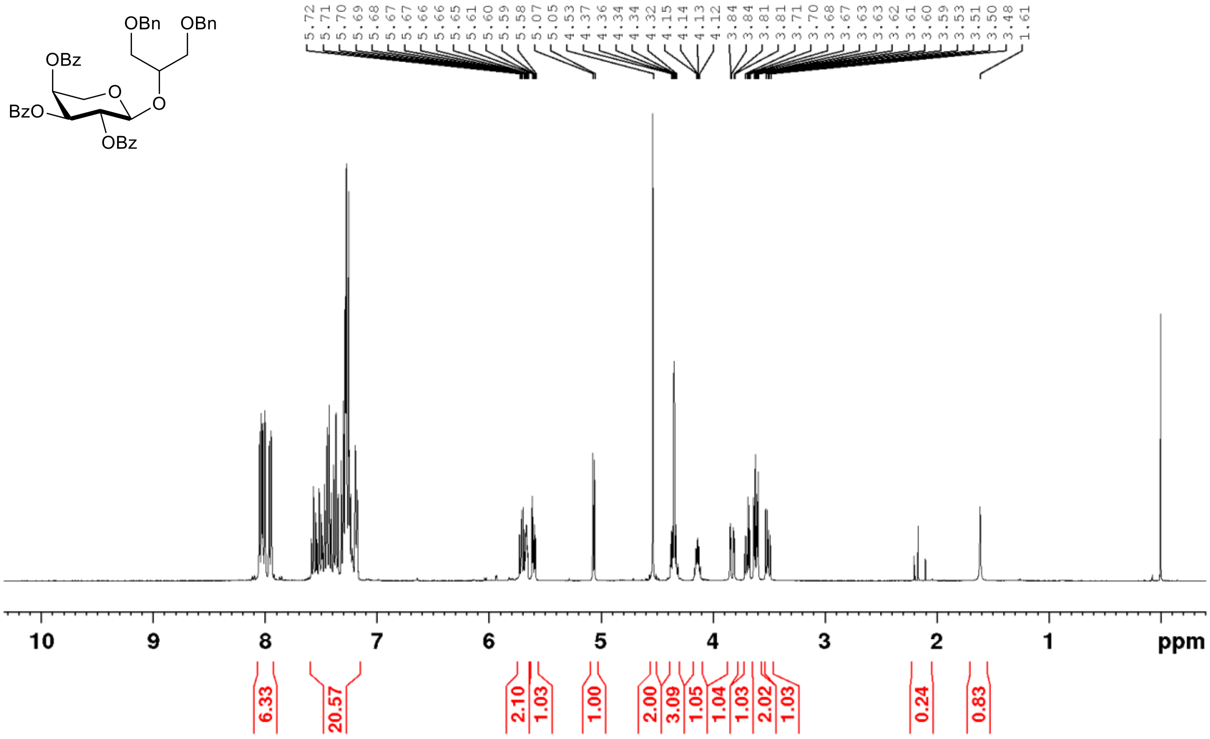

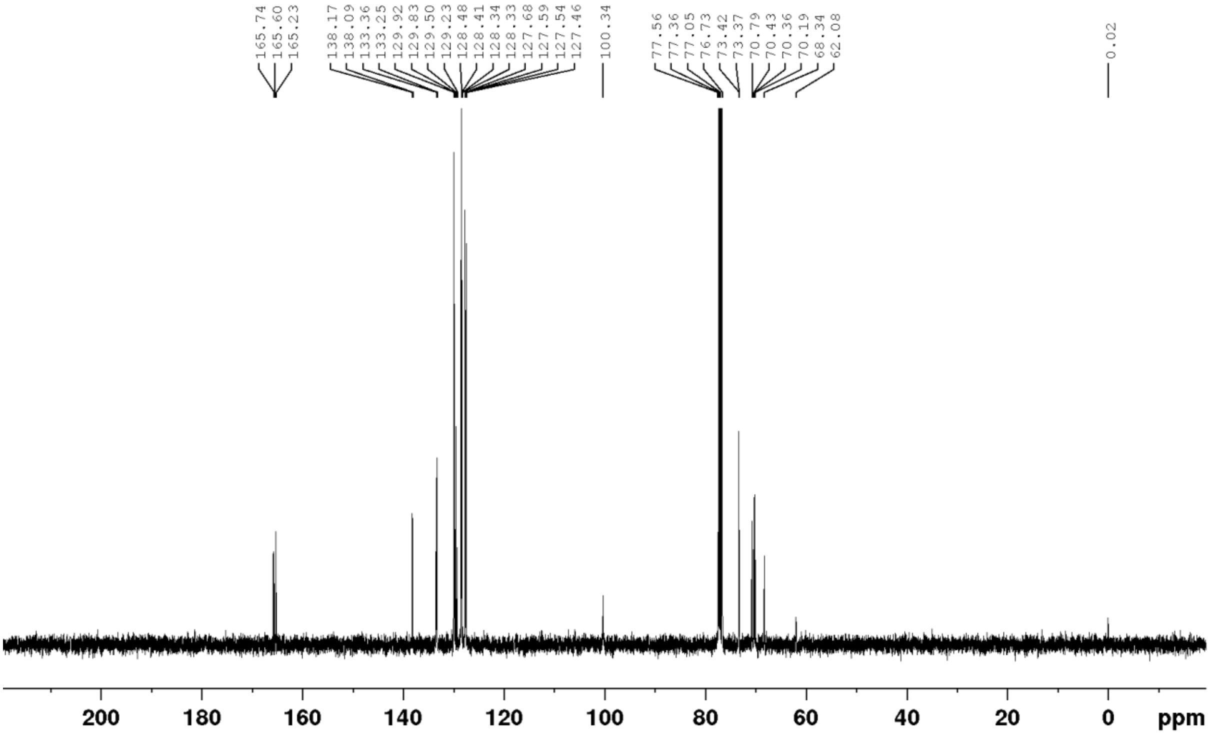

1,3-Dihydroxypropan-2-yl 2,3,4-tri-*O*-benzoyl-α-L-arabinopyranoside (15)

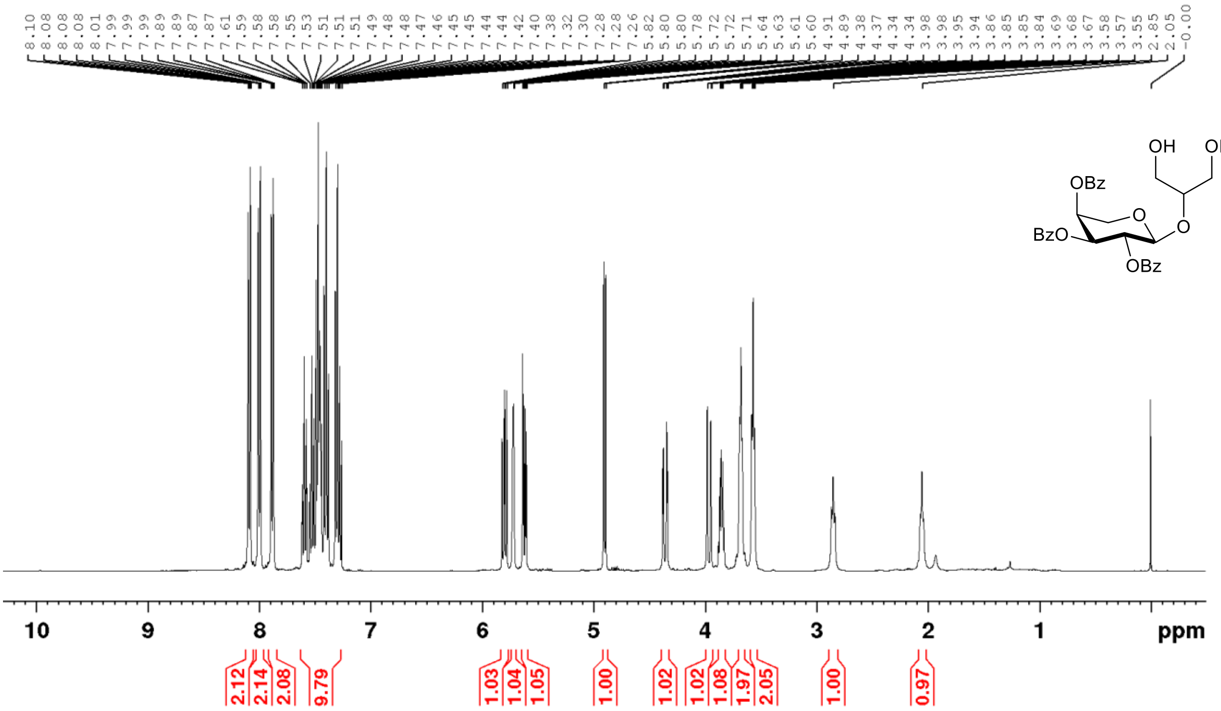

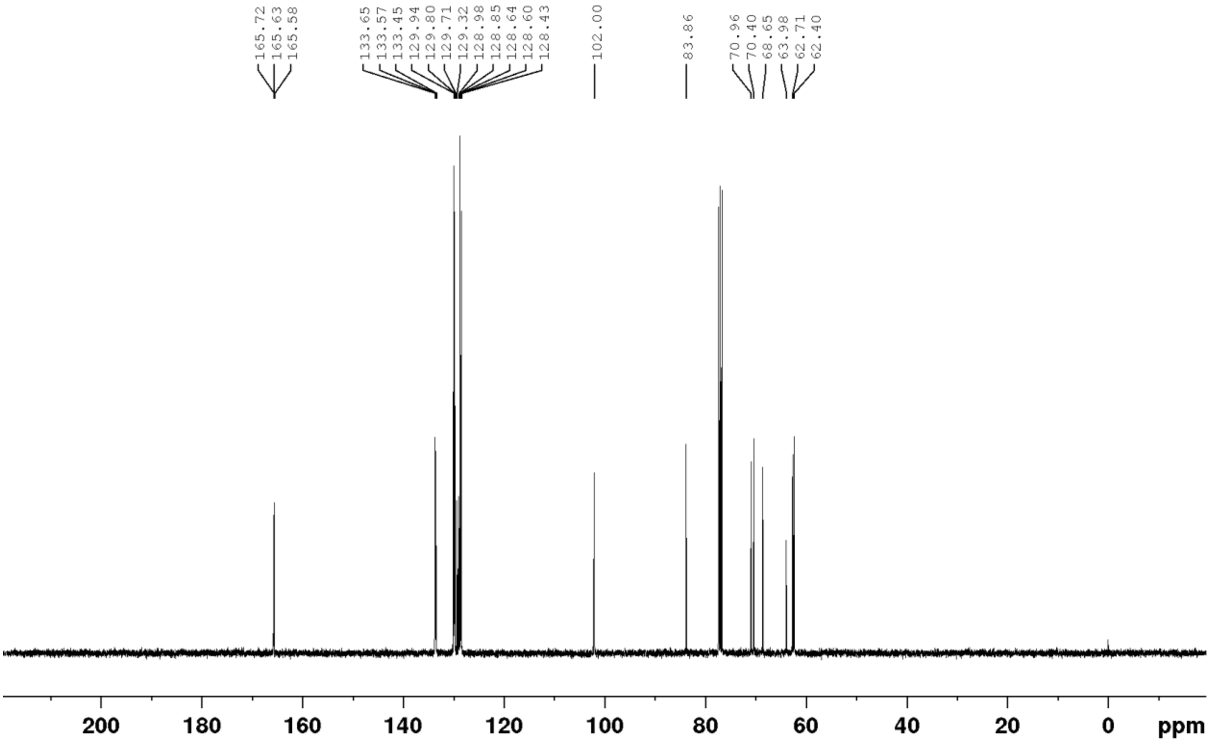

1,3-Dihydroxypropan-2-yl α-ʟ-arabinopyranoside (2)

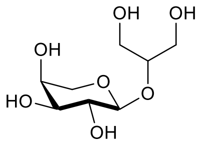

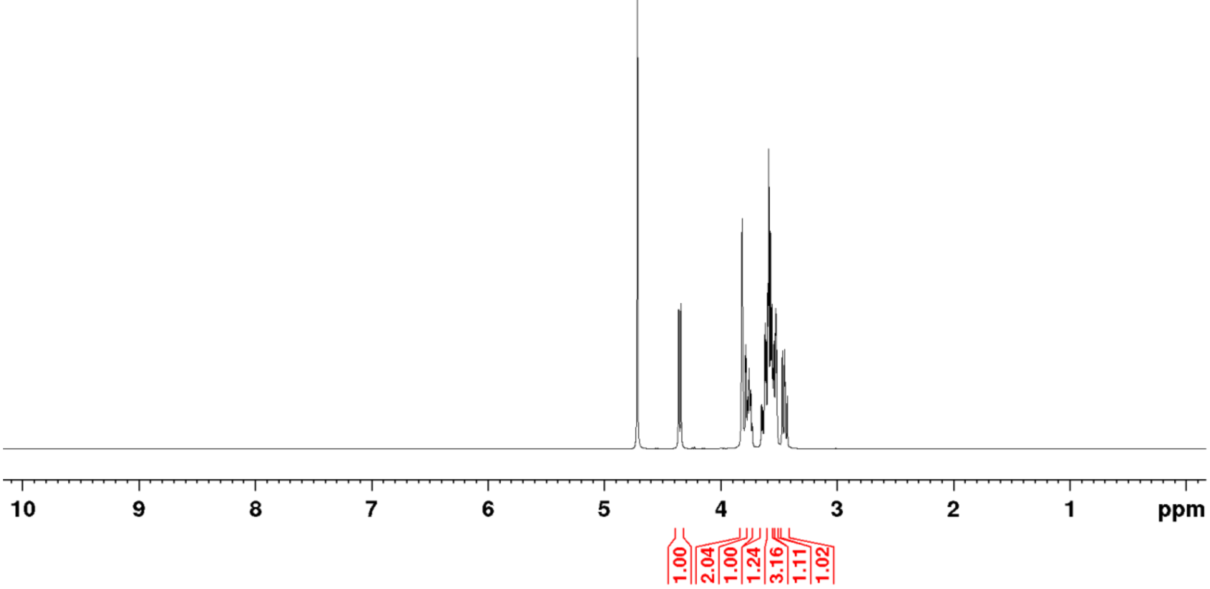

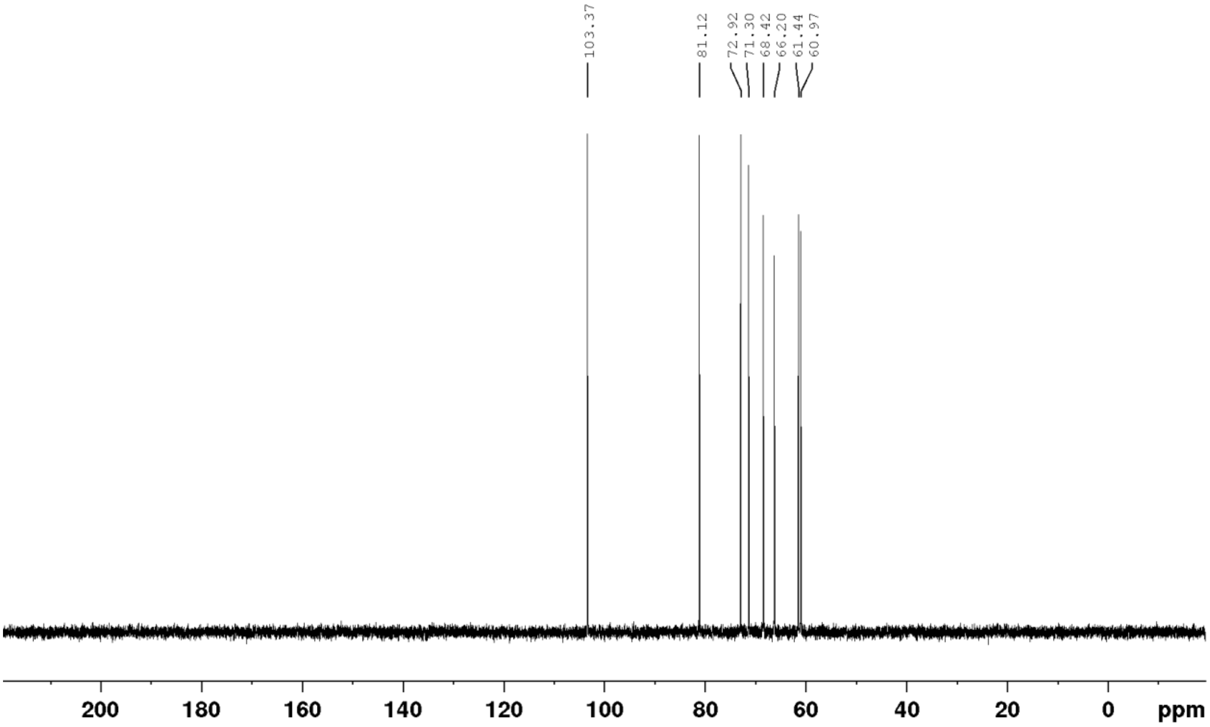

1,3-Bis(benzyloxy)propan-2-yl 2,3,5-tri-*O*-benzyl-α-D-ribofuranoside (19a)

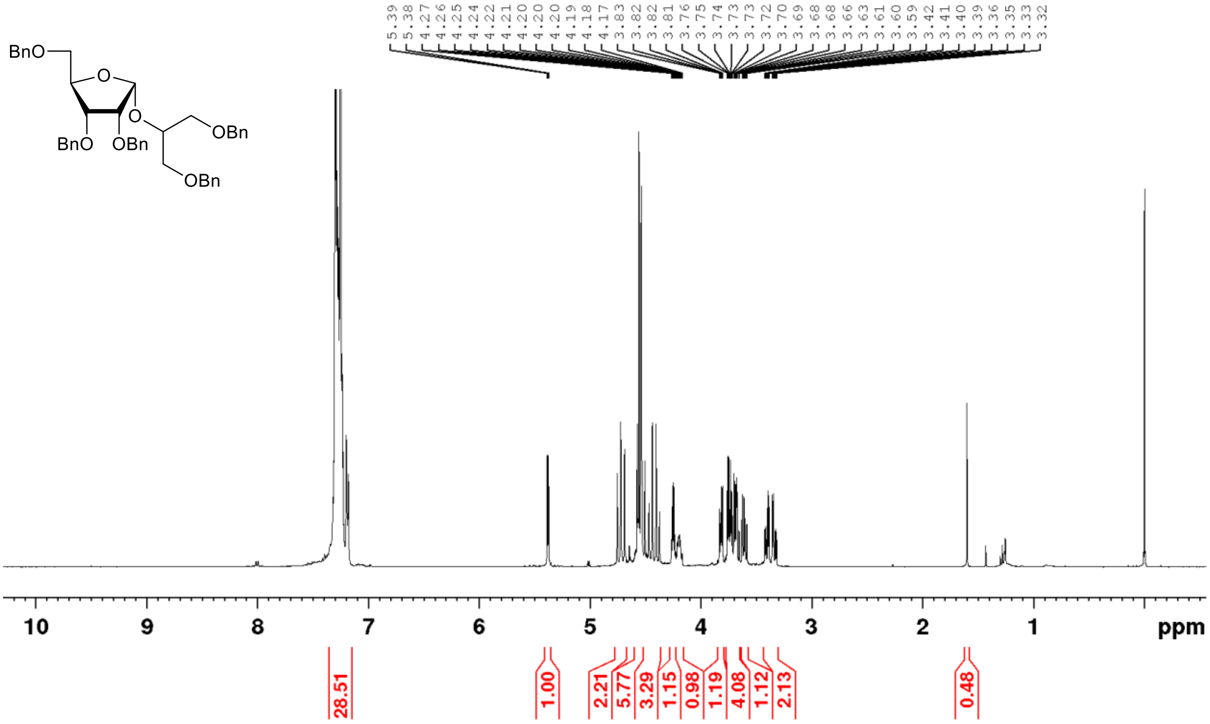

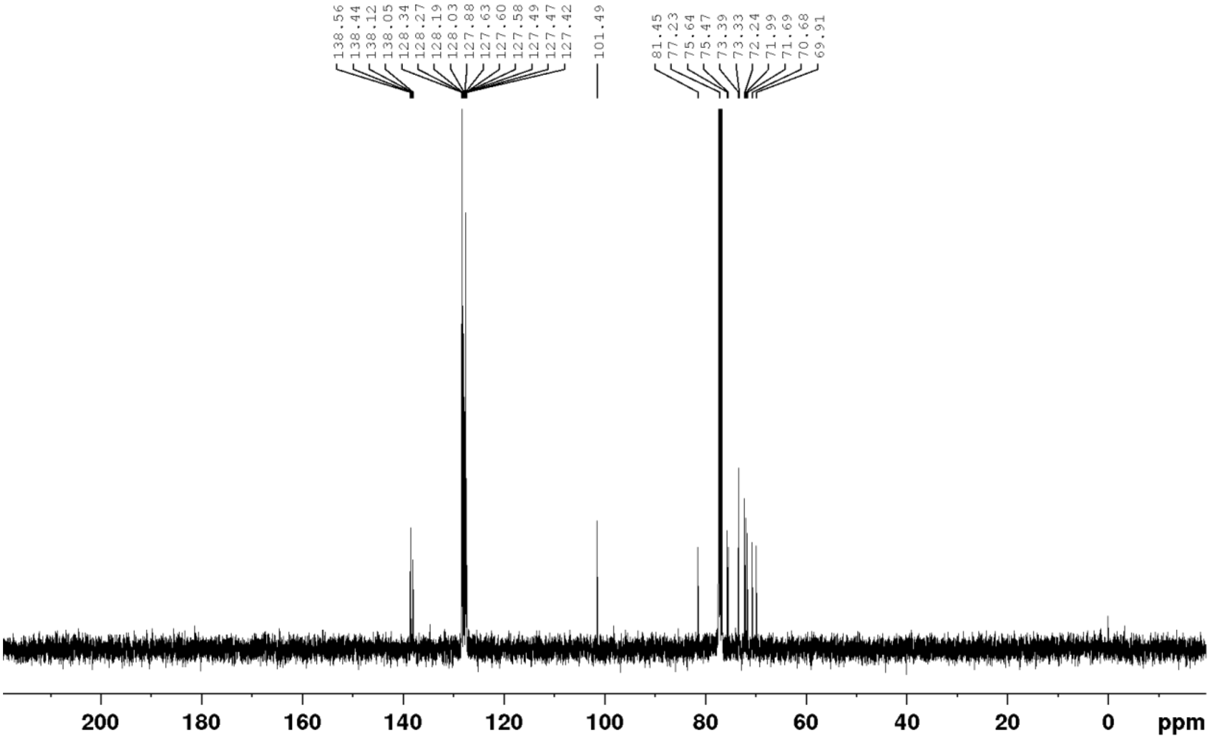

1,3-Bis(benzyloxy)propan-2-yl 2,3,5-tri-*O*-benzyl-β-D-ribofuranoside (19b)

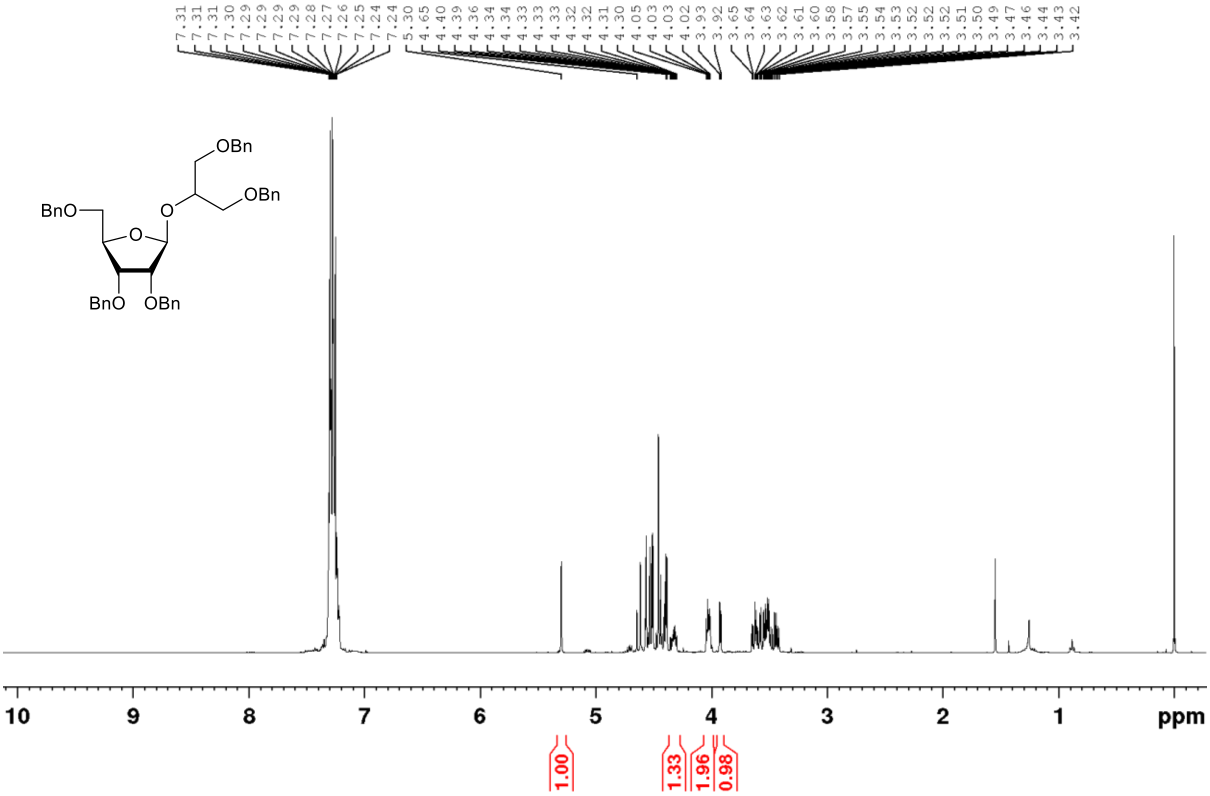

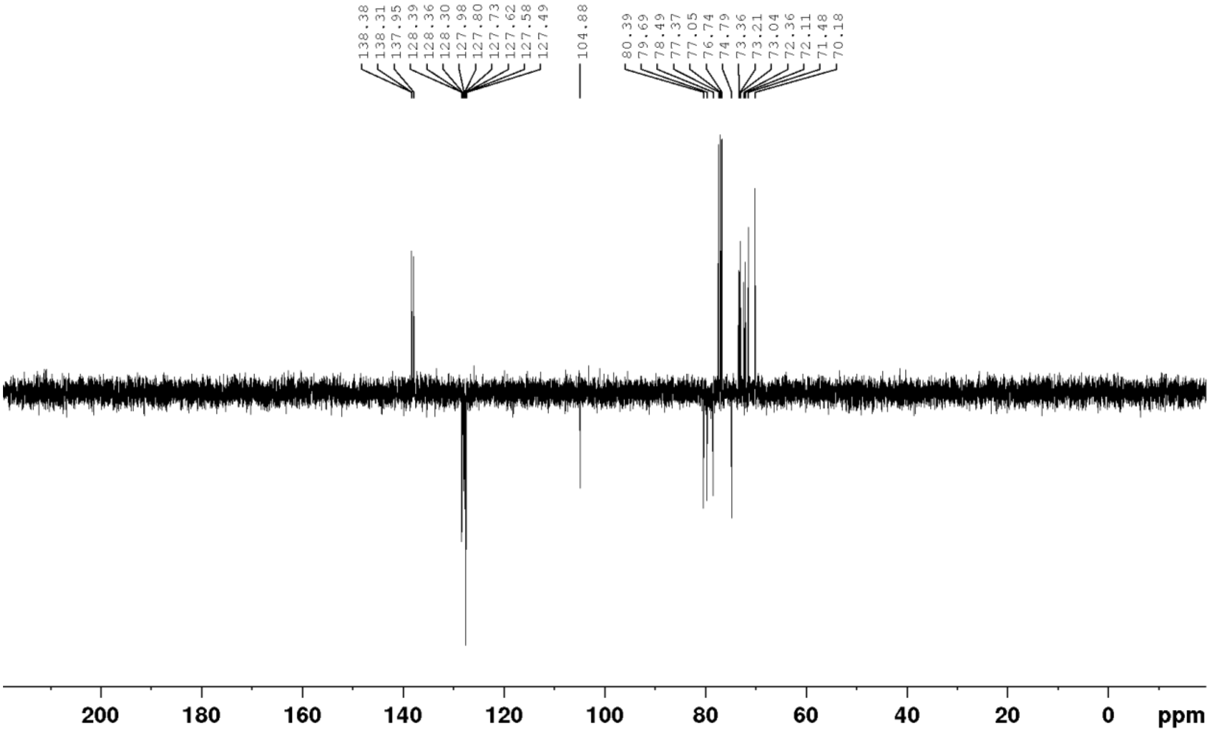

1,3-Dihydroxypropan-2-yl α-ᴅ-ribofuranoside (3)

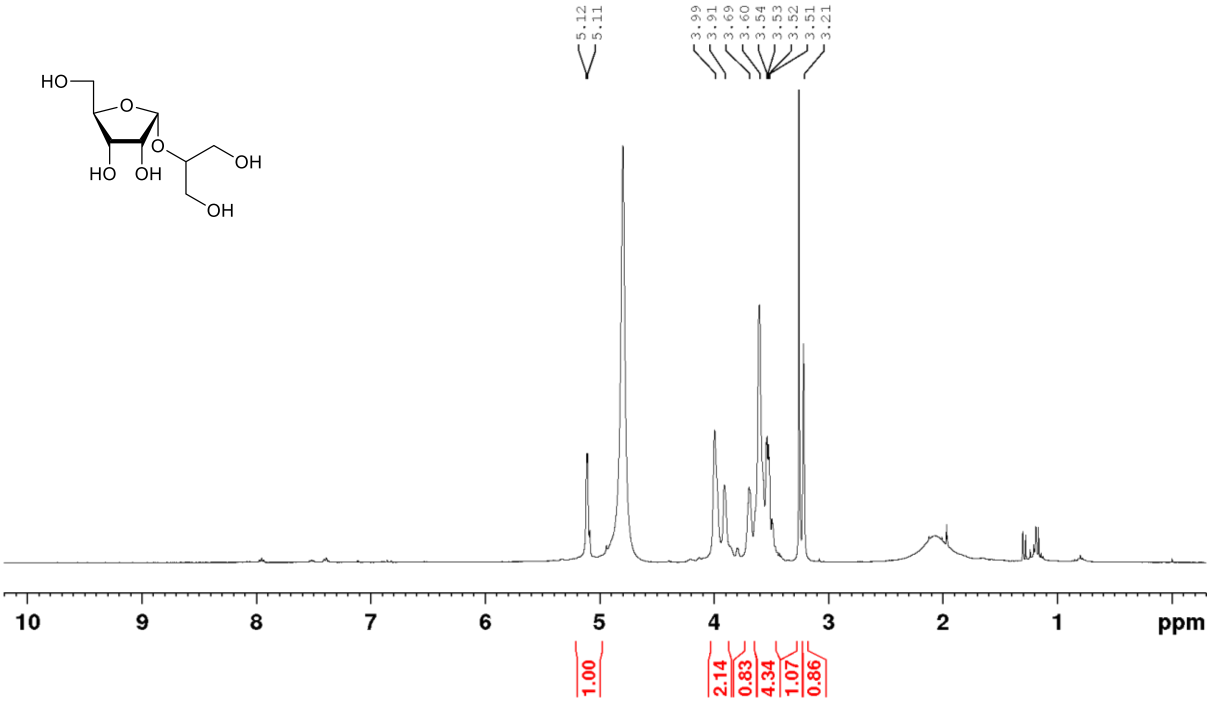

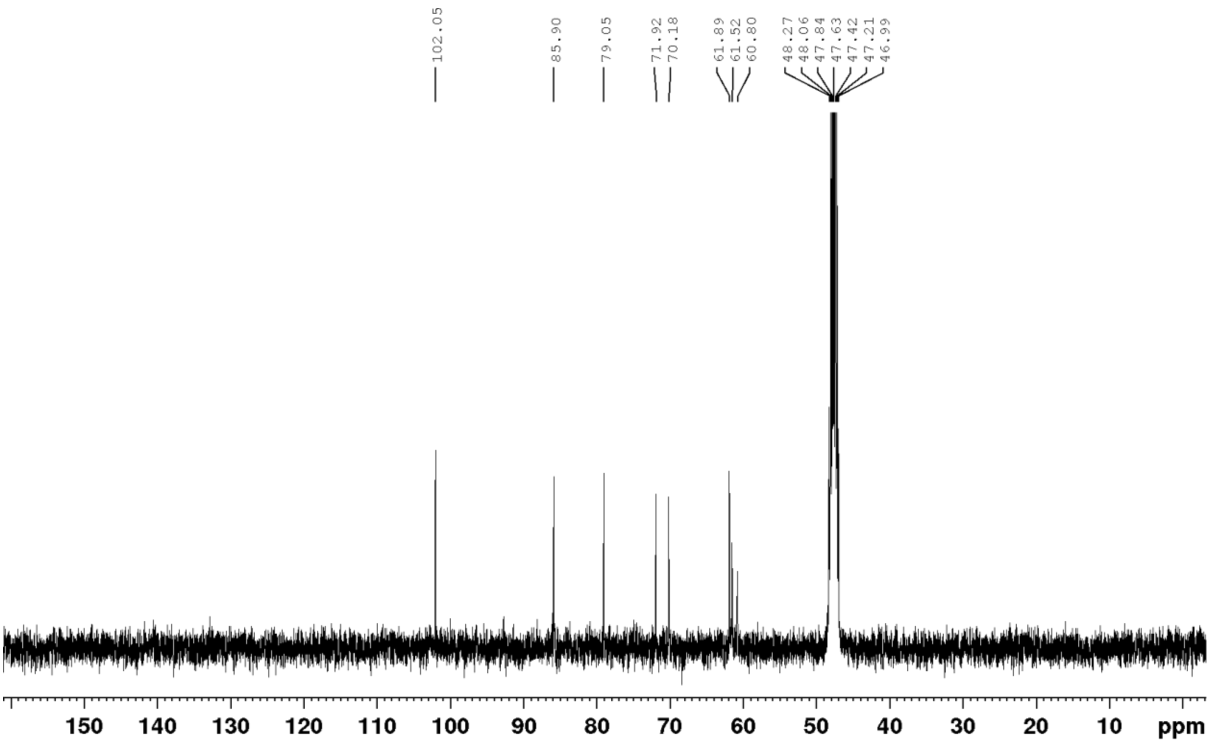

1,3-Bis(benzyloxy)propan-2-yl 2,3,5-tri-*O*-benzyl-α-L-xylofuranoside (20a)

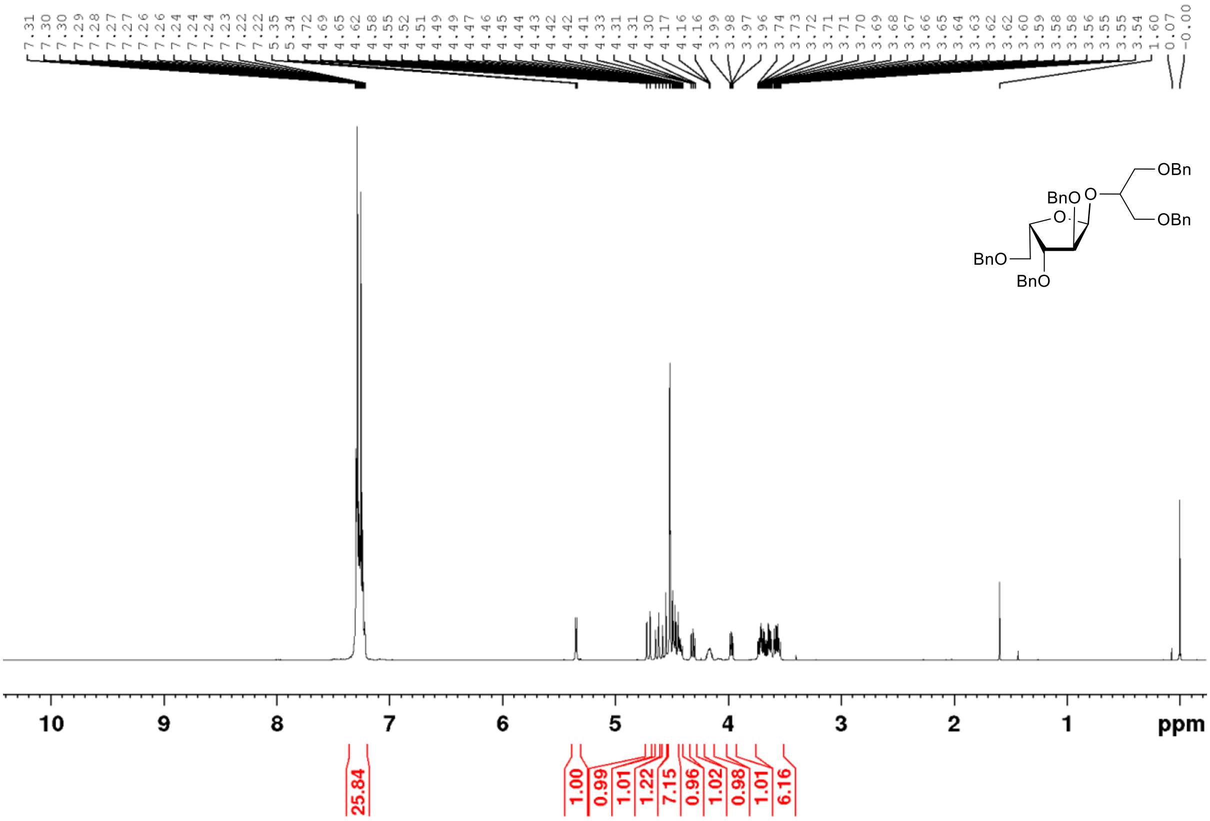

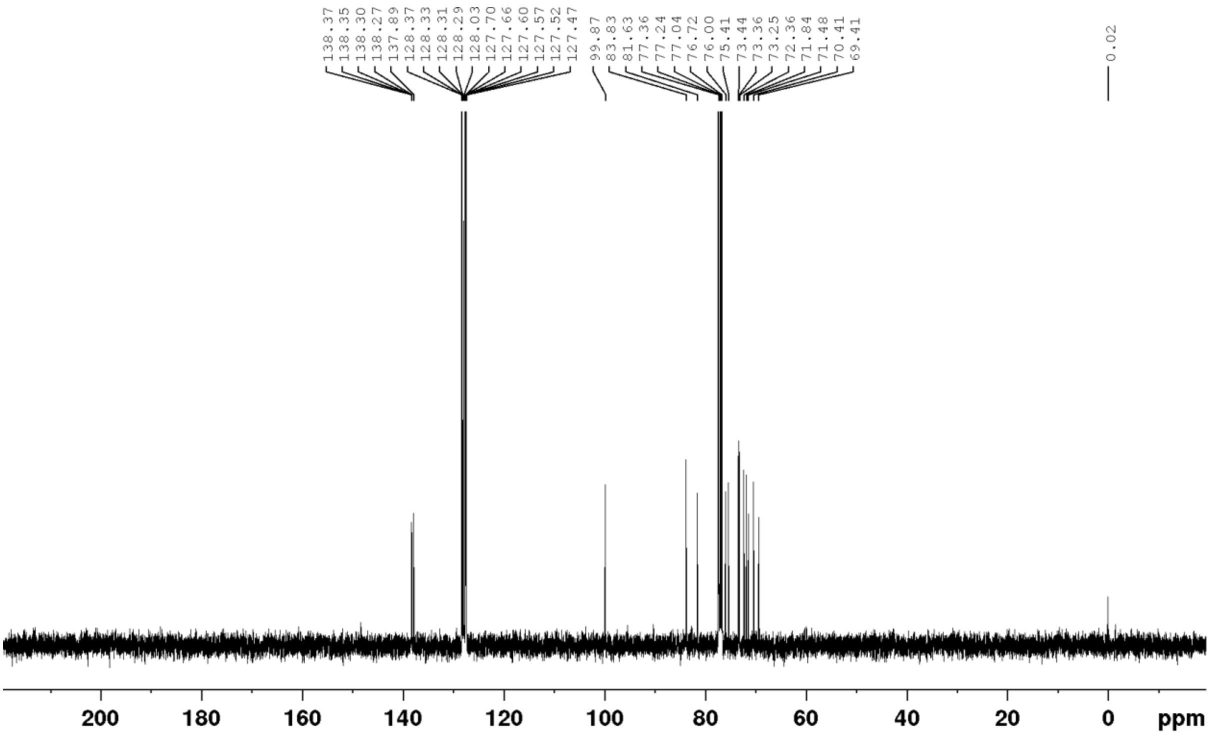

1,3-Bis(benzyloxy)propan-2-yl 2,3,5-tri-*O*-benzyl-β-L-xylofuranoside (20b)

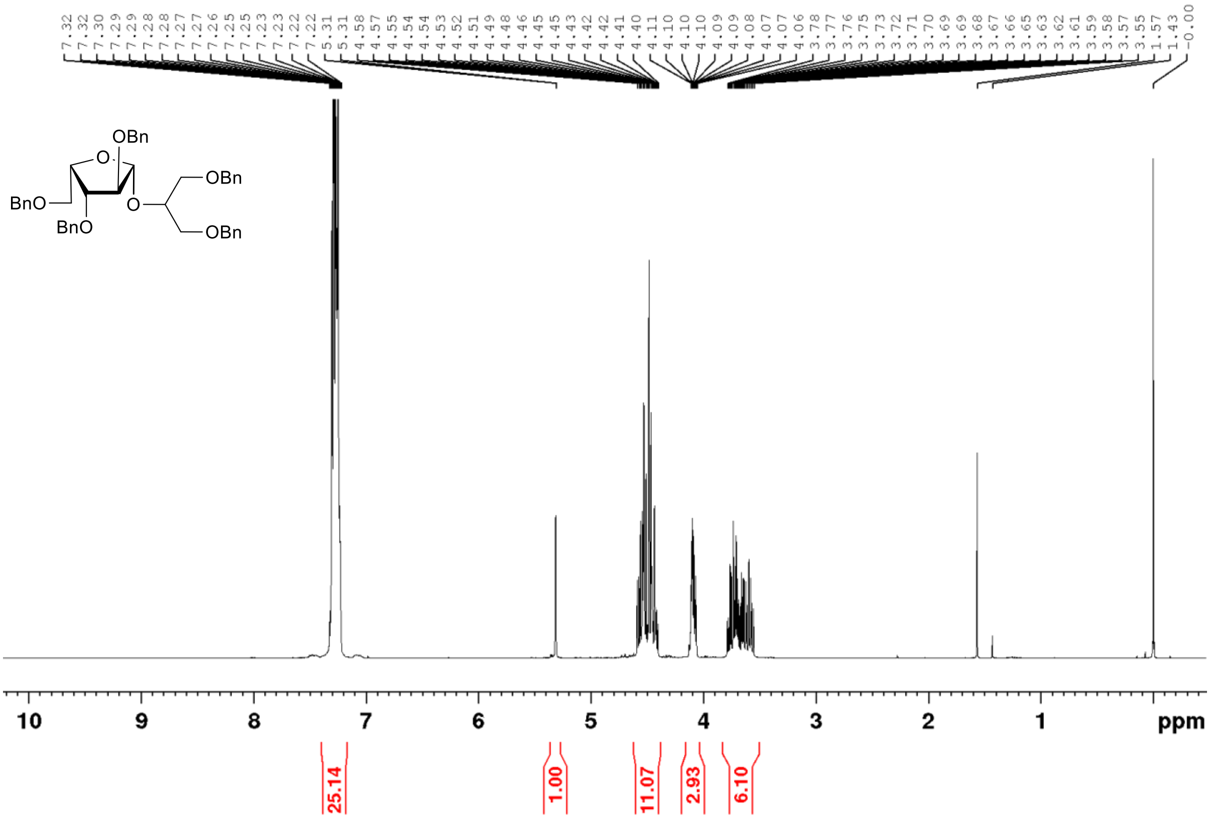

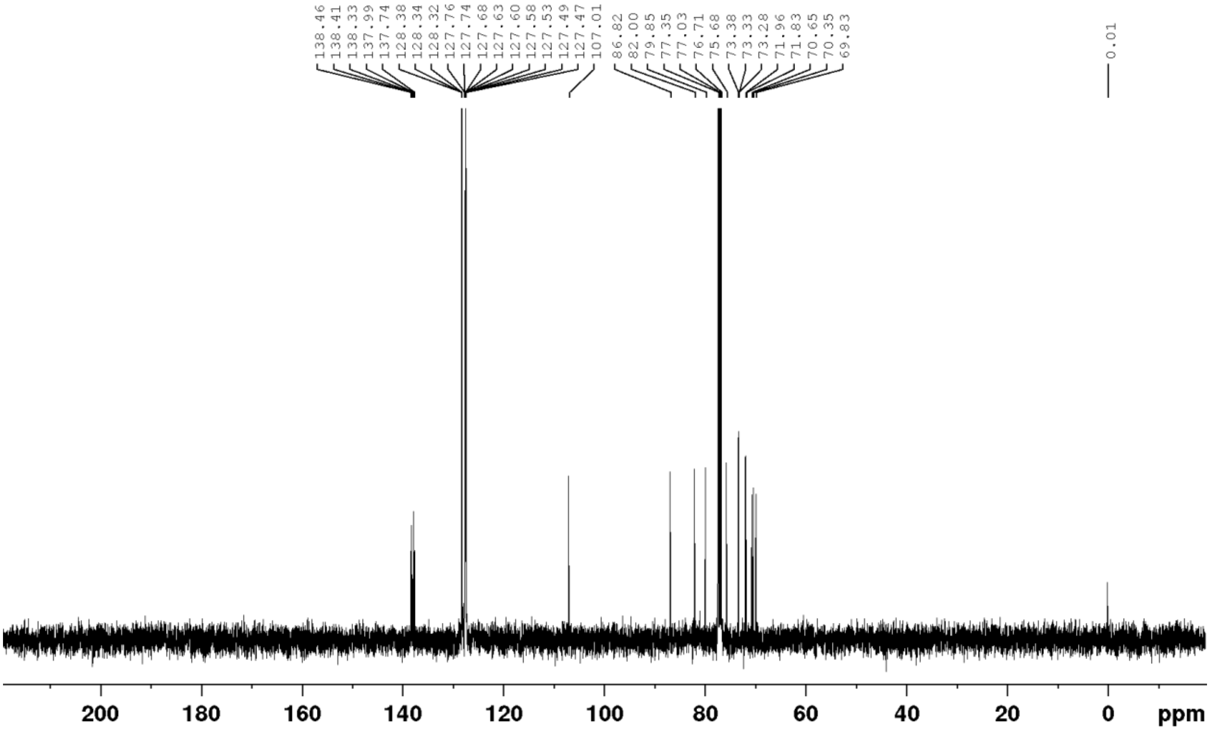

1,3-Dihydroxypropan-2-yl α-ʟ-xylofuranoside (4)

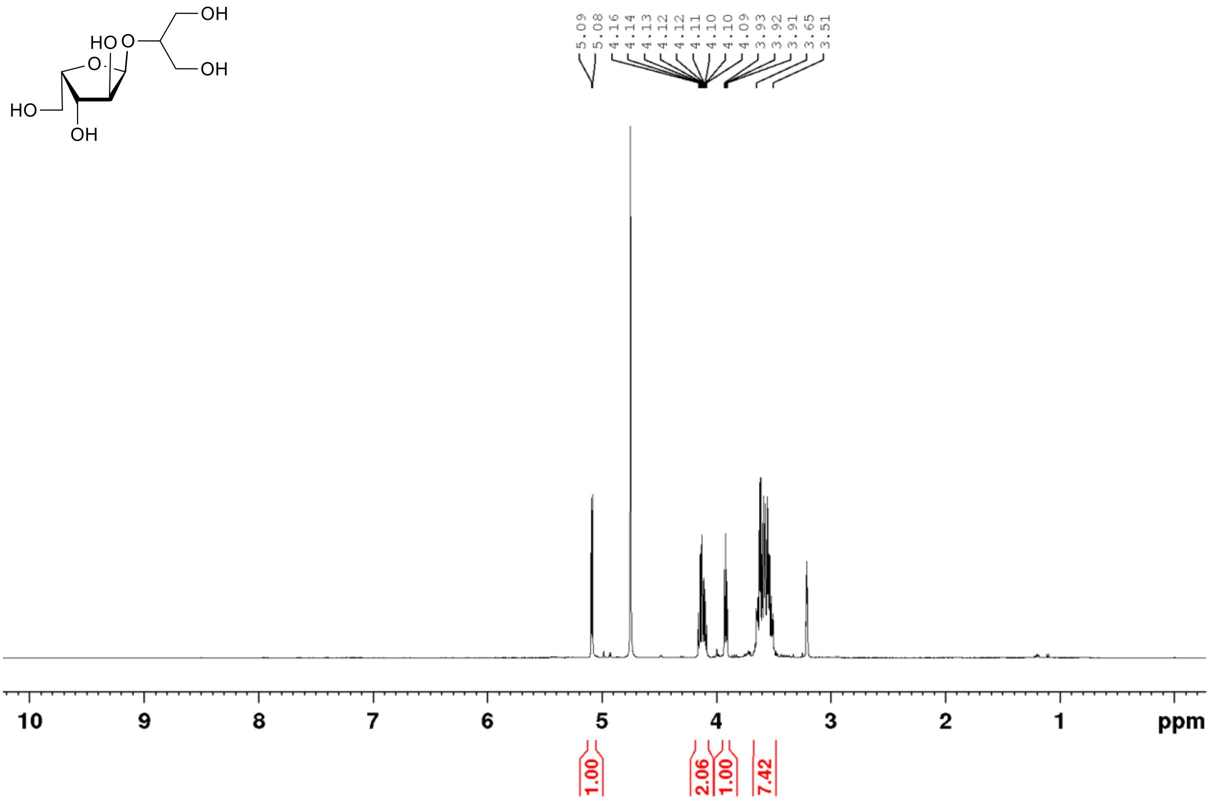

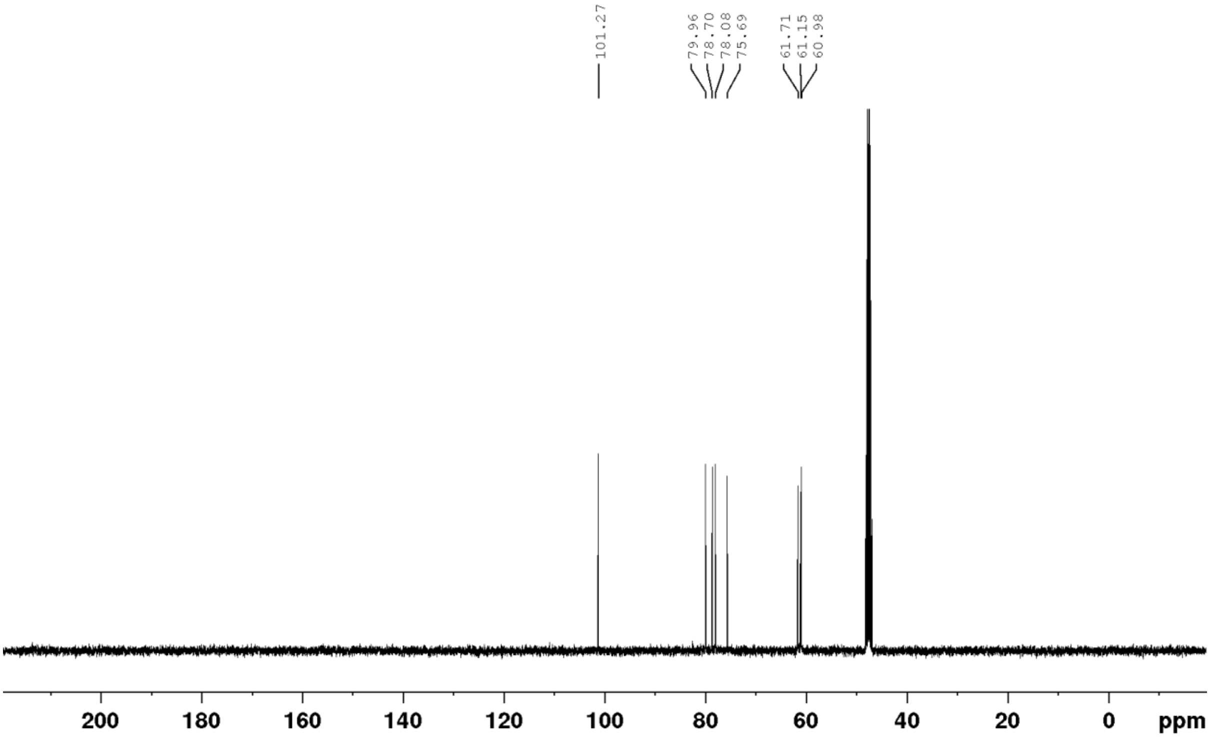

1,3-Bis(benzyloxy)propan-2-yl 2,3,5-tri-*O*-benzyl-β-D-arabinofuranoside (21a)

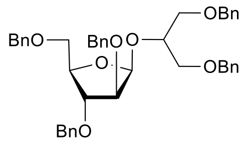

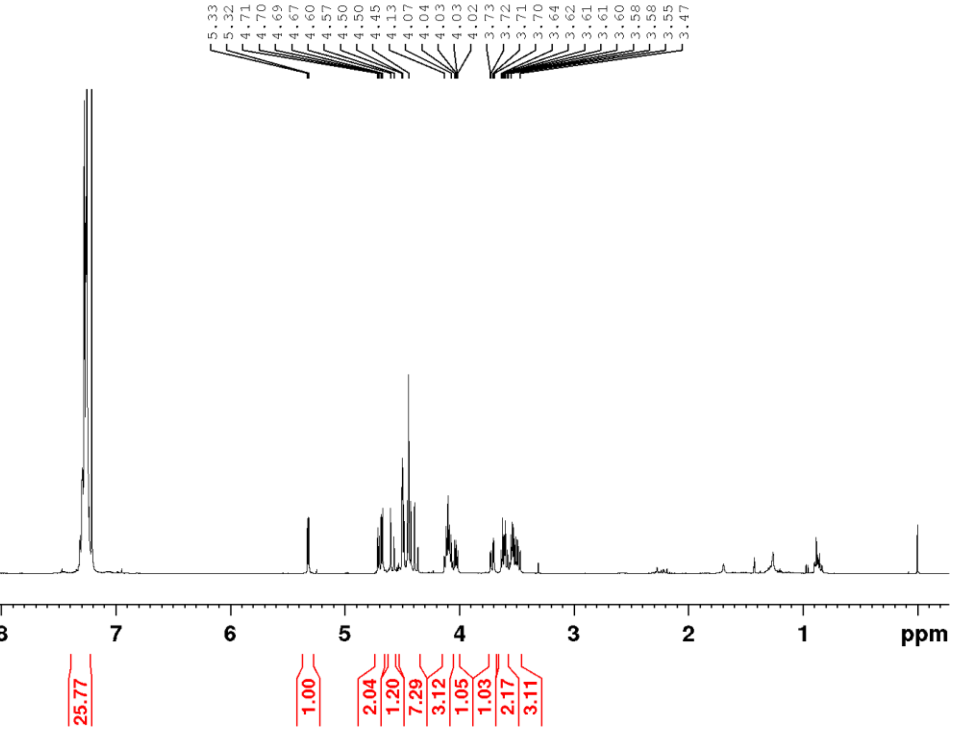

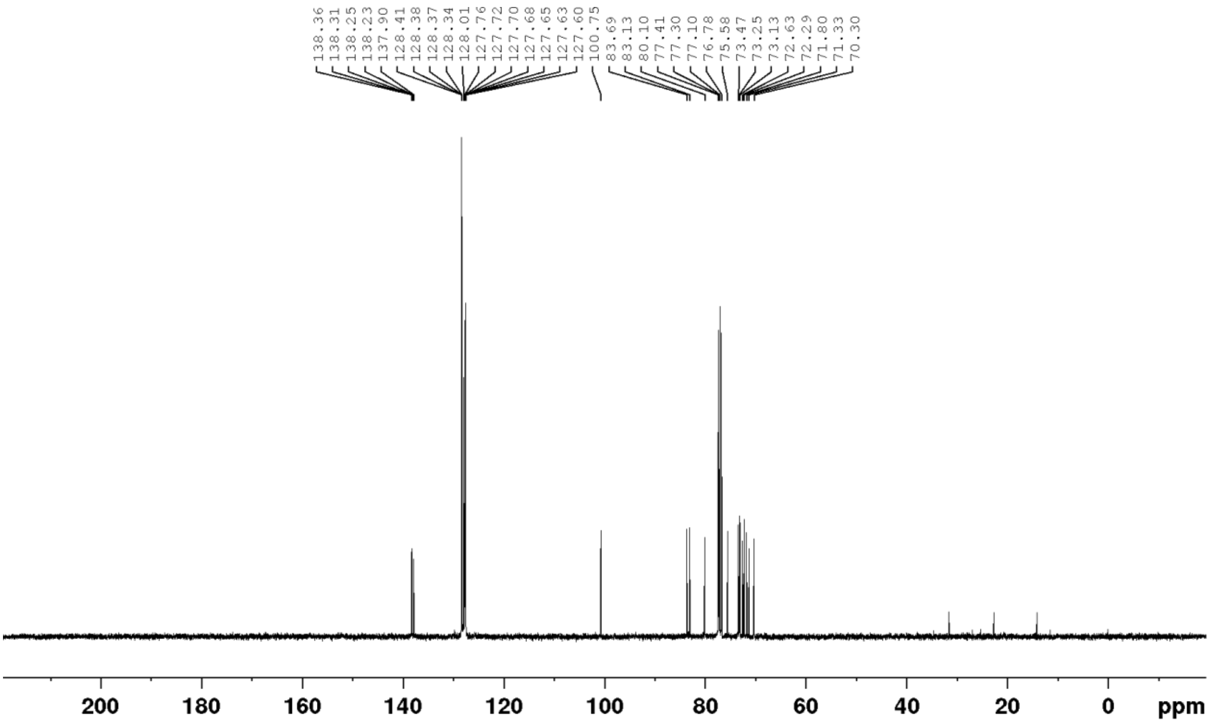

1,3-Bis(benzyloxy)propan-2-yl 2,3,5-tri-*O*-benzyl-α-D-arabinofuranoside (21b)

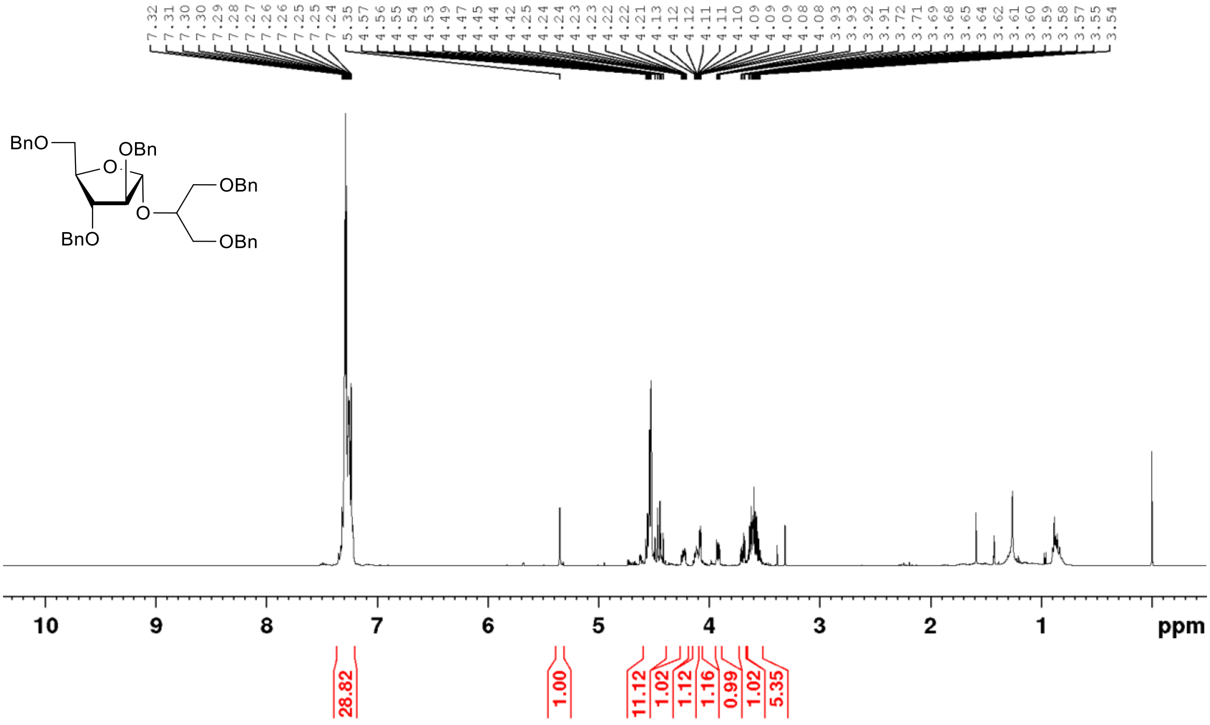

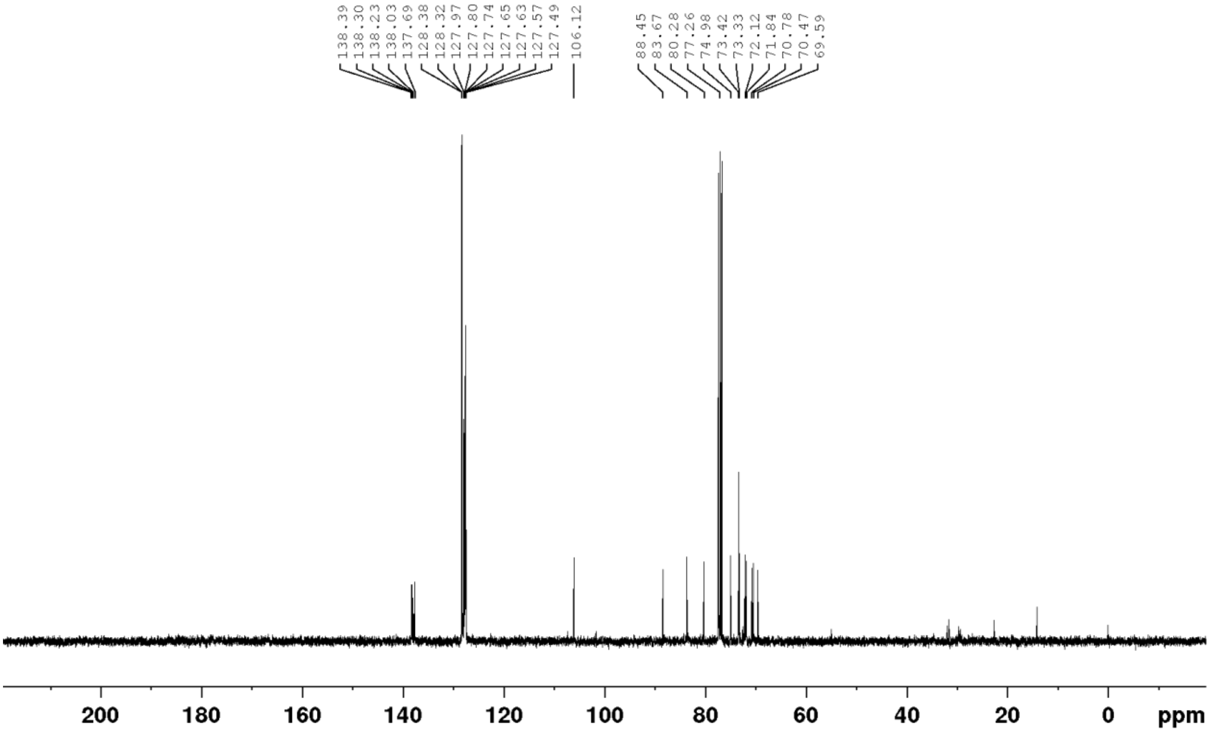

1,3-Dihydroxypropan-2-yl β-D-arabinofuranoside (5)

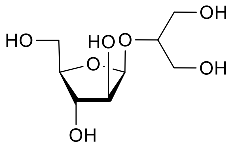

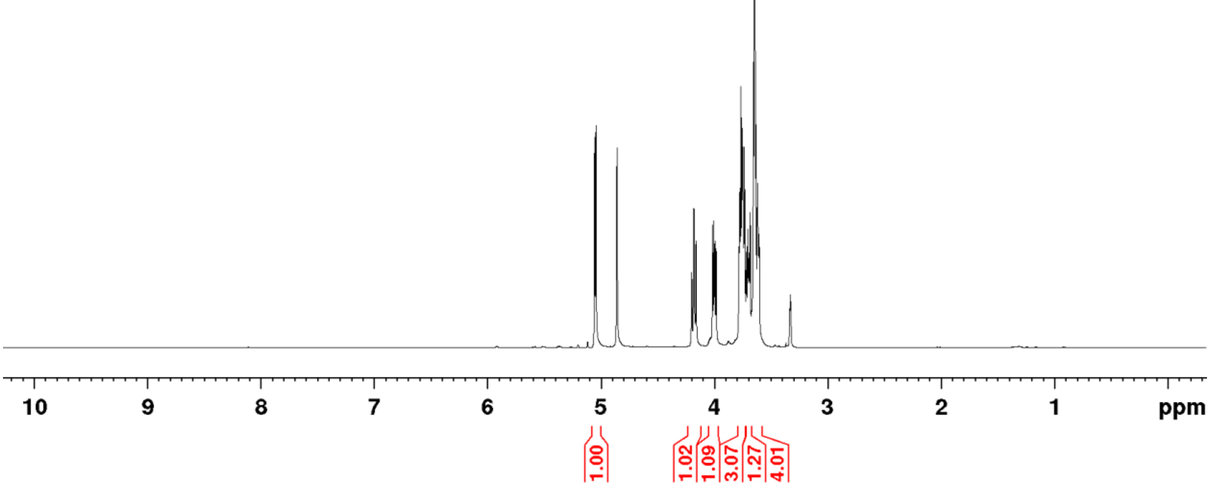

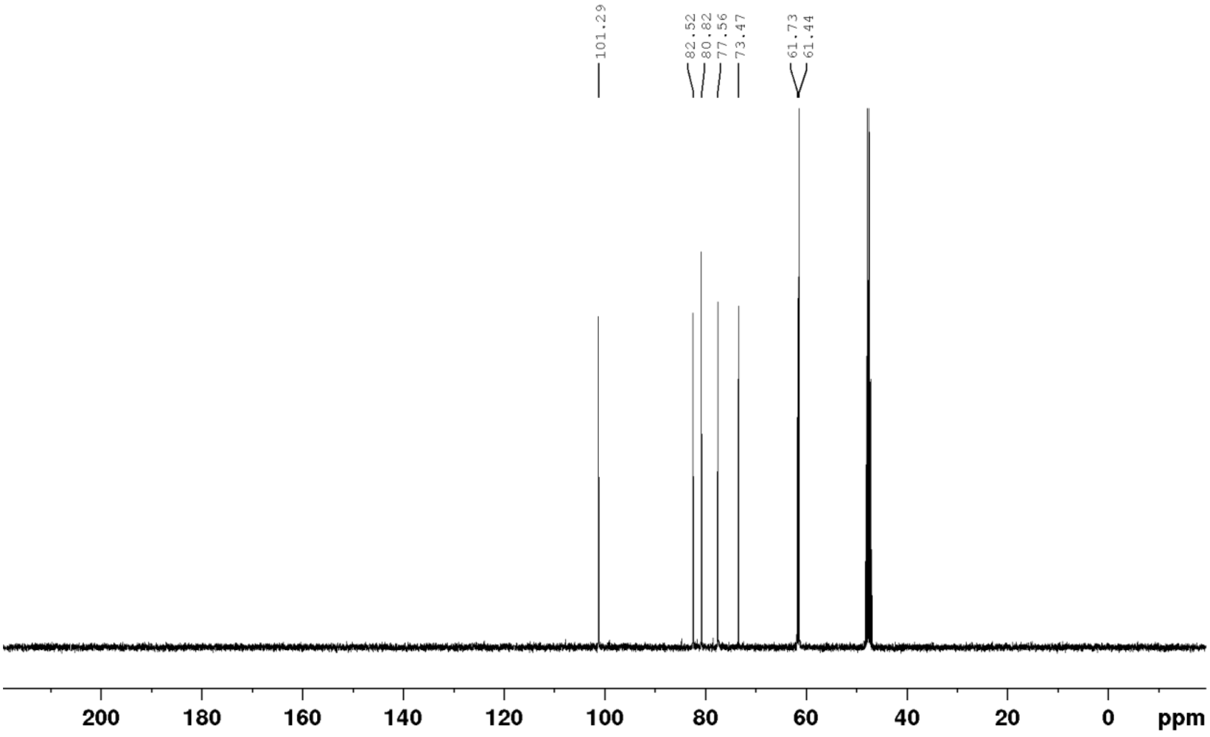

Prop-2-en-1-yl 2,3,5-tri-*O*-benzoyl-α-L-arabinopyranoside (22)

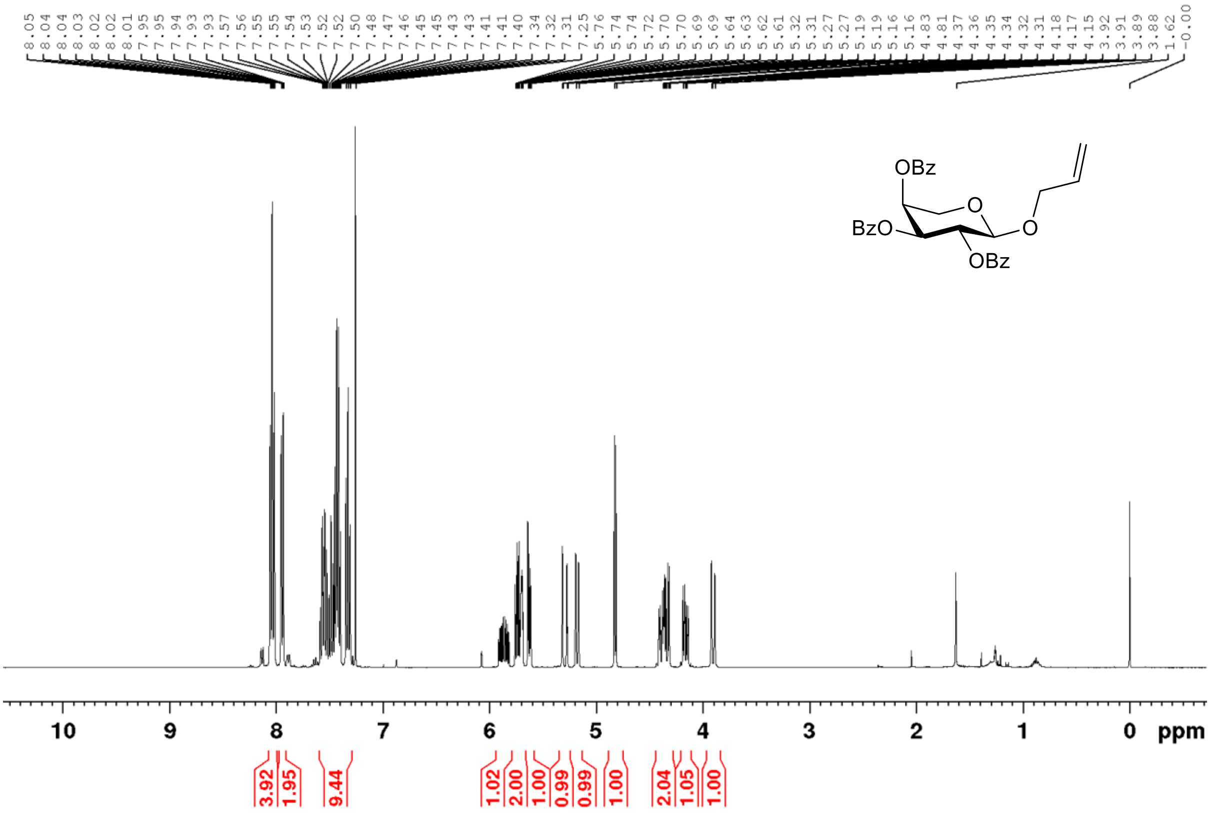

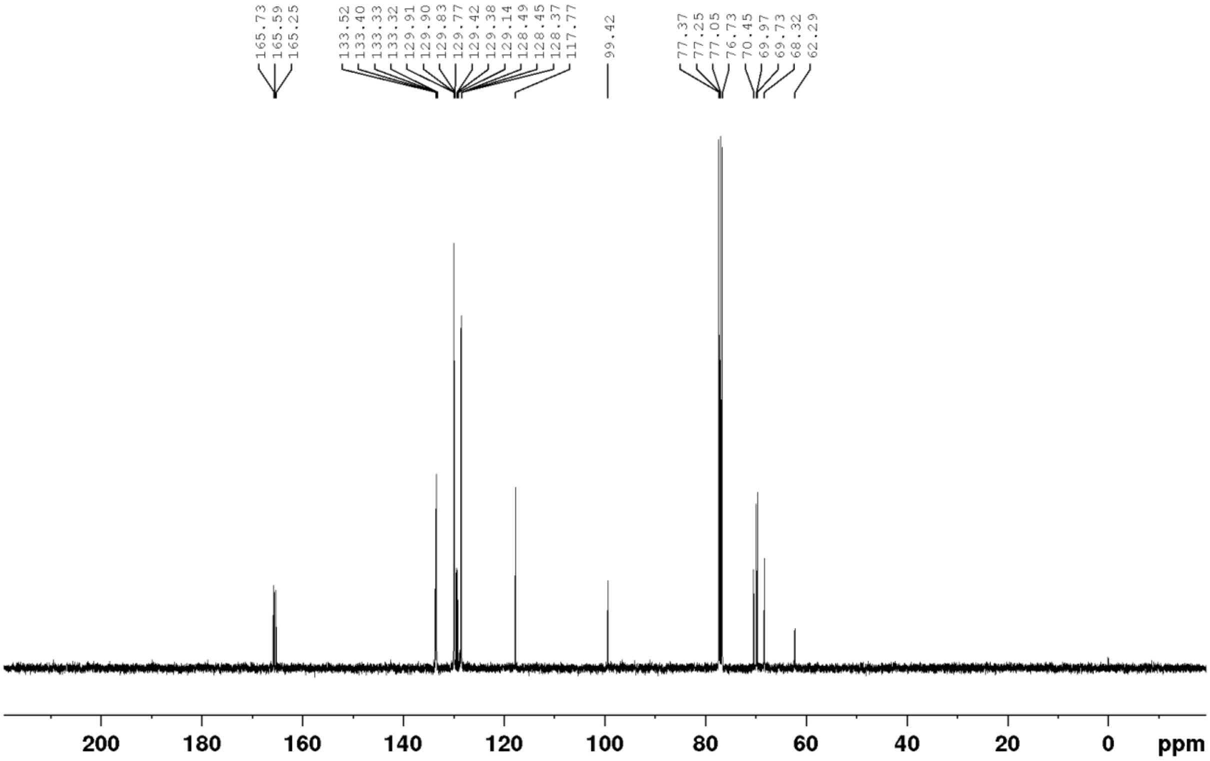

(*R*/*S*-Oxiranyl)methyl 2,3,5-tri-*O*-benzoyl-α-L-arabinopyranoside (23)

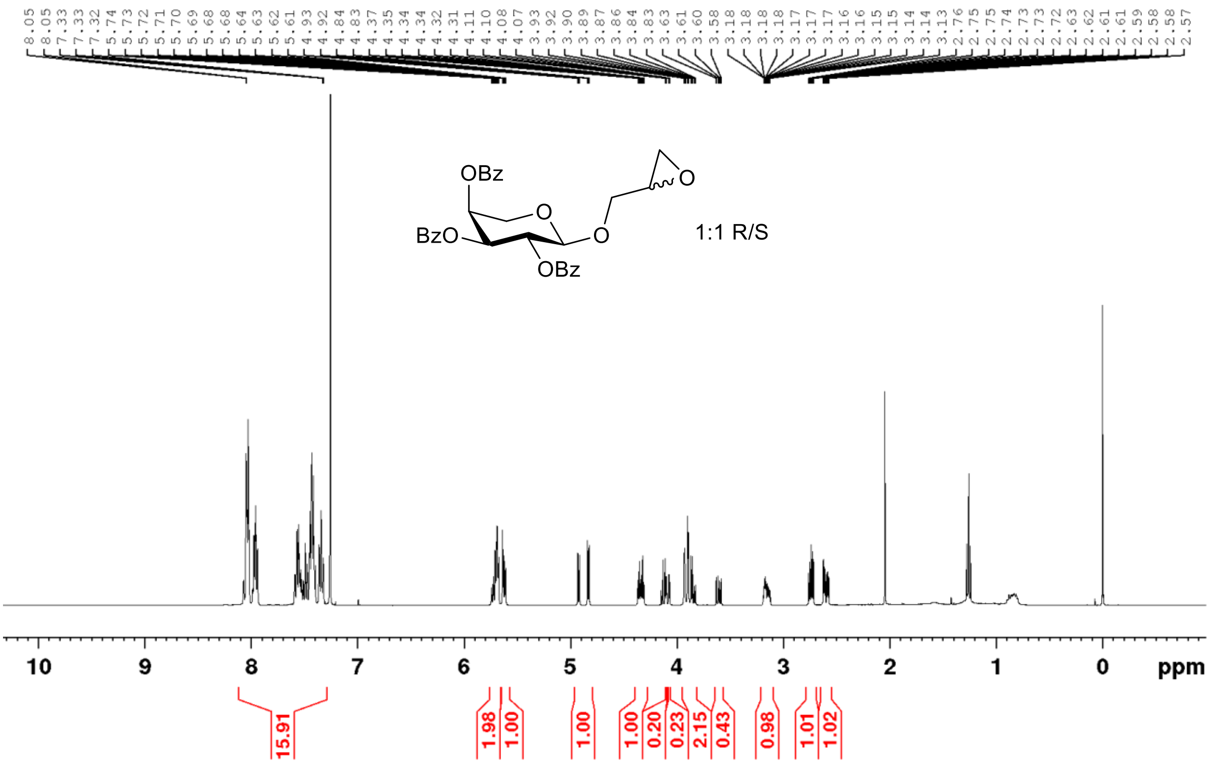

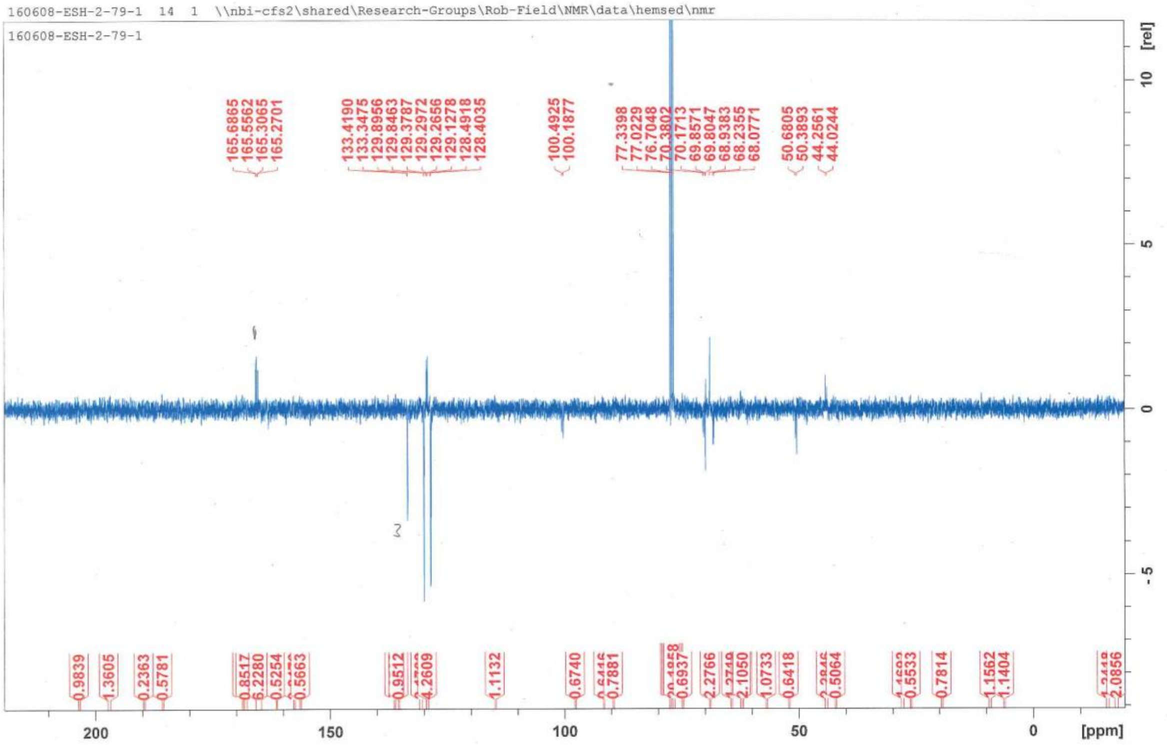

3-(3-Azidopropoxy)-2-hydroxypropyl 2,3,4-tri-*O*-benzoyl-α-L-arabinopyranoside (24)

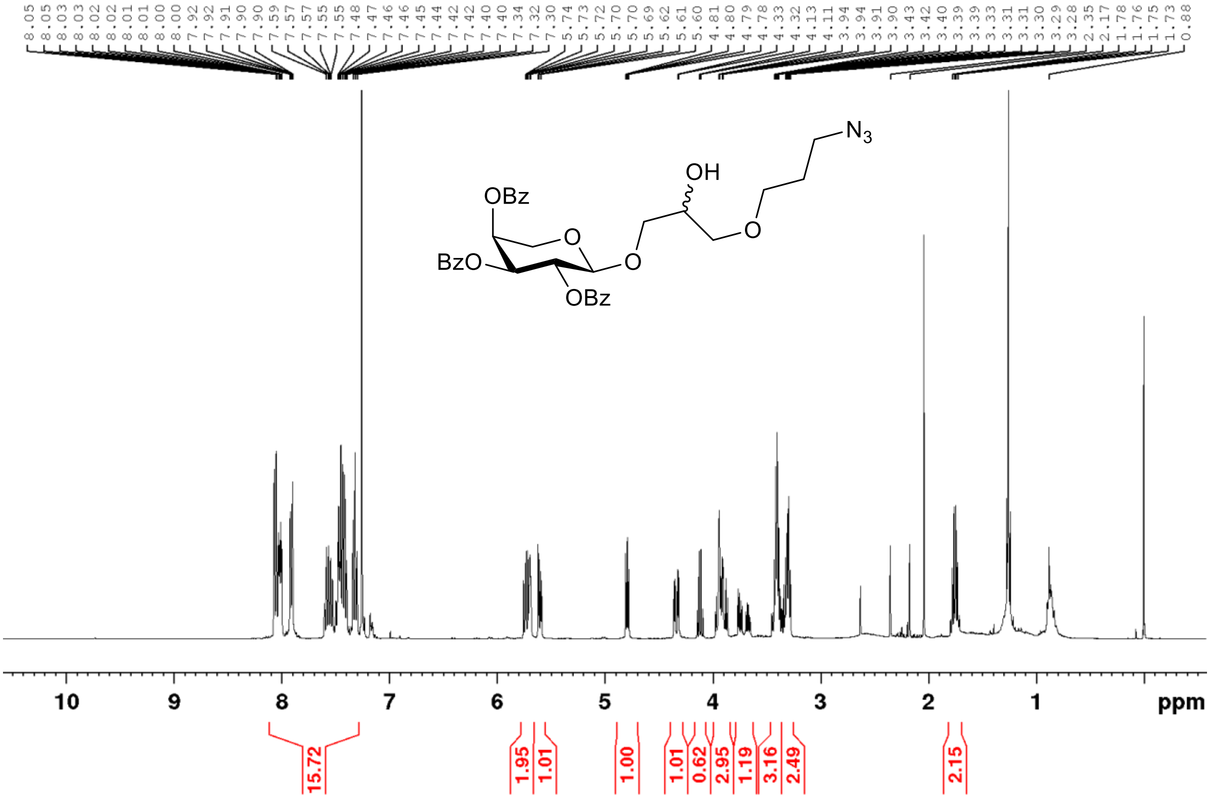

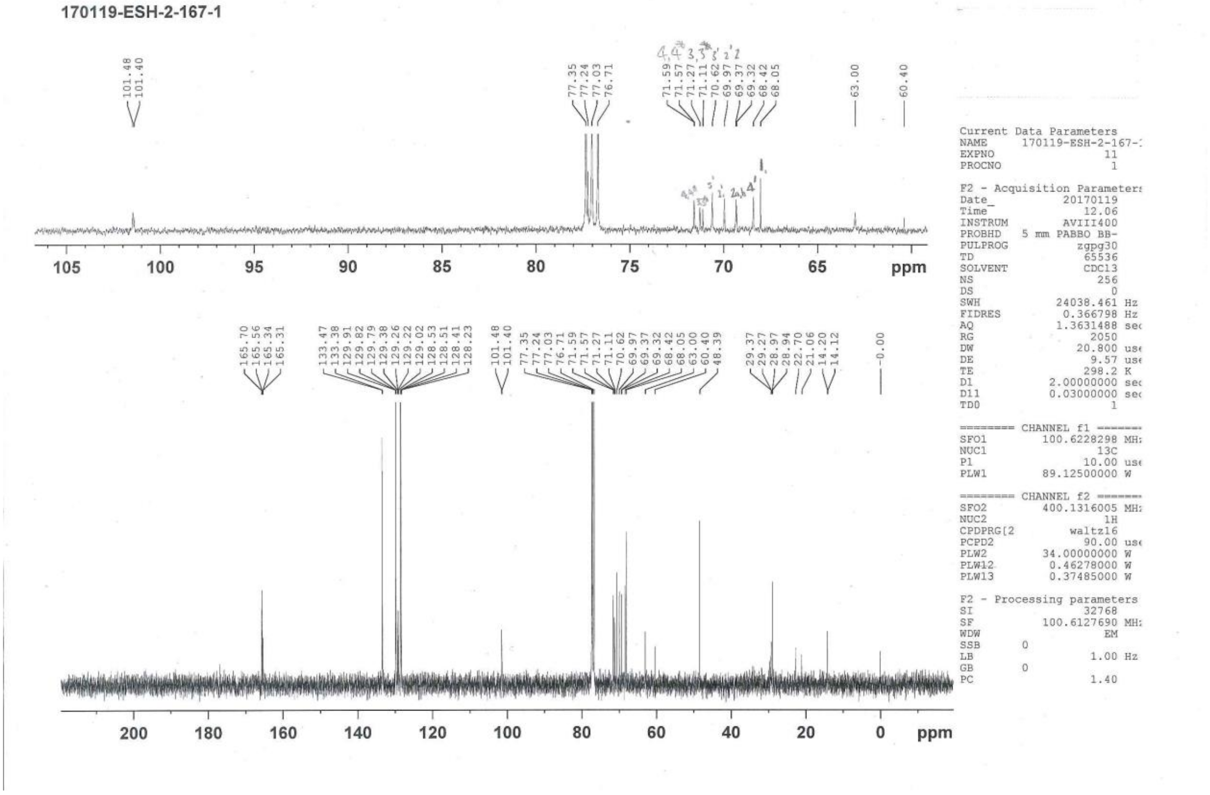

3-(3-Azidopropoxy)-2*R*-[(2,3,5-tri-*O*-benzyl-α-ᴅ-ribofuranosyl)oxy]propyl-2,3,4-tri-*O*-benzoyl-α- ʟ -arabinopyranoside (27)

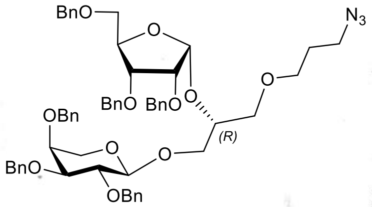

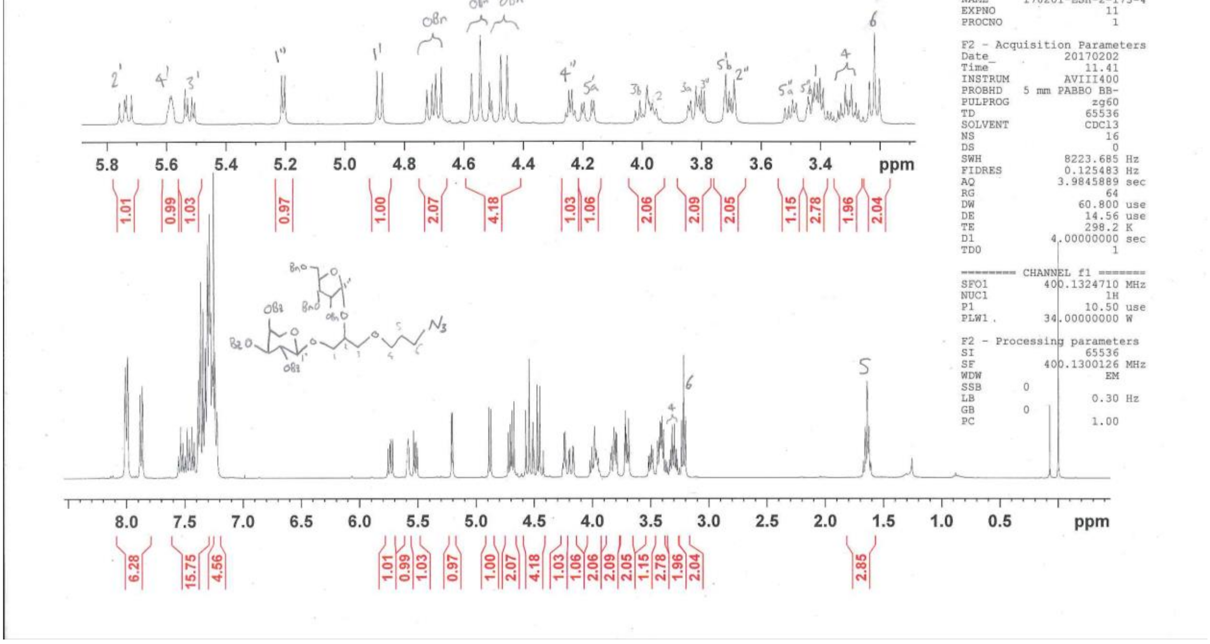

170201-ESH-2-173-4

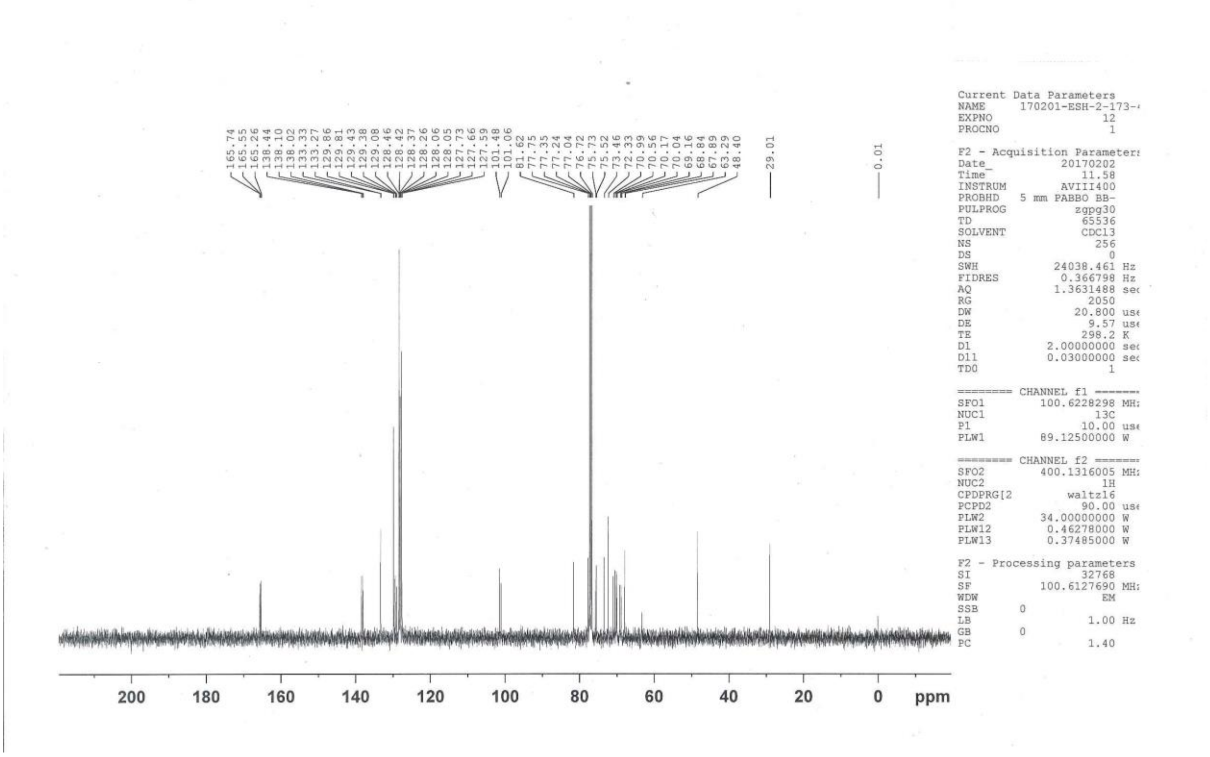

3-(3-Azidopropoxy)-2*S*-[(2,3,5-tri-*O*-benzyl-α-ᴅ-ribofuranosyl)oxy]propyl-2,3,4-tri-*O*-benzoyl-α- ʟ -arabinopyranoside (28)

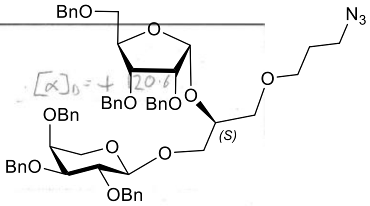

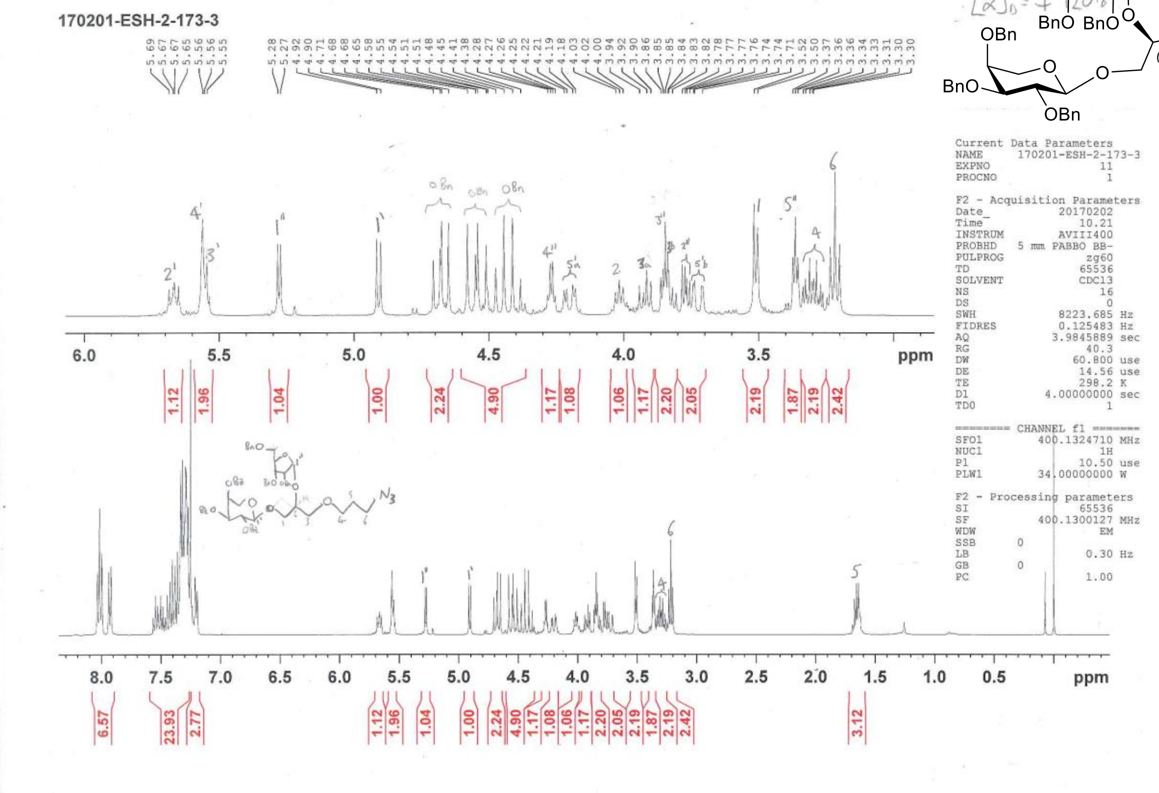

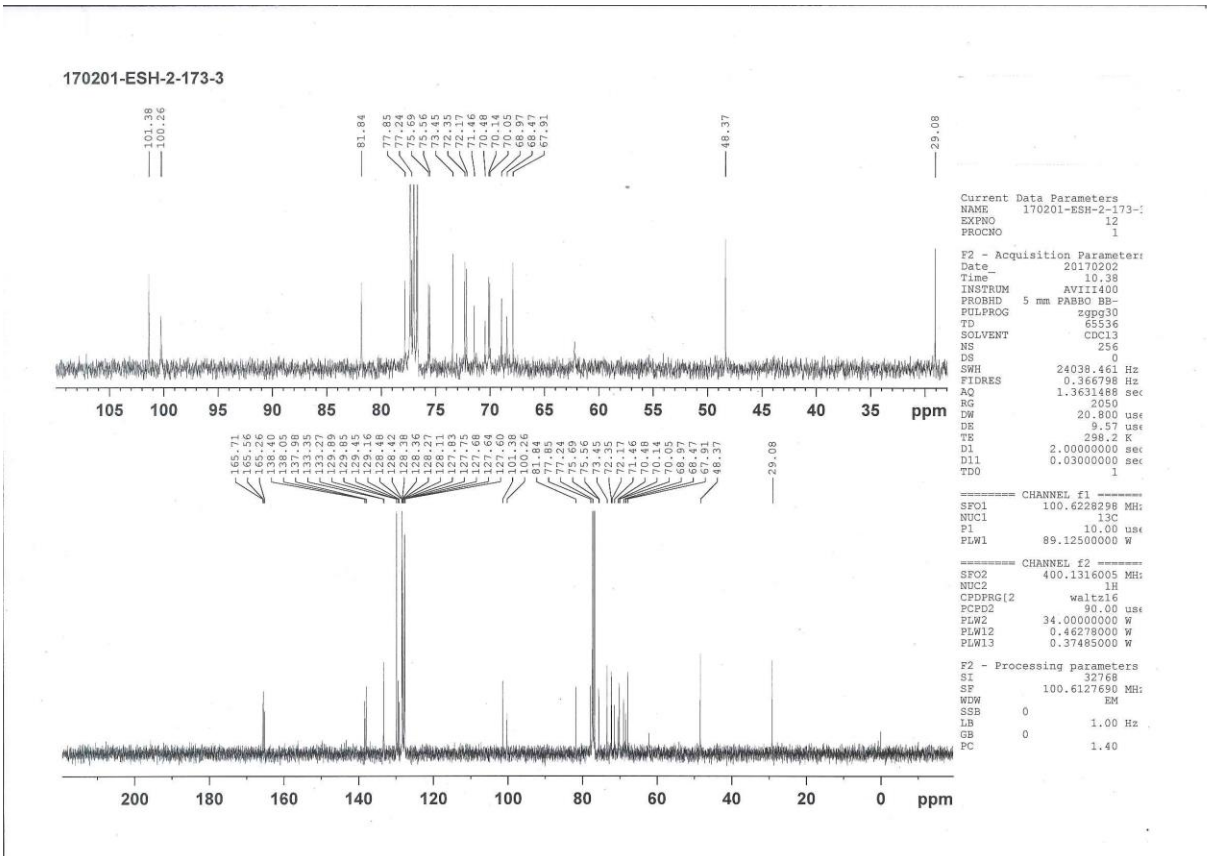

(2*R*)-3-(3-aminopropoxy)-2-(α-D-ribofuranosyloxy)propyl α-L-arabinopyranoside (6)

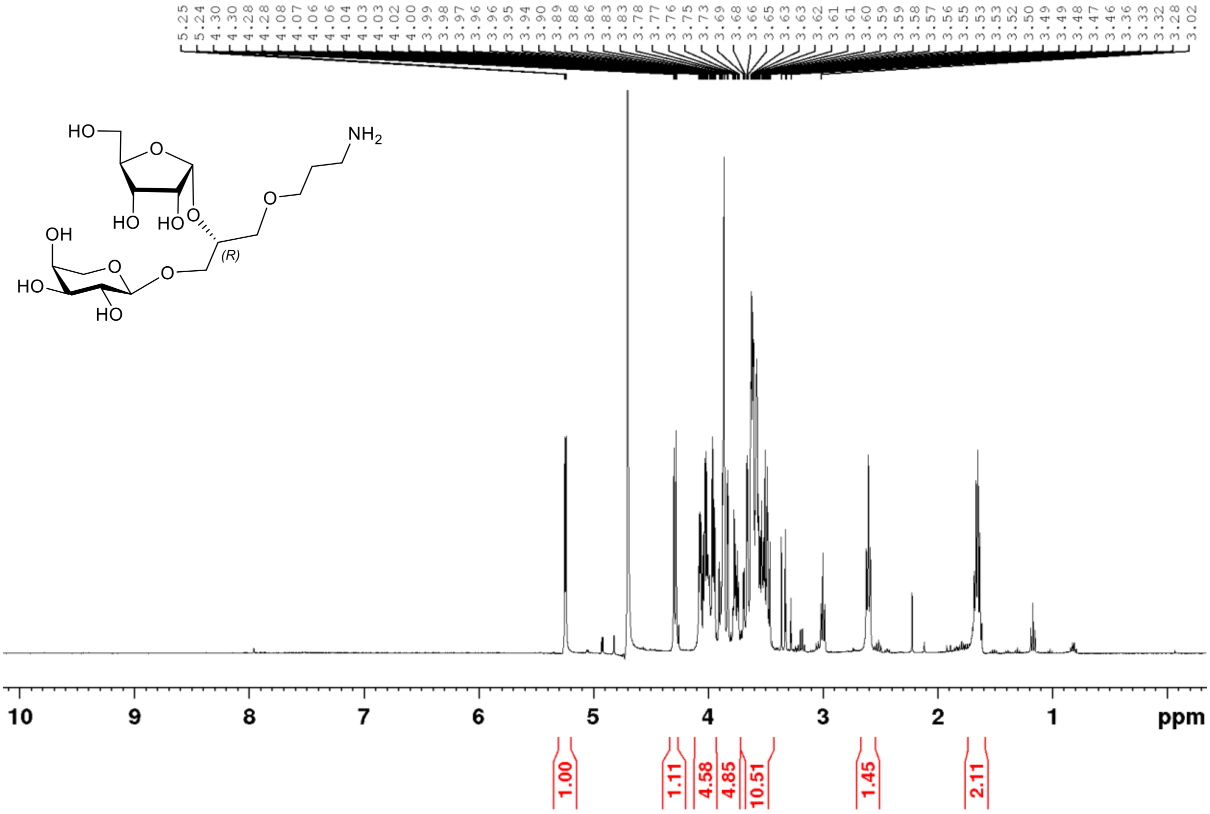

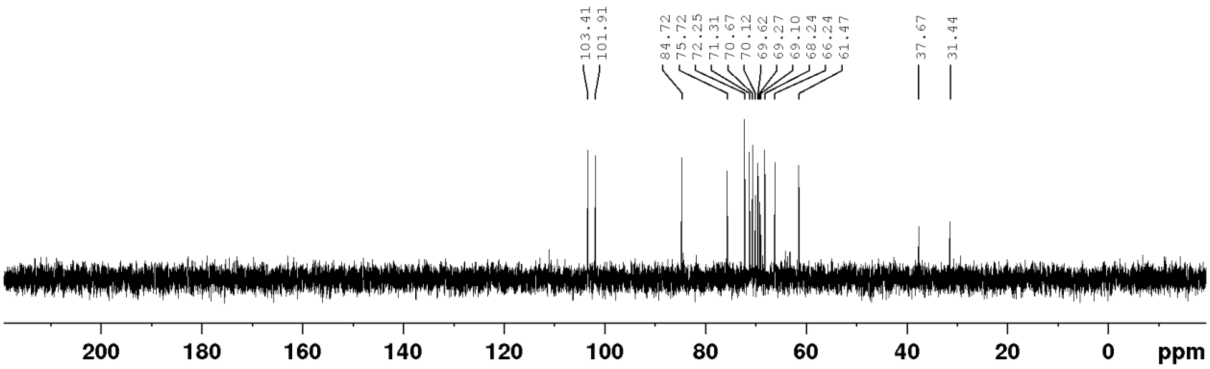

(2*S*)-3-(3-aminopropoxy)-2-(α-D-ribofuranosyloxy)propyl α-L-arabinopyranoside (7)

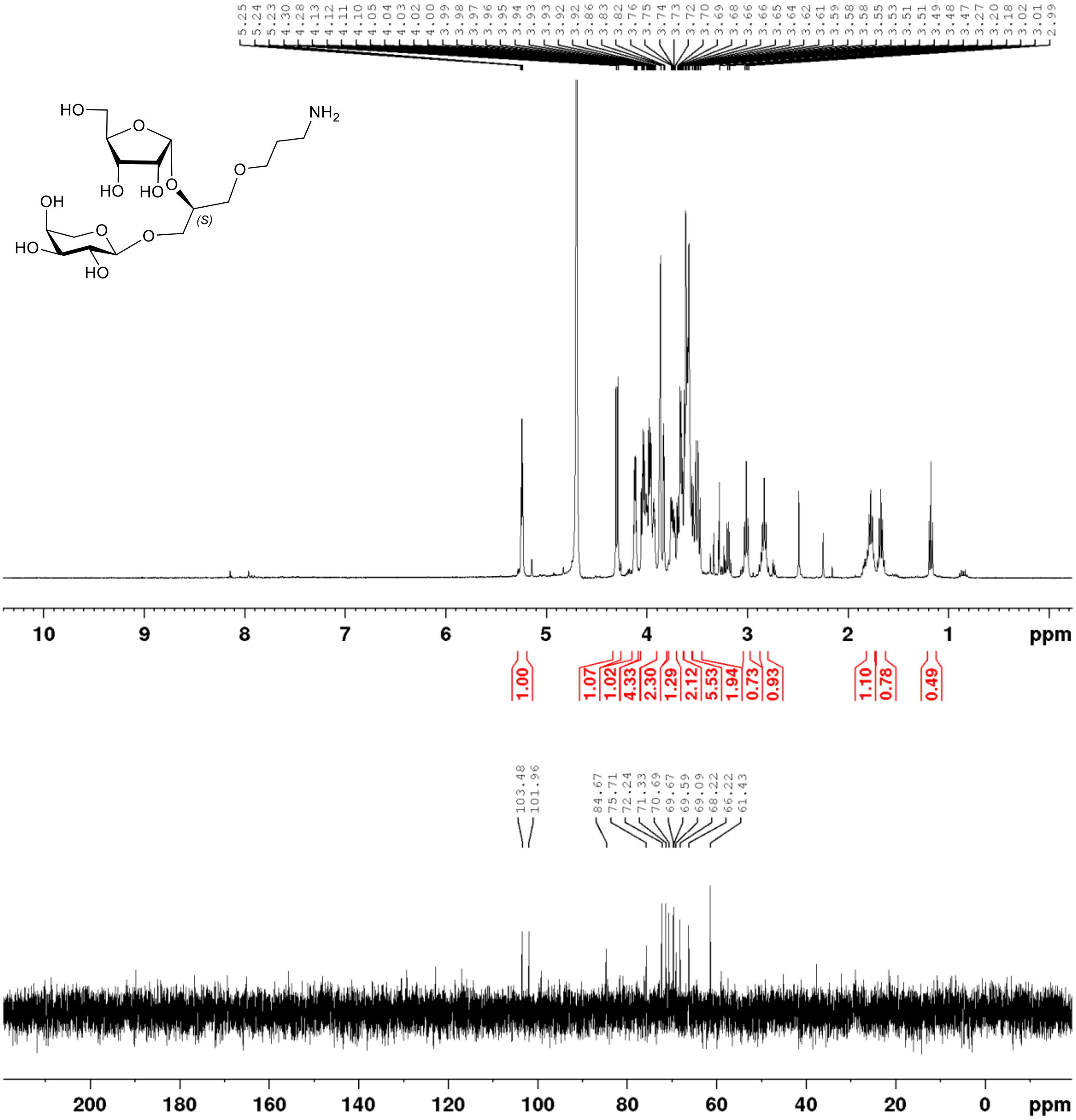

References:

[1] N.B. D'Accorso, I.M.E. Thiel, M. Schüller, Carbohydr. Res. 124 (1983) 177–184.

[2] Q. Zhang, H. Liu, J. Am. Chem. Soc. 122 (2000) 9065–9070.

[3] R.M. De Lederkremer, M.I. Litter, Carbohydr. Res. 20 (1971) 442–444.

[4] C. Gauthier, J. Legault, S. Lavoie, S. Rondeau, S. Tremblay, A. Pichette, Tetrahedron 64 (2008) 7386–7399.

[5] H. Dang, B.P. Roberts, J. Sekhon, T.M. Smits, Org. Biomol. Chem. 1 (2003) 1330–1341.

[6] W.R. Longworth, C.P. Mason, J. Chem. Soc. A Inorganic, Phys. Theor. (1966) 1164–1167.

[7] G.H. Posner, S.R. Haines, Tetrahedron Lett. 26 (1985) 5–8.

[8] T. Mukaiyama, Y. Hashimoto, S. Shoda, Chem. Lett. (1983) 935–938.

[9] W. Rosenbrook, D.A. Riley, P.A. Lartey, Tetrahedron Lett. 26 (1985) 3–4.

[10] G.J. Van Der Heden Van Noort, H.S. Overkleeft, G. a. Van Der Marel, D. V. Filippov, Org. Lett. 13 (2011) 2920–2923.
